# The elusive *endo*-product of the archetypal Diels–Alder reaction of furan and maleic anhydride – observed in the solid state at last

**DOI:** 10.1039/d5sc06724c

**Published:** 2026-05-13

**Authors:** Cameron B. Lennox, Christopher R. Taylor, Igor Huskić, Jogirdas Vainauskas, Christopher W. Nickels, Robin S. Stein, Tristan H. Borchers, A. R. Bonnie J. Lutton Gething, Joseph M. Marrett, Graeme M. Day, Tomislav Friščić

**Affiliations:** a School of Chemistry, University of Birmingham Molecular Sciences Building, Edgbaston Birmingham B152TT UK t.friscic@bham.ac.uk; b Department of Chemistry, McGill University 801 Sherbrooke St. West Montreal QC H3H 0B8 Canada; c School of Chemistry and Chemical Engineering, University of Southampton B30 Highfield Southampton SO17 1BJ UK g.m.day@soton.ac.uk

## Abstract

The [4 + 2] reaction of furan and maleic anhydride is an archetypal, textbook example of Diels–Alder reactivity, whose role as an early and prominent exception from the Alder *endo*-rule has made it a model in teaching and focus of continuous research for almost a century. Although the kinetics, thermodynamics and the *exo*-product of this reaction have been extensively studied, the critically important *endo*-product has remained almost completely unexplored, generally seen only as a fleeting intermediate in solution. By conducting the reaction without an external solvent, we now discover several solid forms of this historically important system, with the combined use of crystal structure prediction (CSP) and X-ray crystallography enabling the exploration of the structures and supramolecular chemistry of the *endo*-adduct in solidified reaction mixtures. Depending on the temperature, the *endo*-isomer appears to preferably form either a single-component solid or a cocrystal with the *exo*-isomer, which offers an explanation for reproducibility challenges reported 60 years ago and can tentatively be further rationalised through state-of-the-art free energy calculations. The observation and complexity of multiple new solid forms of the *endo*-, as well as *exo*-isomer, are surprising, long-missing contributions to this frequently visited Diels–Alder reaction.

## Abstract 2

Combining solventless reactivity, X-ray diffraction, crystal structure prediction and free-energy calculations enabled the discovery and characterisation of solid-state forms of the elusive *endo*-Diels–Alder product of maleic anhydride and furan.

## Graphical abstract

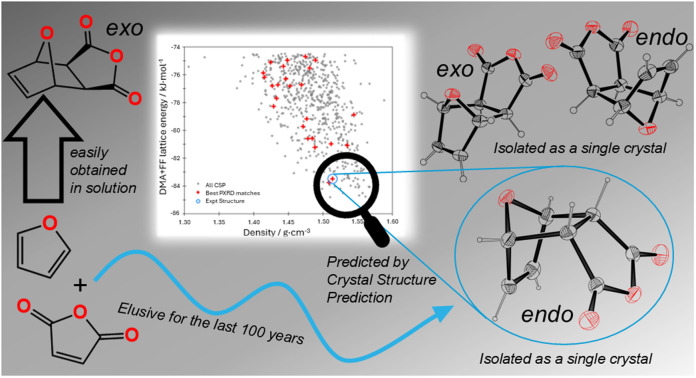

## Introduction

**Fig. 1:**
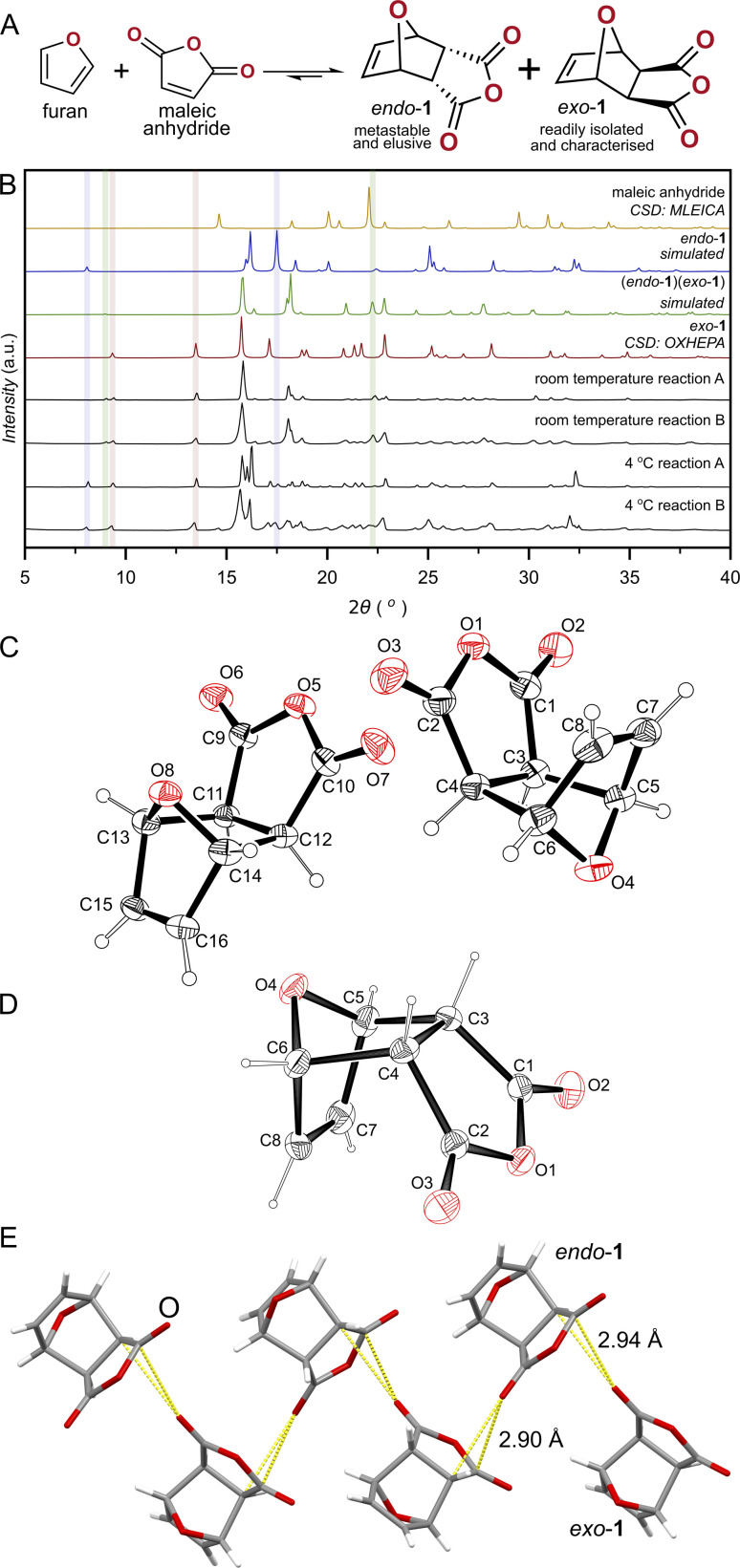
(A) Schematic representation of the Diels–Alder reaction of furan and maleic anhydride leading to *exo*- and *endo*-1; (B) powder X-ray diffractograms (top-to-bottom): simulated for single-crystal X-ray diffraction structure of maleic anhydride (CSD code MLEICA^27^), herein determined *endo*-1, herein determined (*endo*-1)(*exo*-1), *exo*-1 (CSD code OXHEPA^17^), measured for bulk product of the reaction between maleic anhydride and furan at room temperature (conducted at a scale of 30 mmol and 3 mmol), and at 4 °C (conducted at a scale of 30 mmol and 3 mmol). Characteristic Bragg reflections for *endo*-1, (*endo*-1)(*exo*-1) and *exo*-1 are shown in blue, green, and red, respectively. ORTEP views of the asymmetric unit contents for the herein determined crystal structures of: (C) (*exo*-1)(*endo*-1) cocrystal and (D) *endo*-1. Non-hydrogen atoms are shown as thermal ellipsoids corresponding to 30% probability of electron density. (E) Chains of alternating *endo*-1 and *exo*-1 molecules in the (*endo*-1)(*exo*-1) cocrystal with yellow dotted lines highlighting the short C⋯O contacts (with interatomic distances indicated) and C–H⋯O hydrogen bonds.

The Diels–Alder [4 + 2] cycloaddition^1–4^ is among the most important organic transformations, of high historical importance as a key reaction in the study of pericyclic reactions and the emergence of a molecular orbital theory of reactivity. This type of cycloaddition was first reported by Diels and Alder in a series of papers published between 1928 and 1934,^5^ which described a number of reactions that subsequently became textbook material and the subject of extensive experimental and theoretical studies. Among such archetypal examples of Diels–Alder reactivity is the reaction of furan^6^ with maleic anhydride,^7^ described in 1929 as a part of Diels and Alder's second report on such reactivity (Fig. 1A).^8–11^ This particular reaction continues to be the subject of much experimental and theoretical interest, and a model system in teaching experiments,^12–15^ because of its selectivity to form the adduct of *exo*-stereochemistry (*exo*-1) which contrasts the *endo*-stereochemistry of the analogous cyclopentadiene adduct, and the empirical *endo*-rule^12^ of Diels–Alder chemistry. While the original work of Diels and Alder anticipated the *endo*-stereochemistry (*endo*-1) for reaction of maleic anhydride and furan, the *exo*-stereochemistry was established by Woodward and Baer in 1948 ^16^ and confirmed several times by X-ray crystallography by Baggio and co-workers in 1972,^17^ as well as the White (2008),^18^ and the Pinkerton^19^ groups.

The difficulty obtaining *endo*-1 has attracted kinetic studies that have sometimes provided conflicting results. For example, Lee and Herndon have indicated that the initial rate of formation for *endo*-1 is *ca.* 500 times higher compared to that of *exo*-1,^20^ with the final isolated product being the result of rapid *endo-*to *exo-*1 conversion. Koreshkov *et al.*^21^ have found that the rates of formation of *endo*- and *exo*-1 are comparable, while González and López-Ortiz have also observed the preferred formation of *endo*-1.^22^ More recently, the Svatoš group has observed comparable initial rates of formation for both isomers, with *endo*-1 subsequently converting into the *exo*-form.^23^ Overall, the current experiment-based literature indicates that both *endo*- and *exo*-1 should be of similar abundance in early stages of the reaction, with a retro-Diels–Alder process driving the subsequent formation of *exo*-1 as the final product. Gas-phase theoretical calculations over the past decades generally indicate that the *exo*-isomer should be between *ca.* 2 and 3 kcal mol^−1^ (*ca.* 8–12 kJ mol^−1^) more stable compared to *endo*-1.^18–26^

The thermodynamic preference for *exo*-1, coupled with the low activation energy barrier for the cycloreversion of *ca.* 25 kcal mol^−1^,^10^ has made *endo*-1 tantalizing to isolate and generally absent from experimental research. In contrast to the *exo*-isomer, which is readily isolated from solution and was characterised extensively and by multiple X-ray structural analyses, mentions of *endo*-1 as anything else but a fleeting species in solution are scarce.

Specifically, in 1962 ^28^ Anet reported that repeated addition of petroleum ether to a solution of furan and maleic anhydride in acetone produces several solid fractions, one of which was identified as *endo-*1, based on ^1^H NMR analysis. Intriguingly, this work also noted that “Later attempts to isolate the pure endo isomer were thwarted by the tendency of 1 : 1 complex of the *endo* and *exo* isomers to crystallize preferentially”. In 1973, Eggelte and co-workers^29^ reported the targeted synthesis of *endo*-1 through dehydration of the corresponding diacid, providing a solid that melted with decomposition at *ca.* 80 °C, and in solution quickly transformed into *exo*-1. In 2008, the White group attempted to obtain solid *endo*-1 by crystallisation at −20 °C but without success.^18^ The lack of structural characterisation of *endo*-1 is even more striking considering that the supramolecular complex leading to this adduct has recently been characterised in a pulsed jet.^30^ Overall, since the original work of Diels and Alder, solid *endo*-1 appears to have remained elusive and almost completely unexplored by any other technique except solution ^1^H NMR, making it a striking example of a textbook molecule whose structural characterisation has remained largely theoretical.

Computational methods for organic molecular crystal structure prediction (CSP)^31,32^ have had an important impact in solid-state chemistry, demonstrating predictive and explanatory power in applications from porous materials^33^ to alternative semiconductors.^34^ The outstanding reliability of CSP methods for small, rigid molecules^35^ invites their use here to address the behavior of *exo*-1 and *endo*-1. Truly predictive thermodynamic information from computational methods often requires computationally expensive techniques, further limiting the extent to which we can fully explore the “CSP landscape”, *i.e.*, possible minima on the crystalline potential energy surface. However, high-level calculations on subsets of the predicted structures, or even on experimentally observed structures alone, can provide useful insight. The large-scale application of solid-state density functional theory (DFT) to known structures has elucidated the thermodynamics of cocrystallisation,^36^ while sophisticated free-energy calculations have evaluated the typical relative stability of observed polymorphs and how these vary with temperature.^37^ While the expense of such approaches often precludes their application to entire CSP landscapes, informed analysis of the landscapes can permit identification of the most important or relevant structures to prioritise them for more intensive calculations.

We now present the first structural investigation of *endo*-1 in the solid state, achieved by combining a detailed experimental investigation of the solventless Diels–Alder reaction products with CSP, solid-state DFT and free energy calculations. Whereas the solventless reaction was previously reported to yield *exo*-1,^38,39^ we found it generates at least three additional crystalline phases, including solid *endo*-1, a cocrystal of *endo*-1 and *exo*-1, as well as a cocrystal of *exo*-1 with the reactant furan. Moreover, the composition of the product mixture was found to change depending on reaction temperature, with higher temperature preferring the formation of the (*endo*-1)(*exo*-1) cocrystal over individual *exo*-1 and *endo*-1 phases. While this might explain the irreproducibility reported by Anet,^28^ a thorough understanding of this behavior required the synergistic application of experimental and theoretical approaches, with CSP combined with experimental powder X-ray diffraction (PXRD) information enabling the accurate prediction of the crystal structure of pure *endo*-1, while computed free energy differences provide an explanation of the herein observed temperature-dependent preference for (*exo*-1)(*endo*-1) cocrystal formation.

## Results and discussion

### Cocrystal of *endo*-1 and *exo*-1

Our observation of the solid forms of *endo*-1 was serendipitous, resulting from exploring how a solvent-free methodology to prepare the *exo*-isomer can be implemented as a part of a Green Chemistry undergraduate course. Mixing of equimolar amounts of solid maleic anhydride and liquid furan at room temperature led to dissolution of the solid and formation of a liquid phase that subsequently crystallised. After 20–40 minutes sitting on the bench, the reaction mixture is found to be completely solidified. While previous work indicated that solventless reaction should lead to the *exo*-adduct,^38–40^ PXRD analysis of a sample of the reaction mixture after standing overnight revealed signals additional to those anticipated from the published X-ray crystal structure of *exo*-1 (previously reported six times, with Cambridge Structural Database (CSD)^41^ codes: OXHEPA,^17^ OXHEPA01,^42^ OXHEPA02,^18^ OXHEPA03,^19^ OXHEPA04,^43^ OXHEPA05,^43^ or OXHEPA06 ^43^). The additional X-ray reflections also did not match those of either maleic anhydride^27^ or maleic acid that might have formed due to the presence of air moisture (Fig. 1B). The ^1^H NMR analysis of an extracted sample of the product dissolved in CD_3_CN revealed two major sets of signals, one corresponding to *exo*-1, while the second one was consistent with chemical shifts reported for *endo*-1.^28,29^ In addition to these signals, which indicated a ratio of *endo*-1 and *exo*-1 of *ca.* 2 : 7, the ^1^H NMR spectrum also exhibited resonances consistent with small amounts of furan and maleic anhydride, *ca.* 2% each (see SI). In order to avoid potential sampling effects, the reaction was also conducted at a smaller scale (3 mmol), which enabled the immediate PXRD analysis of the entire reaction mixture, with similar results (see SI).

Gratifyingly, we found that the solid product of the neat reaction consisted of micrometer-sized crystals that could be picked individually and explored using single crystal X-ray diffraction. Most crystals studied had unit cell parameters consistent with the structure of *exo*-1 (Table 1), but one revealed a set of different unit cell parameters, *a* = 11.9547(5) Å, *b* = 5.4149(2) Å, *c* = 12.0064(5) Å. Subsequent crystal structure analysis revealed that the material was a cocrystal of the two isomers, with the composition (*endo*-1)(*exo*-1). The asymmetric unit of (*endo*-1)(*exo*-1) contains one molecule of *exo*-1 and one molecule of *endo*-1, previously never experimentally observed in the solid state (Fig. 1C). In the cocrystal structure, alternating *endo*-1 and *exo*-1 molecules connect into chains *via* short C⋯O contacts involving the anhydride carbon atom of the molecule of one isomer and a carbonyl oxygen atom from a neighboring molecule of a different isomer. These C⋯O separations are 2.94 Å (oxygen atom of an *exo*-1 molecule to a carbon atom on an *endo*-1 molecule) and 2.90 Å (oxygen atom of an *endo*-1 molecule to a carbon atom on an *exo*-1 molecule). In both cases the distances are shorter than the expected sum of van der Waals radii of oxygen and carbon (3.32 Å),^44^ reminescent of short contacts seen, for example, in the crystal structure of alloxan.^45^ The oxygen atom in each of these short contacts is also involved in a C–H⋯O hydrogen-bonding contact with the neighboring molecule, with C⋯O separations of 3.04 Å and 3.05 Å. The formation of (*endo*-1)(*exo*-1) was observable for more than one single crystal, as confirmed by X-ray diffraction experiments on other manually-picked crystals. Quantitative Rietveld analysis of a PXRD pattern collected on a sample from a reaction conducted at 3 mmol scale indicated relative weight fractions of *exo*-1 and (*endo*-1)(*exo*-1) of 44% and 56%, respectively. These weight fractions correspond to relative amounts of *endo*-1 and *exo*-1 of *ca.* 2 : 5, which matches reasonably well to the above mentioned ^1^H NMR analyses of entire reaction mixtures dissolved in CD_3_CN.

**Table 1:** Crystallographic parameters for different reports of the crystal structures of *exo*-1, the presently reported (*endo*-1)(*exo*-1) (shown for two different crystals), the best prediction of our *ab initio* CSP procedure for *endo*-1 compared to PXRD data, and the presently reported solid *endo*-1 (shown for two different crystals)

Parameter	*exo*-1	*exo*-1	(*endo*-1)(*exo*-1)^a^	(*endo*-1)(*exo*-1)^a^	*endo*-1 CSP	*endo*-1^b^	*endo*-1^b^
CSD code	OXHEPA^17^	OXHEPA03^19^					
*T* (K)	Room temperature	20	298	298	[N/A]	291	200
Space group	*P*2_1_2_1_2_1_	*P*2_1_2_1_2_1_	*Pn*	*Pn*	*P*2_1_/*c*	*P*2_1_/*c*	*P*2_1_/*c*
*a* (Å)	7.0(2)	5.3018(1)	11.9551(6)	11.9547(5)	11.0512	11.2681(11)	11.2234(8)
*b* (Å)	18.93(3)	6.9265(1)	5.4120(3)	5.4149(2)	5.6988	5.7140(6)	5.6672(3)
*c* (Å)	5.38(1)	18.6795(5)	12.0034(7)	12.0064(5)	11.3996	11.4194(13)	11.3510(8)
*b* (^o^)	—	—	110.570(3)	110.600(2)	103.773	103.858(11)	103.917(7)
*V* (Å^3^)	712.904	685.966	727.12(7)	727.52(5)	697.29	713.85(13)	700.79(8)
*Z*	4	4	2	2	4	4	4
*r*_calc (_g cm^−3^)	1.56	1.609	1.518	1.517	1.583	1.546	1.575

a Unit cell data collected on two different crystals of (*endo*-1)(*exo*-1).

b Unit cell data collected on two different crystals of *endo*-1.

Conducting the neat Diels–Alder reaction of furan and maleic anhydride by mixing pre-cooled reactants until dissolution, and then placing the mixture overnight in a laboratory refrigerator set at 4 °C, also led to the reaction mixture solidification and formation of a colourless polycrystalline product. PXRD analysis of a sample extracted from the solid reaction mixture showed Bragg reflections of *exo*-1 and yet another, unidentified, material alongside small amounts of (*exo*-1)(*exo*-1) (Fig. 1B, also SI). Analysis of an extracted sample by ^1^H NMR spectroscopy in CD_3_CN solution revealed two sets of signals that were consistent with previously reported spectra for *exo*-1 and *endo*-1, as well as maleic anhydride and furan. Conducting this process on a smaller (3 mmol) scale, again with the reaction mixture at 4 °C, provided similar results, with improved sampling in this case showing a more prominent presence of maleic anhydride in the PXRD and NMR analyses. While the stoichiometric ratio of *exo*-1 to *endo*-1 was generally close to 2 : 1 (see SI), the NMR analysis showed a significant difference in the relative amounts of remaining maleic anhydride (29% and 24% in duplicate experiments) to furan (9% and 5% in duplicate experiments). The difference can be explained by the high volatility of the latter (boiling point 30 °C), leading to its rapid evaporation during sample handling and preparation. The presence of unreacted maleic anhydride and lower extent of the reaction when held at 4 °C is likely to be affected by different crystallisation behavior compared to reactions held at room temperature: PXRD analysis showed that the first solid separated upon standing of the reaction mixture at room temperature was largely *exo*-1. In contrast, the first solid separating at 4 °C was found to be maleic anhydride (see SI).

Overall, the combined PXRD and NMR analyses indicate that the unidentified phase from a low-temperature crystallisation process also contains *endo*-1. The formation of this phase appears to be promoted when the reaction mixture is left to crystallise at low temperatures.††All ^1^H NMR spectra have been recorded within 10 minutes of sample dissolution in CD_3_CN. A time-resolved ^1^H NMR study of the sample solution shows that the relative content of *exo*-1 in the mixture of two isomers changes by *ca.* 5% within 20 minutes after the first spectrum was measured (see SI). Similar behavior was observed regardless of whether commercial maleic anhydride was used directly, or whether it was purified by sublimation (see SI), as well as for the reactions crystallising at 0 °C (cooled using a water-ice bath) and −11 °C to −12 °C (with the reaction placed in a freezer or cooled using a flow cryostat).

### Crystal structure prediction and experimental observation of solid *endo*-1

In anticipation that the third, unidentified phase in the solidified Diels–Alder reaction mixture might be the elusive *endo*-1, we undertook a CSP study of the *endo*-1 molecule. Our global lattice energy explorer^46^ software was used to generate hypothetical crystal structures using the gas-phase-optimised *endo*-1 molecular geometry in 25 commonly observed space groups for organic molecular crystals. These candidate structures were energy-minimised using a combination of distributed atomic multipoles derived from quantum mechanical charge densities^47^ and a revised version of the W99 empirical atom–atom repulsion-dispersion potential (the W99rev force field),^48–51^ henceforth referred to as the DMA + FF level of theory. For details of our CSP workflow, see SI S10. Computational details.

**Fig. 2:**
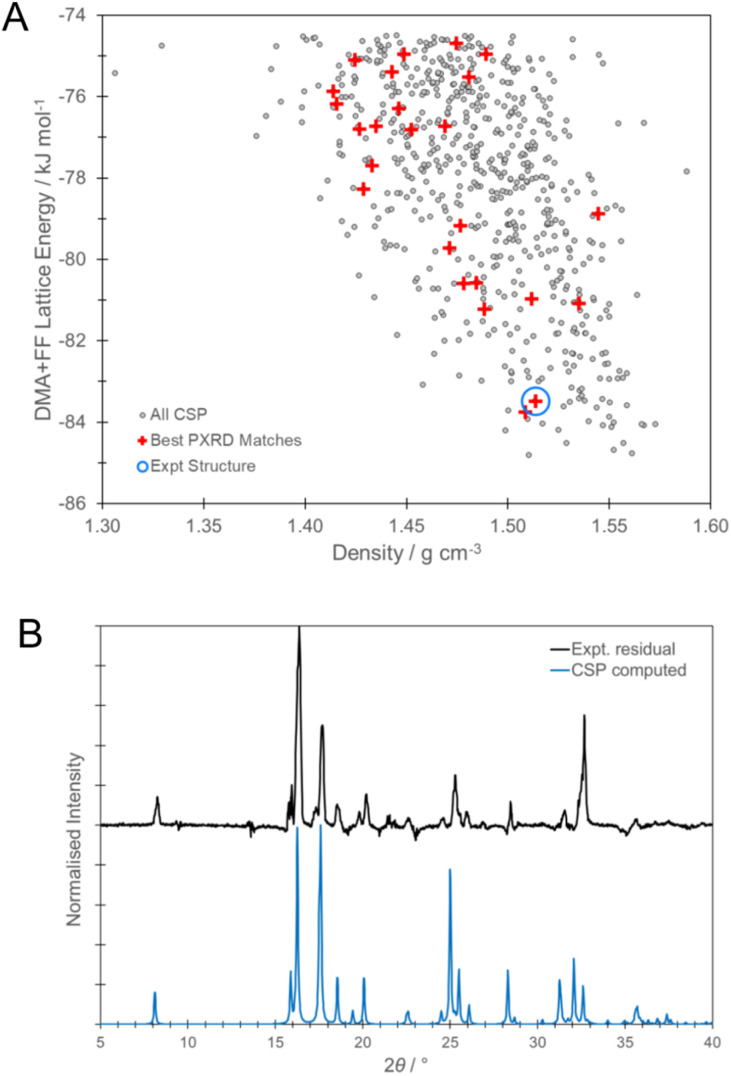
(A) The CSP landscape for pure *endo*-1 at the DMA + FF level of theory. All points represent hypothetical structures from our *ab initio* CSP procedure. Red crosses correspond to structures showing reasonable and consistent agreement with the residual powder X-ray diffraction (PXRD) pattern (B) (black line) obtained in the process of isolating the pattern of pure *endo*-1 (derived by subtracting the patterns for pure *exo*-1 and the (*endo*-1)(*exo*-1) cocrystal from the pattern of the low-temperature neat reaction mixture). The blue pattern in (B) is the computed PXRD pattern of the CSP-derived structure (circled in blue in (A)) that gives the overall best match to the residual pattern after further refinement and re-ranking (Fig. 3). This was found to be an excellent structure match for the pure *endo-*1 form once the latter was isolated. The intensities of the two PXRD patterns have been scaled separately (highest intensity peak in each pattern set to 1.0) and vertically offset for clarity. Negative intensities in the residual (black line) are artifacts of its construction (as the element-wise vector difference between experimental patterns).

This CSP procedure yielded an unusually populous landscape (Fig. 2A) of possible crystal structures, with more than 600 predicted structures lying within 10 kJ mol^−1^ of the global minimum energy structure. This energy window represents a conservative estimate of the error in energetic ranking between this approximate energy model and periodic DFT rankings^52^ (among the most accurate possible and thus the gold standard for CSP), but due to such a populous landscape, complete DFT re-optimisation and re-ranking of all energetically-relevant structures was not feasible. Instead, predicted structures were assessed against a residual PXRD pattern (see Fig. 2B, also SI) obtained from the difference between the patterns of the solidified reaction mixture and the patterns calculated for *exo*-1 and (*endo*-1)(*exo*-1) cocrystal.

For comparison, we calculated simulated PXRD patterns for each of the CSP structures (using the PLATON package^53^) and compared them to the residual pattern using a constrained dynamic time-warping (cDTW) algorithm,^54^ which allows for shifts in peak positions during comparison. The CSP results were filtered based on similarity to this residual, under the assumption that it was an accurate representation of the pure *endo*-1 pattern. Details of the comparison and selection process are presented in the SI; in brief, we selected all structures that were ranked among the 10 closest matches to the residual pattern at any reasonable value of the cDTW constraint. Using this relatively indiscriminate approach, we reduced the candidate population from *ca.* 640 to only 25 unique structures classified as good potential PXRD matches. This filtering process, reliant on experimental PXRD data to generate a realistic residual, thus reduced the number of relevant potential matches to a set that could be subjected to full periodic DFT refinement and re-ranking. Re-optimizing these structures using periodic PBE + D3 in the VASP package (see SI) gave a new, more accurate ordering of the structures, and also allowed for minor structural adjustments that could potentially improve agreement with the residual PXRD signal.

**Fig. 3:**
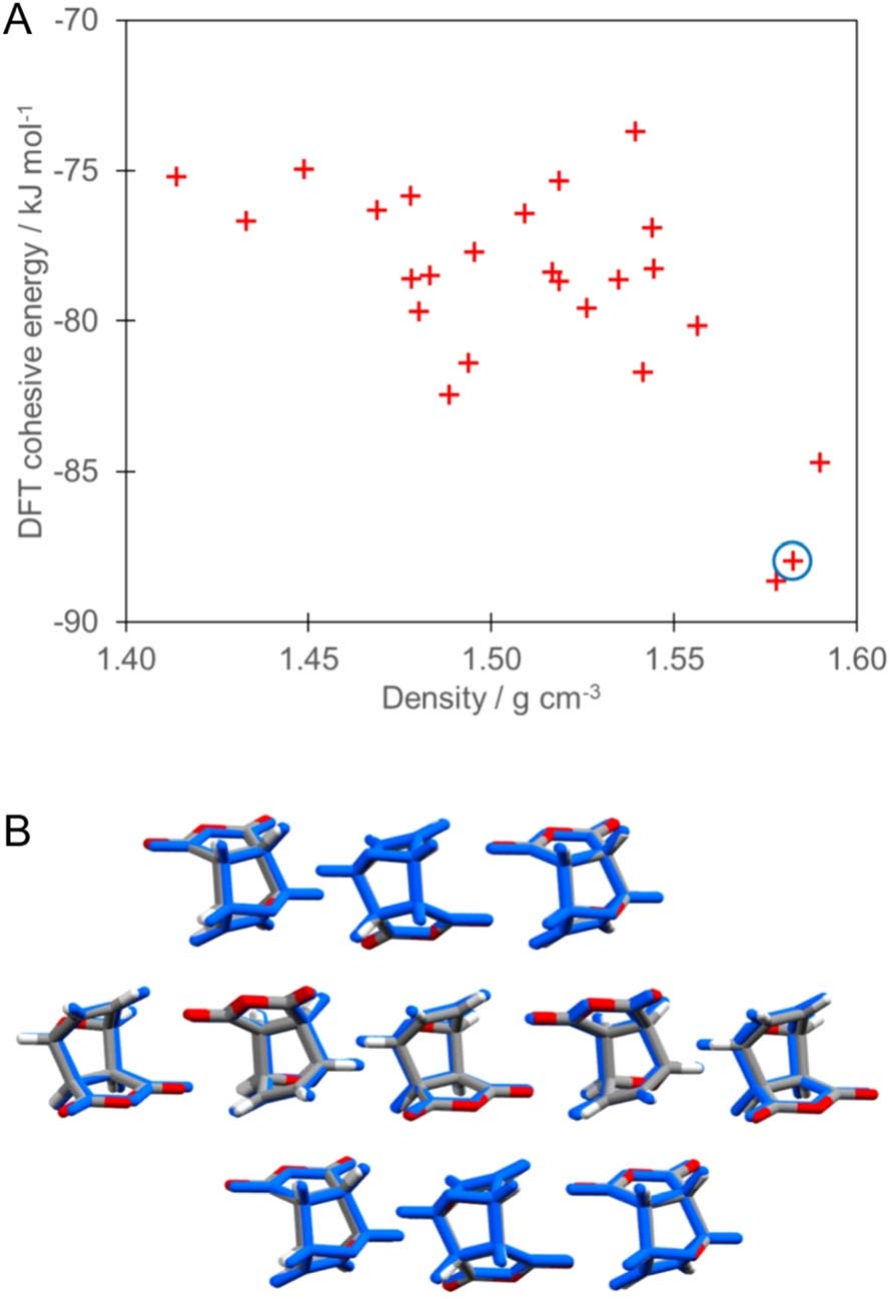
(A) The reoptimised and re-ranked CSP landscape for the *endo*-1 form, now at the periodic DFT (PBE + D3) level of theory. Only points considered consistently reasonable matches to the residual PXRD pattern (see Fig. 2A) were carried forward for refinement, hence only red crosses in this figure. The blue circled structure, the now-refined version of the same structure highlighted in Fig. 2, displayed the overall best match to the residual pattern and was thus assumed to represent the most likely structure of the *endo*-1 form, subsequently confirmed experimentally. (B) An overlay of the single-crystal X-ray diffraction determination of the pure form of *endo*-1 (coloured by element) and the proposed structure from CSP (blue). The RMSD (ignoring hydrogen atoms) of atomic coordinates between 30-molecule clusters from the two structures is 0.183 Å.

Performing the same PXRD comparison now using these DFT-optimised structures revealed a single structure that is the closest match to the residual pattern across the whole range of values of the cDTW constraint considered (pattern shown as the blue line in Fig. 2B). This structure is also the second-lowest energy candidate within this subset of re-ranked structures, and is separated from the minimum by less than 1 kJ mol^−1^, well within the uncertainty of this level of theory (Fig. 3). Based on the very competitive energetic ranking and excellent agreement with the residual PXRD pattern, we anticipated that this predicted crystal structure should correspond to the experimentally observed pure *endo*-1 form.

This anticipation was ultimately validated when we were able to isolate crystals with a new set of unit cell parameters, distinct from those of *exo*-1 or (*endo*-1)(*exo*-1). Diffraction-quality crystals of this phase were first obtained by sublimation of the solidified reaction mixture onto a cold finger, but were subsequently also found to be isolatable directly, by careful analysis and picking from the solidified reaction mixture. Crystal structure determination by single crystal X-ray diffraction revealed that the crystals do indeed correspond to pure *endo*-1 (Fig. 1D), representing the very first structural analysis of this molecule as a pure single-component solid. The experimentally determined structure was found to be an excellent geometric match to the crystal structure of *endo*-1 anticipated from the CSP protocol (Fig. 3B). The root-mean-squared deviation (RMSD) in atomic positions of 30-molecule clusters taken from the CSP structure and the single-crystal solution (overlaid in Mercury^41^) is 0.183 Å, comparable to the typical deviations introduced by thermal fluctuations in crystal structures solved at cryogenic *vs.* room temperature (*cf.* 0.160 Å deviation for paracetamol I at 20 K *vs.* 330 K).^55^

**Fig. 4:**
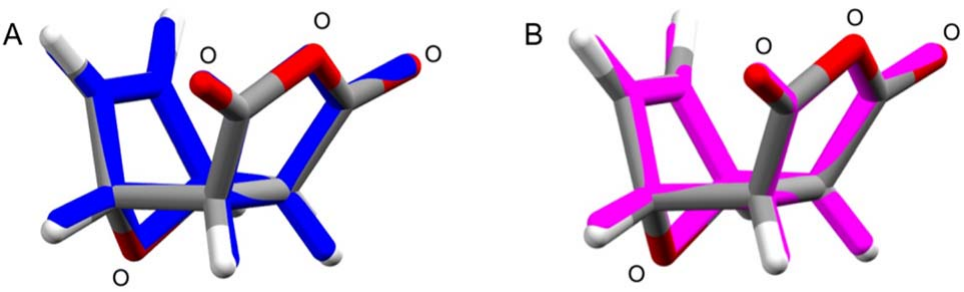
Molecular overlays of the DFT gas-phase-optimized conformer of *endo*-1 (standard element colors) with its experimental in-crystal conformations of the molecule in both (A) the pure form, blue, RMSD = 0.034 Å) and (B) the (*endo*-1)(*exo*-1) cocrystal (magenta, RMSD = 0.037 Å). Oxygen atoms are labelled for clarity.

The molecular geometry of *endo*-1 does not noticeably change between pure *endo*-1 and the (*endo*-1)(*exo*-1) cocrystal (Fig. 4). The conformations of *endo*-1 molecules in both solid forms exhibit a RMSD in atomic positions of approximately 0.3 Å (including hydrogen atoms) compared to the gas-phase conformer, and differ mutually by less than 0.2 Å in RMSD.

DFT optimisation was performed for *exo*-1, giving a total energy 9.5 kJ mol^−1^ lower than the newly-discovered *endo*-1 structure. Partitioning the DFT total energies (see SI for details) shows that *exo*-1 is favoured by intermolecular interactions in the crystal, as well as the greater intramolecular stability: the molecular energy is 6.4 kJ mol^−1^ more stable in *exo*-1 than *endo*-1 and intermolecular interactions are 3.1 kJ mol^−1^ more stabilising in *exo*-1 than *endo*-1. These results are consistent with relative molecular energies calculated by Alves and Fernández^26^ and support the conclusion that *endo*-1 is thermodynamically disfavoured and is observed here because crystallisation from a neat reaction mixture occurs more quickly than full conversion to *exo*-1.

**Fig. 5:**
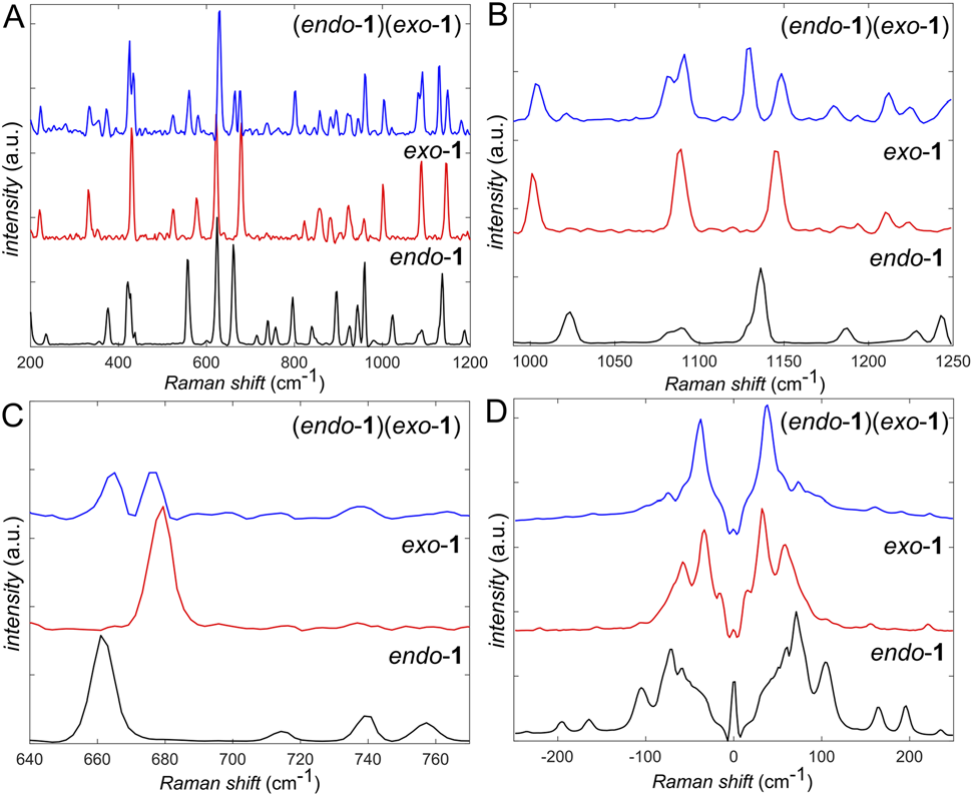
Characterization of single crystals of *endo*-1 (black), *exo*-1 (red) and (*endo*-1)(*exo*-1) (blue) using high- and low-frequency Raman spectroscopy in the: (A) full 200–1200 cm^−1^ range, (B) 1000–1250 cm^−1^ range, (C) 500–780 cm^−1^ range, and (D) low-frequency Raman region (below 200 cm^−1^). Spectra were assigned by comparison to reference spectra measured on crystals previously identified by single crystal X-ray diffraction.

While searching for crystals of *endo*-1 and (*exo*-1)(*endo*-1) was initially based on picking individual crystals from solidified polycrystalline samples, followed by mounting them on an X-ray single crystal diffractometer head and determining unit cell parameters, it was subsequently established that the three phases *exo*-1, *endo*-1 and (*exo*-1)(*endo*-1) could be readily distinguished by fingerprint- and low-frequency Raman spectroscopy.^56^ This observation provided a simpler methodology to identify single crystal samples of each material. Crystals of *exo*-1 and *endo*-1 are readily distinguished by differential characteristic bands in the 1150 cm^−1^ and the 670 cm^−1^ regions, whereas the Raman spectrum of (*endo*-1)(*exo*-1) exhibits features that resemble those of both *exo*-1 and *endo*-1, which is consistent with that spectral region corresponding largely to intramolecular vibrations. In the low-frequency terahertz region, the three phases can be characterised by unique signals: 71, 104 and 163 cm^−1^ for *endo*-1, 34 and 59 cm^−1^ for *exo*-1, and 38 and 159 cm^−1^ for (*endo*-1)(*exo*-1) (Fig. 5D).

### Discovery of the furan solvate of *exo*-1

In the course of searching for a single crystal of *endo*-1 from the reaction vessel, a small portion of picked crystals were found to rapidly become milky upon handling in air. By rapid handling and low-temperature X-ray diffraction, it was eventually possible to collect X-ray diffraction data on one such crystal, revealing that it was a solvate of *exo*-1 with furan, of composition (*exo*-1)(furan). The structure was found to consist of furan molecules, disordered equally over two overlapping positions, occupying channels formed by *exo*-1 that propagate along the crystallographic *b*-axis.

**Fig. 6:**
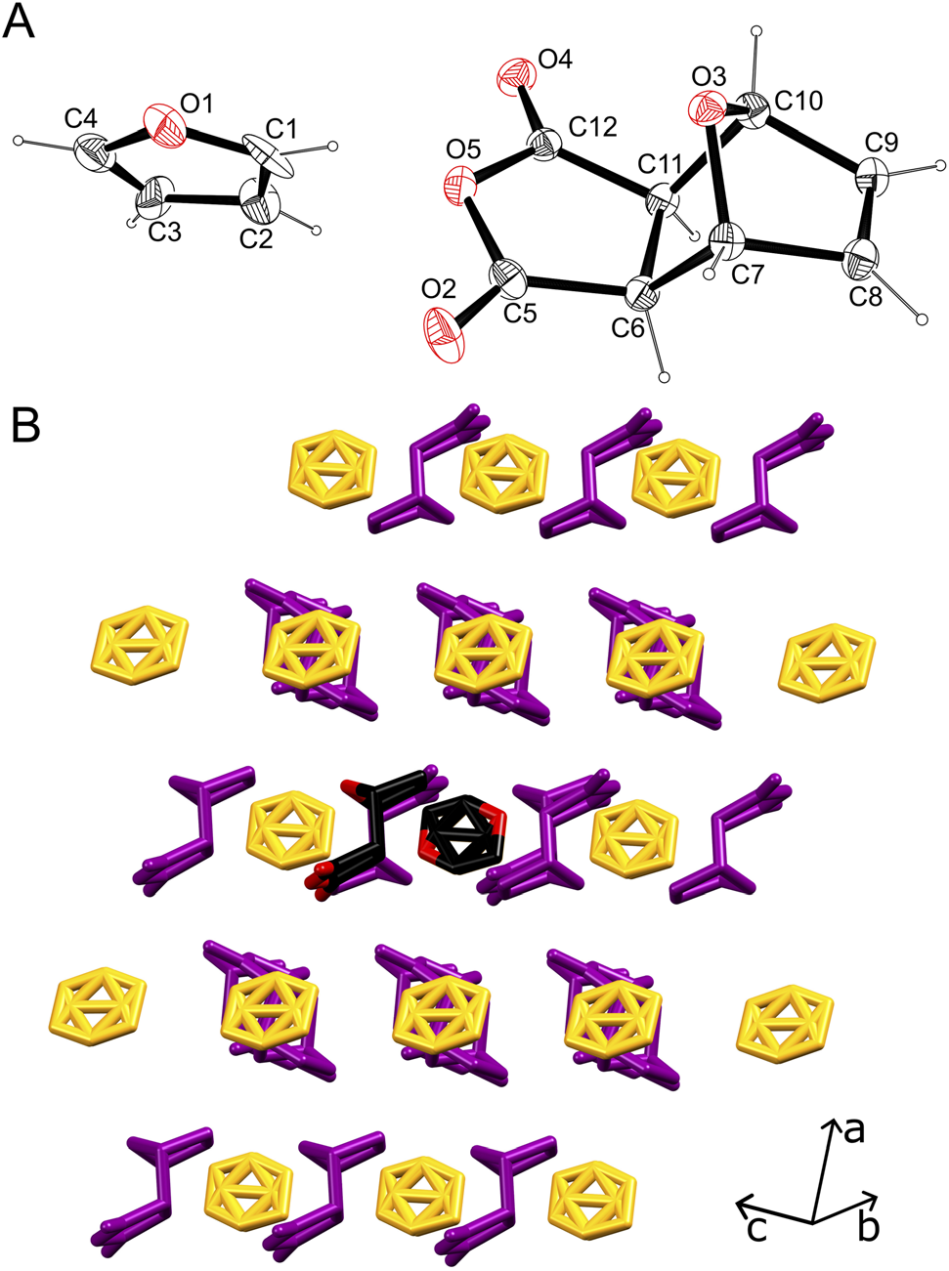
View of the crystal structure of (*exo*-1)(furan): (A) ORTEP view of the asymmetric unit with non-hydrogen atoms shown as ellipsoids corresponding to 30% probability of electron density and (B) molecular arrangement viewed parallel to crystallographic 011-direction, with *exo*-1 molecules shown in purple and disordered furan molecules in yellow. One disordered molecule of furan and one molecule of *exo*-1 are shown in element colors. Hydrogen atoms have been omitted for clarity.

The channel solvate structure suggests that the rapid loss of crystallinity is likely due to the loss of furan (Fig. 6). Indeed, in one example, a sample of the reaction mixture was allowed to remain open to air for 24 hours, leading to apparent decomposition of a number of crystals. For that sample, ^1^H NMR analysis showed a loss of *ca.* 2% of furan without a change in the relative ratio of *endo*-1 to *exo*-1. The discovery of the (*exo*-1)(furan) phase is notable considering that, to the best of our knowledge, the only solid phase known and characterised in the maleic anhydride-furan system since 1920s has been the crystalline *exo*-1.

### Calculating thermodynamic stability of (*endo*-1)(*exo*-1)

With the crystal structures accessible, we were able to determine the relative thermodynamic stability of the cocrystal and the individual components, using accurate lattice energies calculated for the *endo*-1 and *exo*-1 crystals, and for the (*endo*-1)(*exo*-1) cocrystal. Initially, we applied the same periodic DFT approach that we had previously used to re-rank the CSP landscape of the hypothetical *endo*-1 structures. By using the same method to compute the energies of the optimised crystal structures, we determined the static cohesive energies for the three systems. While these energy values account for any differences in molecular conformation between the systems, they lack any description of vibrational or thermal effects. Nonetheless, this approximation was previously shown to reliably predict experimentally observed cocrystals, which were consistently calculated as more stable compared to corresponding mixtures of pure solid components.^36^ In this case, however, the zero-temperature lattice energy description predicts a very slight positive energy difference (+0.7 kJ mol^−1^) between the (*endo*-1)(*exo*-1) cocrystal and a 1 : 1 physical mixture of single-component *exo*-1 and *endo*-1 crystalline solids systems. This suggests that the cocrystal should be slightly disfavoured and, given the small magnitude of this difference compared with the uncertainty in the calculated energies, suggests no energetic motivation for cocrystal formation.

To determine whether the lack of vibrational contributions could be the cause of the inconclusive energetic results, we sought to refine the calculation of crystal energies by incorporating a description of these effects that would be less costly than full periodic DFT phonon calculations. Previous work^57^ has established that phonons computed using anisotropic, atom–atom force field methods can be competitive with those computed using DFT, capable of accurately evaluating the vibrational contributions to the crystalline free energy.^58^ We therefore chose to compute the vibrational contributions at the same multipole-and-force-field level as was used during the initial CSP structure optimization, calculated using the AutoLD/AutoFree utilities of Nyman *et al.*^52^ This method evaluates phonon frequencies across the Brillouin zone using supercells of the provided structure, combined with a Debye approximation for the low-frequency, acoustic phonons and a kernel density estimator (KDE) to model the broadening of the optical phonon density of states (more detail in SI).^37,52^ By combining the results of the periodic DFT static energy calculations with the force-field-level vibrational free energy contributions, we approximate the free energies for all three systems.

**Fig. 7:**
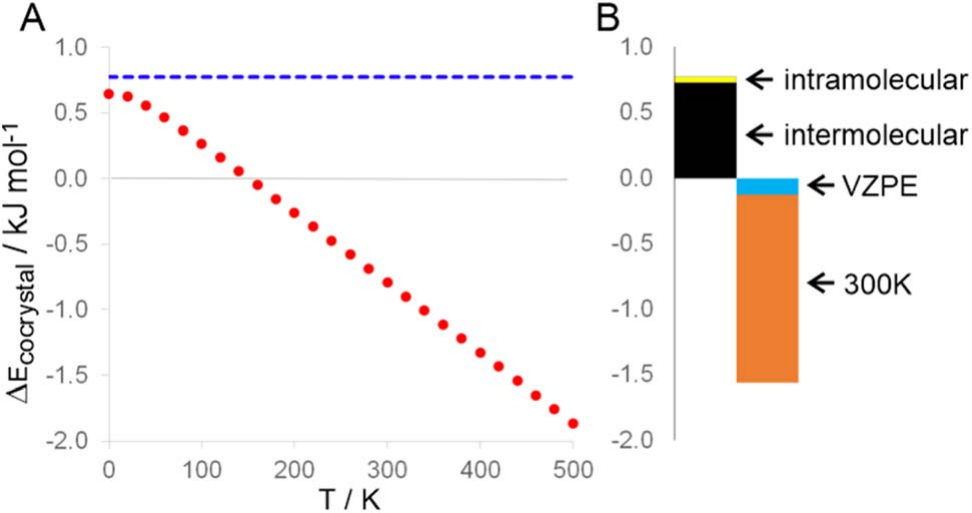
(A) The relative stability of the (*endo*-1)(*exo*-1) cocrystal compared to the 1 : 1 sum of pure form (*endo*-1 + *exo*-1) energies as a function of temperature. The static, zero-temperature lattice energies (blue line) indicate a slight energetic disfavouring of the cocrystal, while the temperature-dependent free energy calculations (red squares) show a qualitative change in the relative stability, with increasing temperature favouring the cocrystal. (B) A bar chart of the separate static and vibrational contributions to the relative free energy of cocrystallisation at 300 K, using the same vertical energy axis as (A). Both the static lattice (black) and intramolecular strain (yellow) energy terms – the only terms present in the static calculations – disfavour the cocrystal, while the zero-point energy (blue) and the thermal contributions (orange) obtained from the dynamical calculations are stabilizing (and dominated by the latter at higher temperatures).

Taking vibrational effects into account led to a marked improvement in the agreement with experimental data. The cocrystal is still predicted to be higher in free energy than a 1 : 1 mixture of single-component crystals at low temperatures, but becomes increasingly lower with increasing temperature and the cocrystal is calculated to be the stable form at room temperature (Fig. 7). While the magnitude of the energies involved is small compared to the uncertainty and the predicted transition temperature is thus not expected to be quantitatively accurate, a clear trend is observed in behaviour with respect to temperature that agrees qualitatively with experimental observations. At low temperatures, the calculations suggest that the single-component forms are more stable, and thus form in preference when the reaction is performed at low temperature. However, as temperature increases, our calculations propose that vibrational effects preferentially stabilise the cocrystal, rationalising the presence of the cocrystal in the reaction product mixture at room temperature.‡‡A small error in the relative static DFT energies has a large impact on the predicted transition temperature. A 1 kJ mol^−1^ change in the static energy difference introduces a change of *ca.* 190 K in the transition temperature. Depending on the accuracy of the predicted transition temperature, the calculated stabilisation of the cocrystal compared to separate *endo*-1 and *exo*-1 solids might support a kinetic rationalisation of the observed temperature-dependent crystallisation behavior in a way akin to Ostwald's rule of stages, with crystallisation at potentially higher supersaturation at a lower temperature might lead to a metastable product.^60^ The intramolecular strain contribution to energies is insignificant, confirming the assumed rigidity of these molecules' solid-state conformations.

With the caveat that the preferred formation of different solid forms in experiments conducted at different temperatures might also be the result of kinetic phenomena^59,60^,‡ (including relative rates of nucleation, crystal growth, competition with other solid forms such as the furan solvate of *exo*-1, *etc*), we note that thermally-dependent stabilisation of a cocrystal of isomers over a mixture of individual pure isomer solids is reminescent of the behavior sometimes seen for other types of isomers, notably enantiomers.^61^ A famous example is the historical work of Pasteur on chirality of tartrate salts where, notwithstanding the differences in level of hydration of racemic and enantiopure phases, temperature was key in enabling the manual separation of a conglomerate mixture of enantiopure solids, as opposed to a crystalline racemate.^62^

### Thermal analysis and hot-stage microscopy

**Fig. 8:**
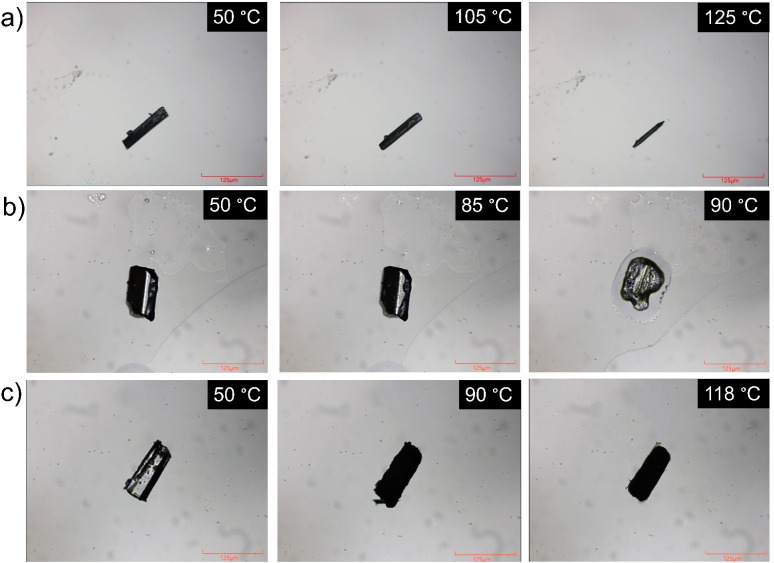
Selected thermal microscopy images of a crystal of (a) *exo*-1; (b) *endo*-1, and (c) the cocrystal (*exo*-1)(*endo*-1). More images and videos of thermal microscopy experiments are provided in the SI and supplementary Movies S1–S5.

Although *endo*-1 and (*exo*-1)(*endo*-1) were available only in the form of hand-picked individual crystals, these were suitable for a qualitative investigation of their solid-state thermal properties using open sample hot-stage microscopy and variable temperature single crystal X-ray diffraction. The behavior of these two crystals under open-air hot-stage microscopy was considerably different from that of a crystal of *exo*-1, which was found to slowly reduce in size during heating due to evaporation (Fig. 8A).^63^ Nevertheless, melting of *exo*-1 could be observed at *ca.* 119 °C if hot-stage microscopy was conducted on a covered, polycrystalline sample. Specifically, a crystal of *endo*-1 was found to cleanly melt at *ca.* 90 °C to form a clear liquid (Fig. 8B). Thermal microscopy behavior of (*exo*-1)(*endo*-1) is distinct from that of either cocrystal constituent, as the cocrystal was found to darken around 90 °C and then continued to evaporate without visible melting (Fig. 8C). Low-frequency Raman spectroscopy analysis of the initially (*exo*-1)(*endo*-1) cocrystal after being held at 100 °C indicates that the darkening of the crystal is associated with transformation to solid *exo*-1 (see SI). We note that the behaviour of both the (*exo*-1)(*endo*-1) cocrystal and phase-pure crystalline *endo*-1 bears some resemblance to those ascribed to solid *endo*-1 by Eggelte.^29^ The hot-stage microscopy measurements on selected crystals of *endo*-1, (*exo*-1)(*endo*-1) and *exo*-1 are also provided as SI Movies S1–S5. The crystallographic analysis of the single crystals of (*exo*-1)(*endo*-1) and *endo*-1 at different temperatures also enabled the characterization of their solid-state thermal behaviour, notably the determination of thermal expansion coefficients^64–67^ which are comparable (between 30 and 80 MK^−1^) to those calculated for *exo*-1 based on previously reported crystal structure determinations (see SI).

## Conclusion

In summary, investigating the neat reaction of furan and maleic anhydride led to the long-missing structural characterization and discovery of multiple solid forms of the elusive Diels–Alder *endo*-adduct. We note the importance of solventless reaction design for enabling the crystallization of solid forms of the *endo*-adduct before the usual complete reversal to the *exo*-isomer. More broadly, this observation reflects a general ability of solventless reactions and crystallization pathways to sometimes access and stabilise crystallographically well-defined molecular solid forms that are not readily accessible through conventional solution crystallization, such as unusual tautomers,^68^ kinetically persistent crystalline phases,^69^ or molecular species that rapidly dissociate in dilute solutions.^70^ Analysis of the solidified Diels–Alder reaction mixture through a combination of X-ray diffraction and CSP led to three additional crystalline phases in a well-known and highly-studied reaction system, for which the only structurally characterised phase so far has been the *exo*-adduct. The use of CSP to characterise the crystal structure of the pure *endo*-1 form was facilitated by the availability of experimental PXRD data containing signals of this solid form, which allowed for the winnowing of an otherwise highly varied landscape of putative crystal structures down to a tractable number of candidates that showed good agreement with the experiment, and the subsequently obtained crystal structure. This work demonstrates the complementary power of combining experiment and theory, in which the provision of even partial or incomplete experimental data, for example the residual PXRD pattern presumed to signify the *endo*-1 form, enabled selective deployment of more accurate computational methods that would be intractably expensive to apply to the CSP landscape based on energy rankings alone. These more advanced calculations on structures that align with the experimental data can then, in turn, more reliably propose solutions to be verified by further experiments. The validated demonstration of structure identification from laboratory PXRD data has wider relevance in materials discovery, particularly with significant recent development of automation in materials synthesis and characterization.^71,72^ Predicted structural landscapes, along with algorithms for structure matching, could support the workflows for self-driving labs by supplying feedback on the full crystal structure, predicted properties and the presence or absence of alternative low energy polymorphs. Furthermore, the observed temperature sensitivity of (co)crystallisation demonstrates the complexities of even well-studied experimental systems, as well as the need for continued development and validation of sophisticated computational methods for free-energy calculations of solids. Indeed, the free energy calculations for the cocrystal and physical mixture of pure isomer solids can now suggest an explanation for previously reported reproducibility issues in obtaining crystalline *endo*-1. Finally, the observation of a furan solvate of the *exo*-isomer is also notable not only because of the previously not recognised, potentially limiting role that such a cocrystal would have on the progress of the solventless reaction, but also because it adds to the unexpected diversity of phases relevant to this well-known reaction.

Overall, the herein presented work advances the understanding of a historically-significant Diels–Alder reaction that has been attracting attention for almost a century, highlights the value of high-end theoretical modelling for understanding complex solid-state reaction mixtures, and demonstrates the benefits of exploring solventless and neat reaction environments, even for systems that have been extensively studied. We are currently working towards developing a further understanding of the stability, and conditions for preferential formation, of solid forms reported here.

## Author contributions

The manuscript was written through contributions of all authors, and all authors have given approval to the final version of the manuscript. Conceptualization: TF, GMD, CBL, IH; data curation: TF, GMD; formal analysis: TF, GMD, CBL, CRT, IH, RSS, JV, THB, ARBJLG; funding acquisition: TF, GMD; investigation: all authors; methodology: TF, GMD, IH, CBL, THB; project administration: TF, GMD; resources: TF, GMD; software: GMD; supervision: TF, GMD; validation: TF, GMD, CBL, IH, CWN, JMM; visualization: TF, GMD, CBL, CRT, THB.

## Conflicts of interest

There are no conflicts to declare.

## Supplementary Material

SC-OLF-D5SC06724C-s001

SC-OLF-D5SC06724C-s002

SC-OLF-D5SC06724C-s003

SC-OLF-D5SC06724C-s004

SC-OLF-D5SC06724C-s005

SC-OLF-D5SC06724C-s006

SC-OLF-D5SC06724C-s007

## Data Availability

Data for this article are available at Zenodo at DOI: 10.5281/zenodo.20852658. CCDC 2424954, 2424955, 2376885–2376894 contain the supplementary crystallographic data for this paper.^73*a*–*l*^ Supplementary information (SI): experimental and theoretical procedures, along with results of powder and single crystal X-ray diffraction, NMR spectroscopy, hot-stage microscopy and analysis of thermal expansion behavior (PDF), as well as crystal structure data, including CheckCIF information (CIF, PDF), and movies of hot-stage microscopy experiments (MP4). See DOI: https://doi.org/10.1039/d5sc06724c.
