# Supplementary material for: The elusive endo-product of the archetypal Diels–Alder reaction of furan and maleic anhydride – observed in the solid state at last
Source: Chem Sci. 2026 May 13. Online ahead of print. doi: 10.1039/d5sc06724c (PMC13321552; doi:10.1039/d5sc06724c)
Supplement: SC-OLF-D5SC06724C-s006 [file SC-OLF-D5SC06724C-s006.pdf]

Supplementary Information (SI) for Chemical Science.
This journal is © The Royal Society of Chemistry 2026

Electronic Supporting Information for: The elusive *endo*-product of the archetypal Diels-Alder reaction between furan and maleic anhydride – observed in the solid state at last

Cameron B. Lennox,[a,b] Christopher R. Taylor,[c] Igor Huskić,[b] Tristan H. Borchers,[a] Jogirdas Vainauskas,[a,b] Christopher W. Nickels,[b] Robin S. Stein,[b] A. R. Bonnie J. Lutton-Gething,[a] Joseph M. Marrett,[a] Graeme M. Day[c]* and Tomislav Friščić[a,b,]*

Table of Contents

		Page
**S1.**	**Experimental section**	2
S1.1	General information	2
S1.2	Synthesis and material handling	2
S1.3	Single-crystal X-ray diffraction	2
S1.4	Powder X-ray diffraction	2
S1.5	Solution nuclear magnetic resonance spectroscopy	2
S1.6	Thermal microscopy	3
S1.7	Raman spectroscopy	3
S1.8	Thermogravimetric analysis and differential scanning calorimetry (TGA/DSC)	3
**S2.**	**Powder X-ray diffraction (PXRD) patterns**	4
**S3.**	**Rietveld refinement**	10
**S4.**	**Single-crystal X-ray diffraction**	13
S4.1	ORTEP figures	28
S4.2	Principal axis strain resulting from thermal expansion	40
**S5.**	**^{1}H- and ^{13}C- nuclear magnetic resonance spectra**	46
**S6.**	**Thermal microscopy**	57
**S7.**	**Thermal microscopy videos**	61
**S8.**	**Raman spectra**	62
**S9.**	**Thermogravimetric analysis (TGA)/Differential scanning calorimetry (DSC)**	63
**S10.**	**Computational details**	64
**S11.**	**References**	66

S1. Experimental section

S1.1 General information

Maleic anhydride (99 %, Sigma-Aldrich) was used without further purification and furan (>99 %, Sigma-Aldrich) was purified by vacuum distillation prior to use.

S1.2 Synthesis and material handling

Single crystals of *endo*-**1** were extracted from the solidified reaction mixture for the reaction of an equimolar amount of maleic anhydride (30 mmol, 2.94 g) and furan (30 mmol, 2.04 g) conducted at ca. 4 °C.

Single crystals of (*endo*-**1**)(*exo*-**1**) were extracted from the solidified reaction mixture for the reaction of an equimolar amount of maleic anhydride (30 mmol, 2.94 g) and furan (30 mmol, 2.04 g) conducted at room temperature (ca. 20 °C).

Single crystals of *exo*-**1** were extracted from the solidified neat reaction mixture for the reaction of equimolar amounts of maleic anhydride (30 mmol, 2.94 g) and furan (30 mmol, 2.04 g) conducted at 45 °C.

For improved sampling and analysis of the overall composition of reaction mixtures, the reactions were conducted at a smaller, 3 mmol scale.

S1.3 Single-crystal X-ray diffraction

Single crystal X-ray diffraction (scXRD) data for (*exo*-**1**)(*endo*-**1**) and *endo*-**1** were measured on a Bruker D8 Venture X-ray diffractometer equipped with a Photon 200 area detector, and I µS microfocus X-ray source (Bruker AXS, CuK_α source). Measurements were carried out at different temperatures with an Oxford Instrument CryoStream.

Single crystal X-ray diffraction data for (*exo*-**1**)(furan) and (*exo*-**1**)(*endo*-**1**) were measured on a Agilent SuperNova diffractometer equipped with an Atlas detector and an Oxford Cryostream cooling system, using mirror-monochromated CuK_α radiation (λ = 1.54184 Å) from a microfocus source. Measurements were carried out at temperatures of 120 K, 150 K, 200 K and 298 K. Data was collected in a series of ω-scans. The data collection was driven, processed and an absorption correction was applied using CrysAlisPro.[1]

Single crystals were coated with a thin layer of paratone oil before mounting on a diffractometer, and structure solution was carried out using the SHELXTL package. The parameters were refined for all data by full-matrix-least-squares refinement of F^2 using SHELXL.[2] All the non-hydrogen atoms were refined with anisotropic thermal parameters, and the hydrogen atoms were included in the refinement using a riding model, with their coordinates and isotropic thermal parameters tied to those of their parent carbon atoms.

The structures of (*exo*-**1**)(*endo*-**1**) and *endo*-**1** have been determined several times, using different crystals, to evaluate reproducibility of the solid phases.

Crystallographic data in CIF format for all herein determined crystal structures can be accessed using the joint CCDC/FIZ Karlsruhe online deposition service, under the deposition numbers 2424954, 2424955, 2376885-2376894.

S1.4 Powder X-ray diffraction

Powder X-ray diffractograms were collected with a Bruker D2 Phaser powder diffractometer, equipped with a CuK_α (λ = 1.5418 Å) source and Lynxeye detector. Powder diffractograms for another set of experiments were measured on a Panalytical AERIS diffractometer with a CuK_α (λ = 1.5418 Å) source and a PIXcel1D-Medipix3 detector. Diffractograms were collected over the range of 5° to 40° (2θ) with a step size of 0.05°.

S1.5 Solution nuclear magnetic resonance (NMR) spectroscopy
Solution ^{1}H and ^{13}C nuclear magnetic resonance spectra were collected using either a Varian Inova (500 MHz for ^{1}H, 125 MHz for ^{13}C) or Bruker AVIII (400 MHz for ^{1}H, 125 MHz for ^{13}C) NMR spectrometer. Chemical shifts are reported relative to CD_3CN (δ 1.94 ppm for ^{1}H; δ 118.26 ppm for ^{13}C), with the spectra measured within 10 minutes of sample dissolution.

For time-dependent ^{1}H NMR measurements (see Figures S36, S37), a 44 mg sample of a 3 mmol scale reaction mixture of maleic anhydride and furan was taken after 19 hours of standing at room temperature and dissolved in 1 mL CD_3CN prior to analysis.

S1.6 Thermal microscopy
Thermal microscopy was performed with a LINKAM LNP96-S temperature-controlled stage, an Allied Vision Alvium 1800 U-1240c camera mounted on a Leica DM2500 LED microscope using a 10x objective. All videos and pictures in Figures S21-S31 include a scale bar (125 μm) and are white balance corrected. All samples were characterized prior to thermal microscopy analysis also by single-crystal or powder X-diffraction, and Raman spectroscopy.

S1.7 Raman spectroscopy
Spectra for *exo*-**1**, *endo*-**1**, (*endo*-**1**)(*exo*-**1**) were recorded using a Coherent THz-Raman system equipped with a 785 nm cleanline laser and a TR-probe mounted on a Leica DM2500 LED microscope with a 10x objective, at ca. 70 mW power with 15 s integration time, and averaged over 50 accumulations. Measurements were performed on single crystals previously assessed by single crystal X-ray diffraction. The reference spectrum for *exo*-**1** was collected ar 150 K. The spectra were manually background corrected using MATLAB background substraction (msbackadj).

S1.8 Thermogravimetric analysis and differential scanning calorimetry (TGA/DSC)
Tandem thermogravimetric analysis (TGA) and differential scanning calorimetry (DSC) experiments were performed with ca. 10 mg of a sample placed in an open alumina pan mounted on a STD 650 TA instrument. Samples were heated from 25 to 215 °C at a rate of 5 °C/min under dynamic nitrogen atmosphere, with a balance and purge flow rate of 50 mL/min.

S2. Powder X-ray Diffraction (PXRD) patterns

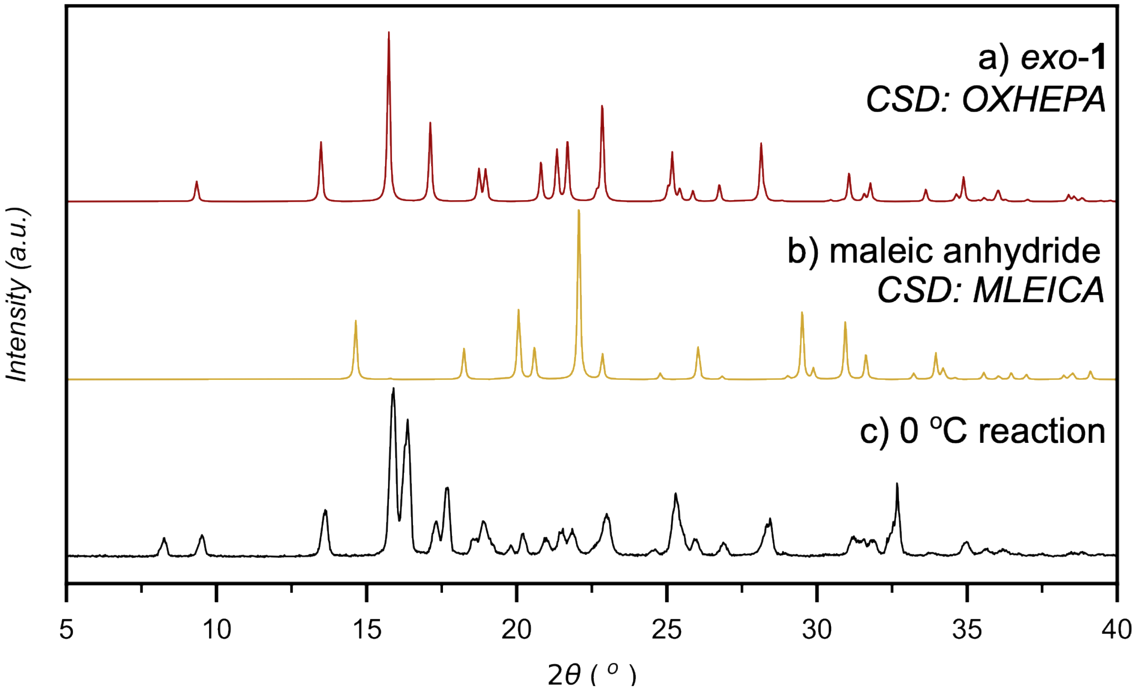

Fig. S1. Powder X-ray diffraction patterns (top-to-bottom): simulated for the single-crystal X-ray structures of *exo*-1 (CSD: OXHEPA) and maleic anhydride (CSD: MLEICA), and measured for the product of the reaction conducted at 0 °C (used for CSP search).

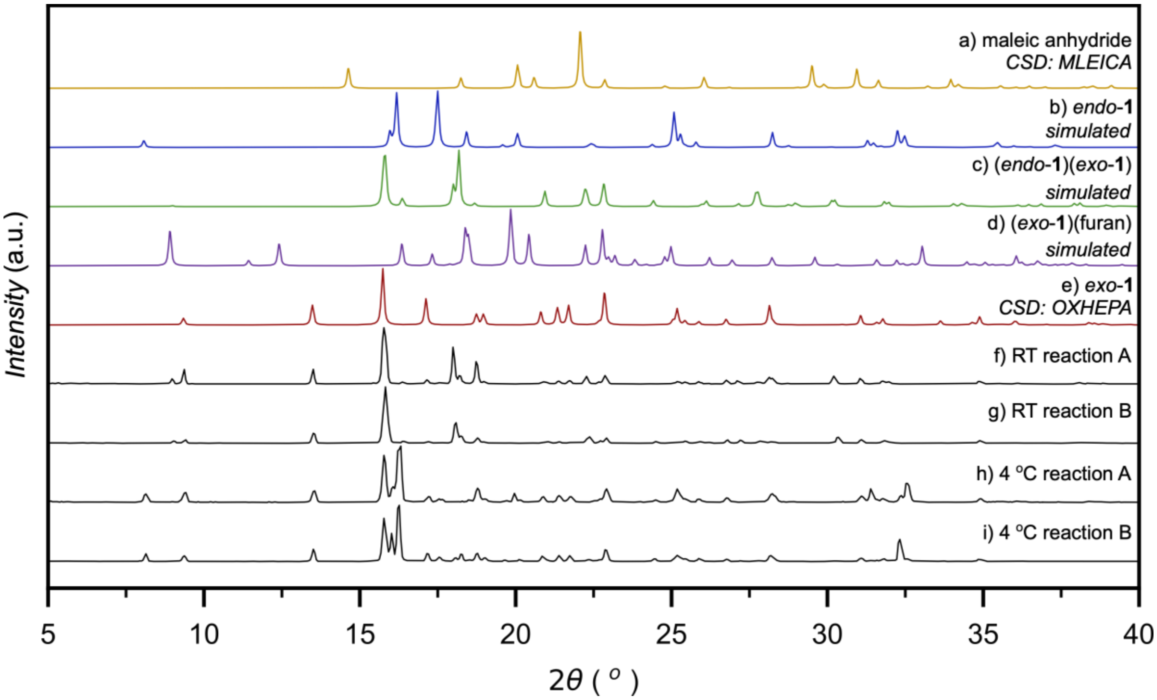

Fig. S2. Selected PXRD patterns for the neat reaction of maleic anhydride and furan (30 mmol scale): a) calculated for maleic anhydride (CSD code: MLEICA), b) calculated for (*endo*-**1**)(furan) collected at 180 K, c) calculated for *endo*-**1** collected at 298 K, d) calculated for (*exo*-**1**)(*endo*-**1**) cocrystal collected at 298 K, e) calculated for *exo*-**1** (CSD code: OXHEPA), f) and g) measured for the product of neat reaction at room temperature conducted in duplicate, displaying Bragg reflections corresponding to the (*endo*-1)(*exo*-1) cocrystal, h) and i) measured for the product of the reaction at 4 °C conducted in duplicate, displaying Bragg reflections indicating a mixture of *exo*-**1** and *endo*-**1**. The PXRD patterns are likely to exhibit preferred orientation, as the samples were not mechanically ground before measurement.

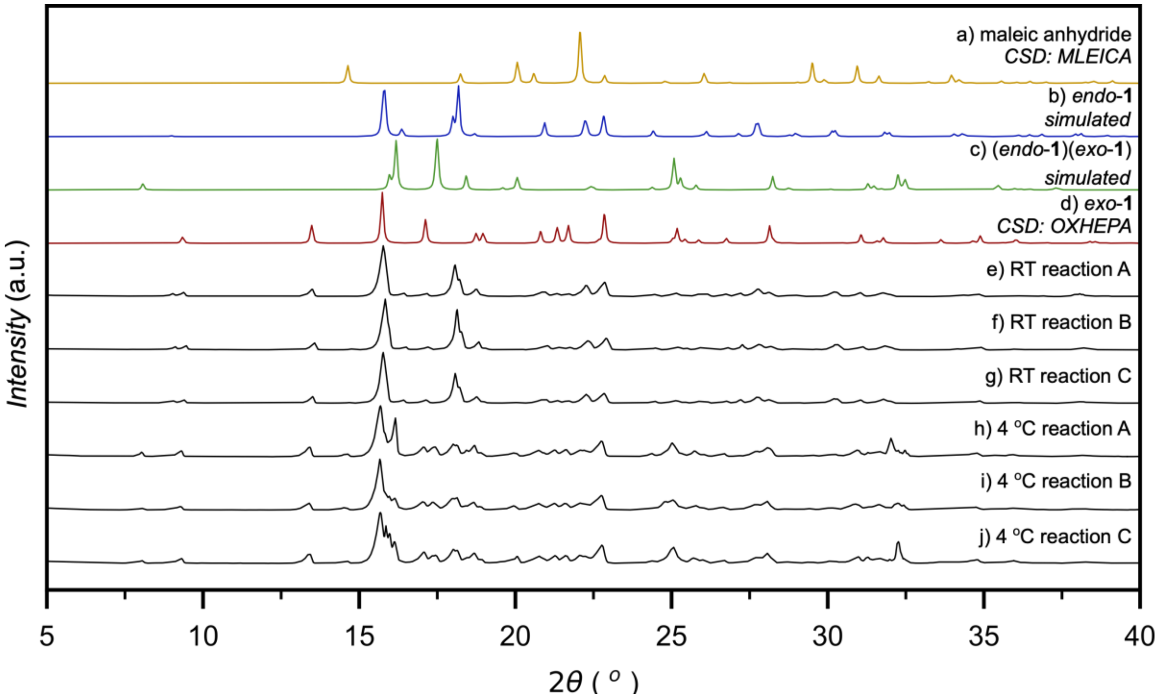

Fig. S3. Selected PXRD patterns for the neat reaction of maleic anhydride and furan (3 mmol scale): a) calculated for maleic anhydride (CSD code: MLEICA), b) calculated for *endo*-**1** collected at 298 K, c) calculated for (*exo*-**1**)(*endo*-**1**) cocrystal collected at 298 K, d) calculated for *exo*-**1** (CSD code: OXHEPA), e-g) measured for the product of the neat reaction at room temperature, in triplicate, displaying Bragg reflections corresponding to the (*endo*-**1**)(*exo*-**1**) cocrystal, and h-i) measured for the product of the reaction at 4 °C, in triplicate, displaying Bragg reflections indicating a mixture of *exo*-**1** and *endo*-**1**.

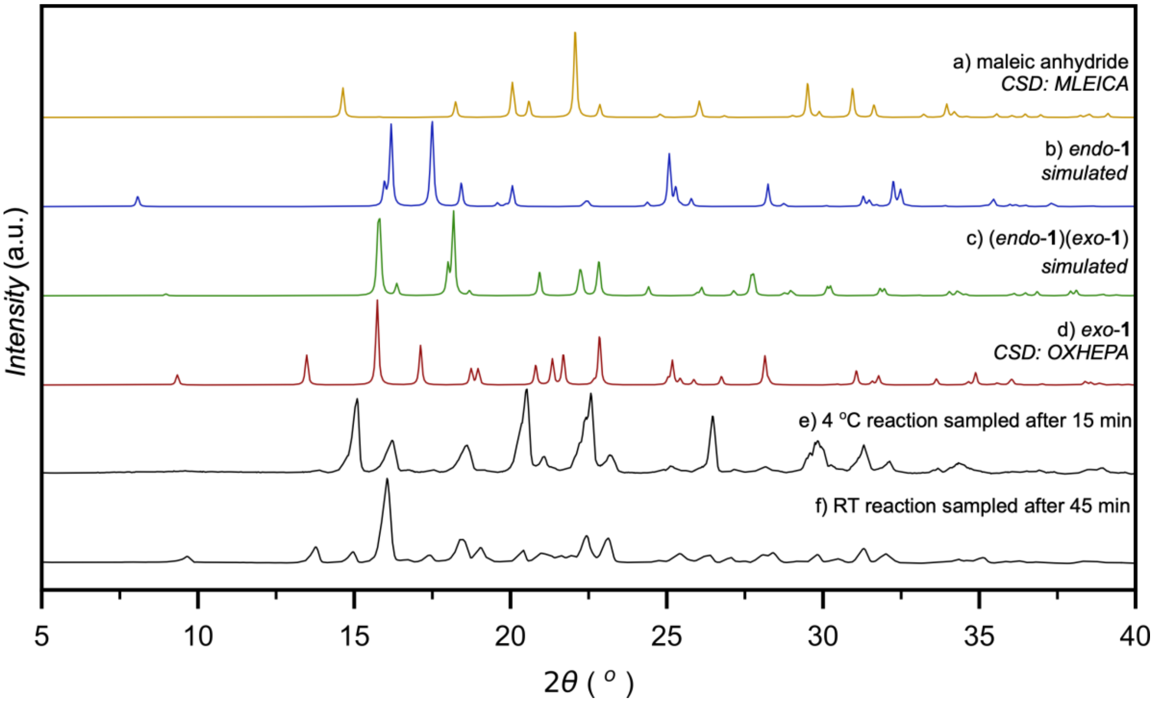

Fig. S4. Selected PXRD patterns for the neat reaction of maleic anhydride and furan: a) calculated for maleic anhydride (CSD code: MLEICA), b) calculated for *endo*-**1** collected at 298 K, c) calculated for (*exo*-**1**)(*endo*-**1**) cocrystal collected at 298 K, d) calculated for *exo*-**1** (CSD code: OXHEPA), e) measured for the crystallized solid product of neat reaction at 30 mmol scale at 4 °C after 15 minutes, displaying Bragg reflections corresponding to predominantly the maleic anhydride, and f) measured for the crystallized solid product of the reaction at 3 mmol scale at room temperature after 45 minutes, displaying Bragg reflections indicating a mixture of *exo*-**1** and maleic anhydride.

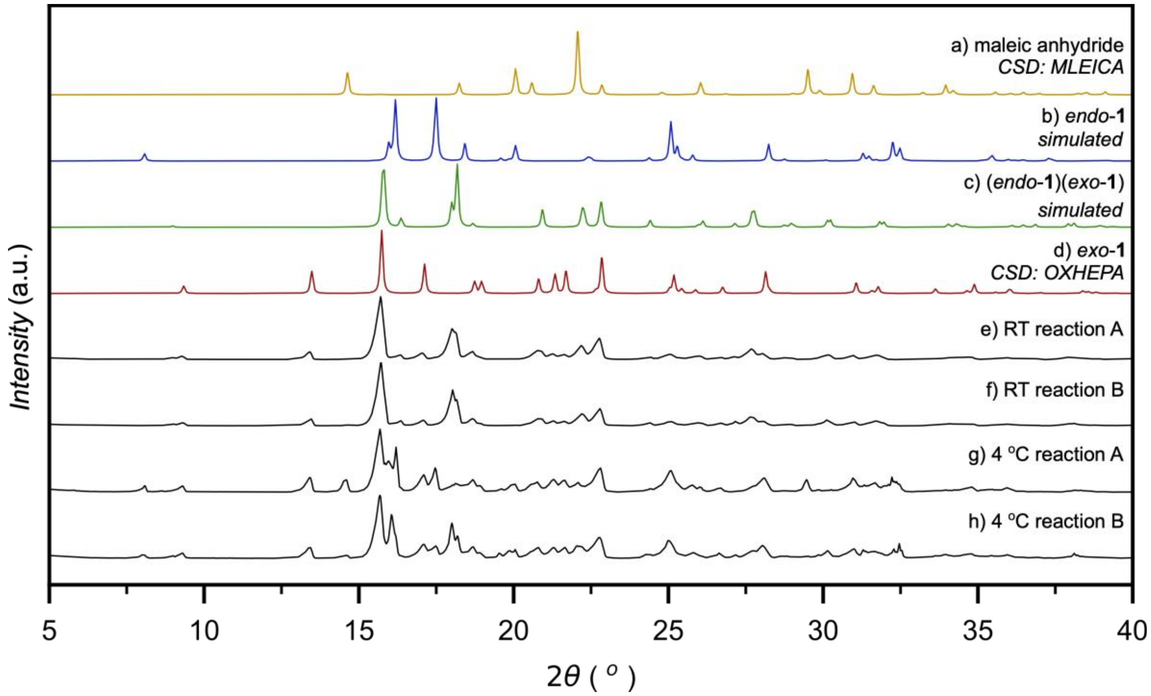

Fig. S5. Selected PXRD patterns for the neat reaction of sublimated maleic anhydride and furan conducted at a 3 mmol scale: a) calculated for maleic anhydride (CSD code: MLEICA), b) calculated for *endo*-**1** collected at 298 K, c) calculated for (*exo*-**1**)(*endo*-**1**) cocrystal collected at 298 K, d) calculated for *exo*-**1** (CSD code: OXHEPA), e) and f) measured for the product of the neat reaction at room temperature, in duplicate, displaying Bragg reflections corresponding to the (*endo*-**1**)(*exo*-**1**) cocrystal, and g) and h) measured for the product of the reaction at 4 °C, in duplicate, displaying Bragg reflections indicating a mixture of *exo*-**1** and *endo*-**1**.

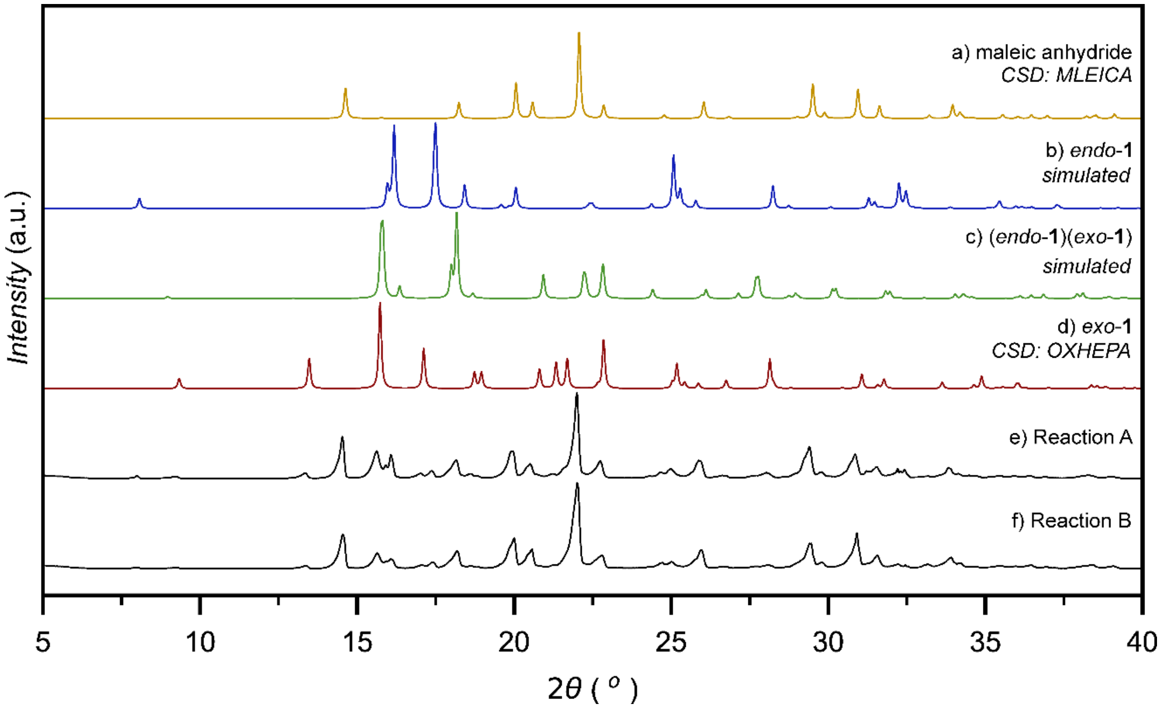

Fig. S6. Selected PXRD patterns for the neat reaction of maleic anhydride and furan: a) calculated for maleic anhydride (CSD code MLEICA), b) calculated for *endo*-**1** structure collected at 298 K, c) calculated for (*exo*-**1**)(*endo*-**1**) cocrystal structure collected at 298 K, d) calculated for *exo*-**1** structure (CSD code OXHEPA), e) and f) measured for the product of the neat reaction conducted at a 3 mmol scale at a temperature between -11 °C and -12 °C over 18 hours, displaying Bragg reflections consistent with the presence of *endo*-**1**.

S3. Rietveld Refinement

Rietveld[3] refinement was attempted for products obtained using sublimated maleic anhydride on a 3 mmol scale at 4 °C and at room temperature (20 °C). XRPD data suitable for refinement was collected with CuK_α (λ = 1.5418 Å) radiation using a Panalytical Aeris diffractometer equipped with an Empyrean Cu LFF HR tube and a PIXcel1D-Medipix3 detector. CuK_β radiation was removed through the use of a 0.02 mm Ni-filter on the diffracted beam. Diffraction patterns were recorded over the 2θ range 5-70°, with a step size of 0.02°. For both the room temperature and low temperature samples a series of 10-minute scans were collected over a total period of 12 hours. This collection strategy was chosen to monitor if any degradation of the sample had occurred over the monitoring period. Due to the high crystallinity of the sample and the quality of the scans only the sum of the final three scans was used for refinements.

For the room temperature phase, no degradation of the sample was observed, and Rietveld refinement was successfully performed over the 2θ-range 7-45° using Topas Academic V7[4] (Figure S6). The background was defined using an 8-component Chebyshev function and the peak shape modelled using an instrument resolution function determined empirically using a THCZ *pseudo*-Voight function against a Si reference pattern collected under equivalent measurement conditions. Domain size and microstrain related broadening effects were accounted for using convoluted Gaussian and Lorentzian functions. Both unit cell parameters and the instrument zero-error were allowed to refine freely and the significant preferred orientation observed was accounted for through the application of an 8th order spherical harmonics correction for each phase. The significant preferred orientation made further refinement of structural parameters challenging, therefore no further optimisation of structural parameters was attempted. Reference single crystal data was collected in-house for *endo*-**1** and (*exo*-**1**)(*endo*-**1**), or acquired from the CSD (*exo-1*: CSD code OXHEPA06; maleic acid: CSD code MALIAC02; maleic anhydride: CSD code MLEICA). The Rietveld refinement results suggest that the room temperature product consist of ~44 wt% *exo*-**1** and ~56 wt%, (*exo*-**1**)(*endo*-**1**), corresponding to relative amounts of *exo*-**1** and *endo*-**1** of ~72 mol% and ~28 mol%, respectively. Notably, whereas ^{1}H NMR indicated the presence of a small amount of maleic anhydride in the sample, this was not evident by PXRD.

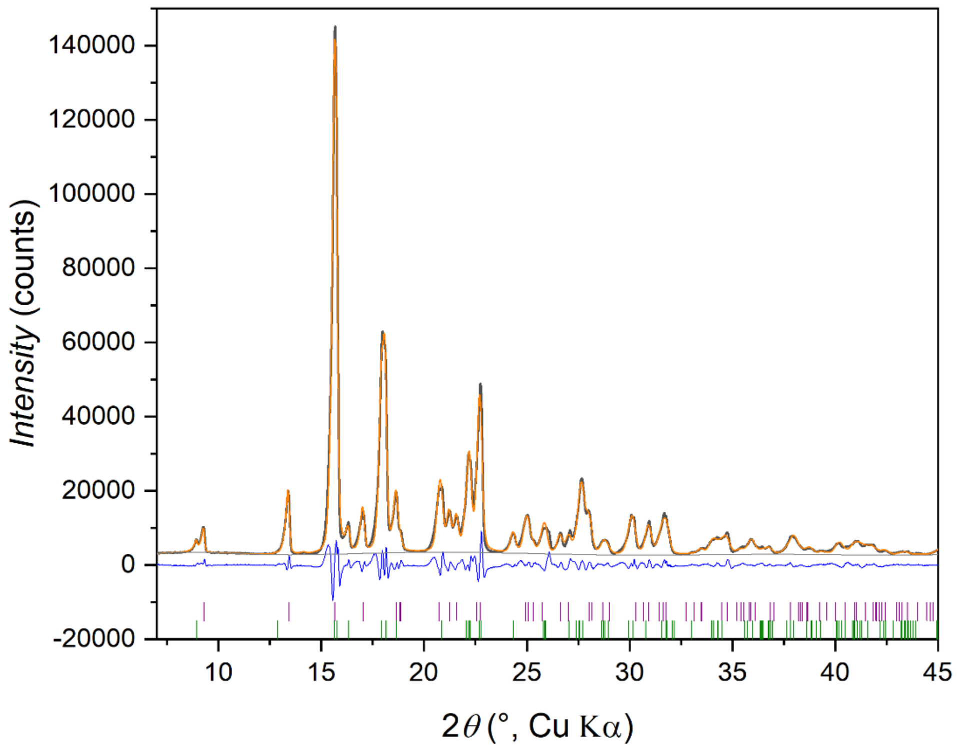

Fig.S7. Final Rietveld refinement result for a sample resulting from room-temperature Diels-Alder reaction of furane and maleic anhydride. Final unit cell parameters for *exo*-**1**: *a* = 11.987(2) Å, *b* = 5.420(1) Å, *c* = 12.074(3) Å, *β* = 110.36(1)°, *V* = 735.5(3) Å^3. R_{wp} 9.10%, R_p 6.62%, R_{exp} = 1.08%. Final unit cell parameters for (*exo*-**1**)(*endo*-**1**): *a* = 11.987(2) Å, *b* = 5.420(1) Å, *c* = 12.074(3) Å, *β* = 110.36(1)°, *V* = 735.5(3) Å^3. R_{wp} 9.10 %, R_p 6.62 %, R_{exp} = 1.08 %. Black = observed, orange = calculated, blue = difference, grey = background, purple = expected *hkl* positions for *exo*-**1** and green = expected *hkl* positions for (*exo*-**1**)(*endo*-**1**).

In contrast to the material produced at room temperature, the material generated at 4 °C was observed to transform over the 12-hour monitoring period, as indicated by the decrease in intensity of Bragg reflections associated with maleic anhydride, within the first 180 minutes of monitoring (see selected scans in Figure S7), and the decrease in intensity of reflections associated with the (*exo*-**1**)(furan). In addition to expected phases, a short-lived, previously unobserved reflection has been identified at 11.1° 2*θ* in scan 1 only. No further significant changes were observed over the remaining 10.5 hours of monitoring. Attempts at Rietveld refinement using data from the scan no. 2 (10 minutes into monitoring), or using the data resulting from summing the last three 10-minute scans of the monitoring experiment were not successful, due to significant preferred orientation in each case.

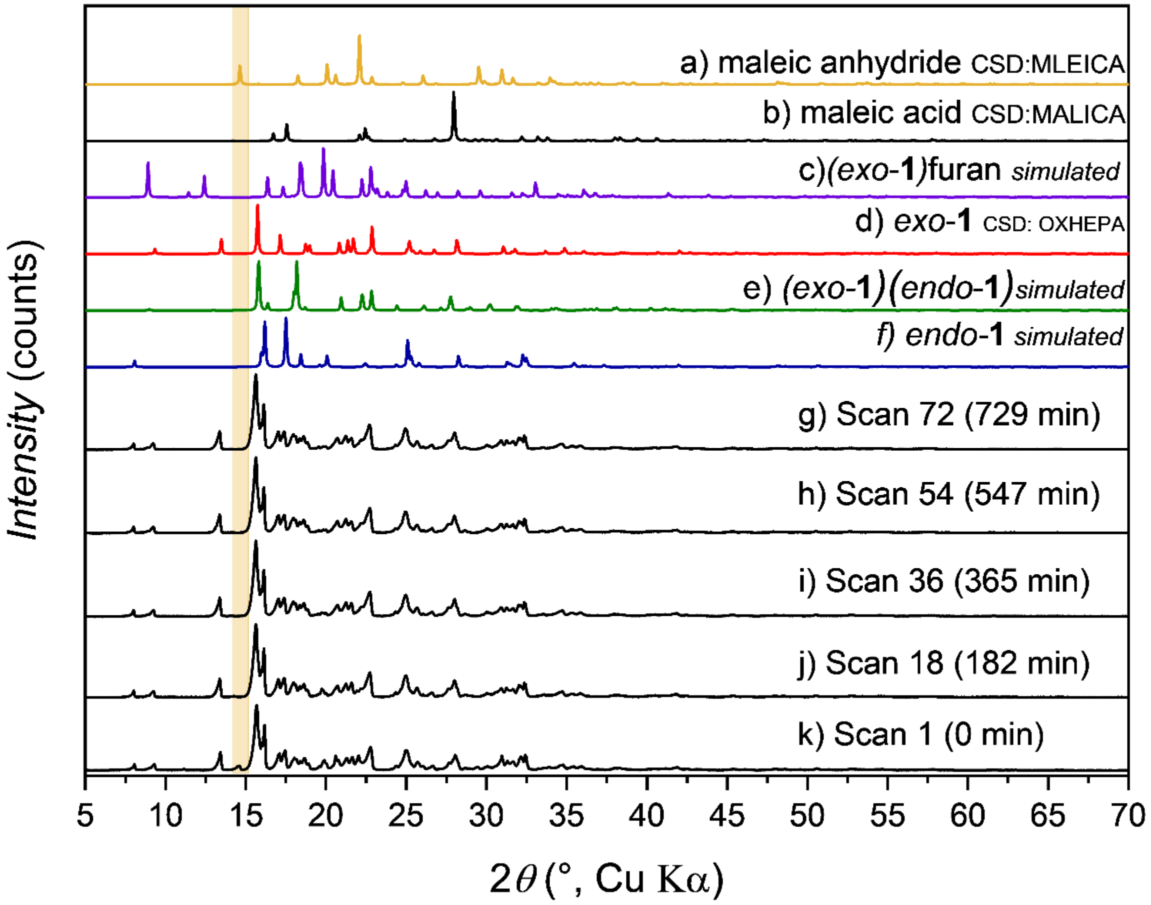

Fig. S8. a) Simulated pattern for maleic anhydride, b) simulated pattern for maleic acid, c) simulated pattern for (*exo*-**1**)furan, d) simulated pattern for *exo*-**1**, e) simulated pattern for (*exo*-**1**)(*endo*-**1**), f) simulated pattern for *endo*-**1**. g) - k) Selected PXRD diffractograms of a 3 mmol scale sample produced at 4°C during time-dependant PXRD monitoring. A loss of Bragg reflections associated with maleic anhydride by 182 minutes might be due to the formation of maleic acid.

S4. Single-crystal X-ray diffraction

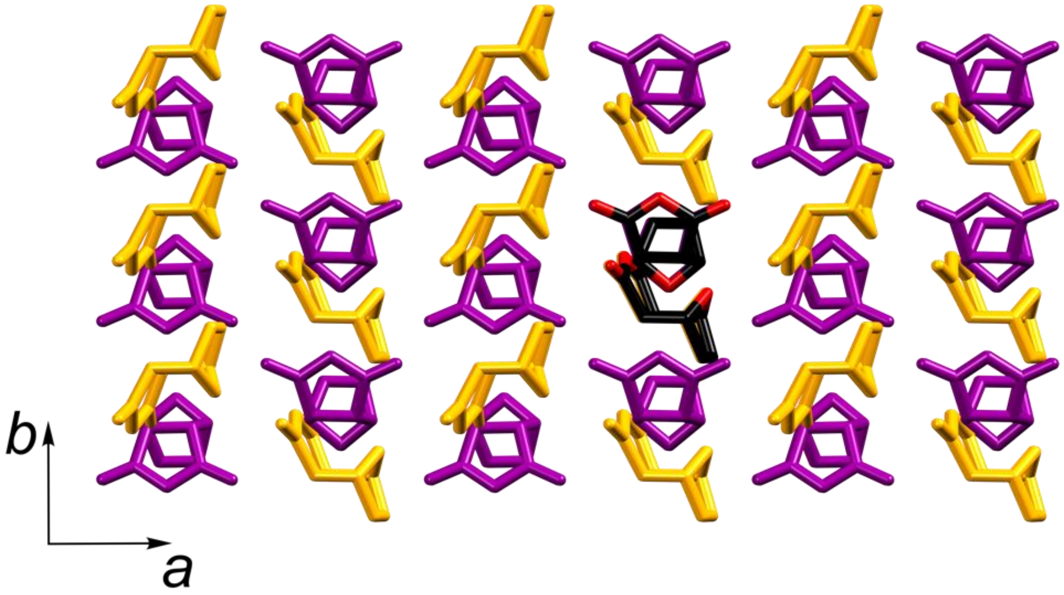

Fig. S9. Arrangement of molecules in solid (*endo*-**1**)(*exo*-**1**) cocrystal at 298 K, shown along the crystallographic *c*-axis. Molecules of *endo*-**1** are shown in purple, and of *exo*-**1** in yellow. For clarity, hydrogen atoms have been omitted and two molecules are shown with element colouring, with carbon atoms in black and oxygen in red.

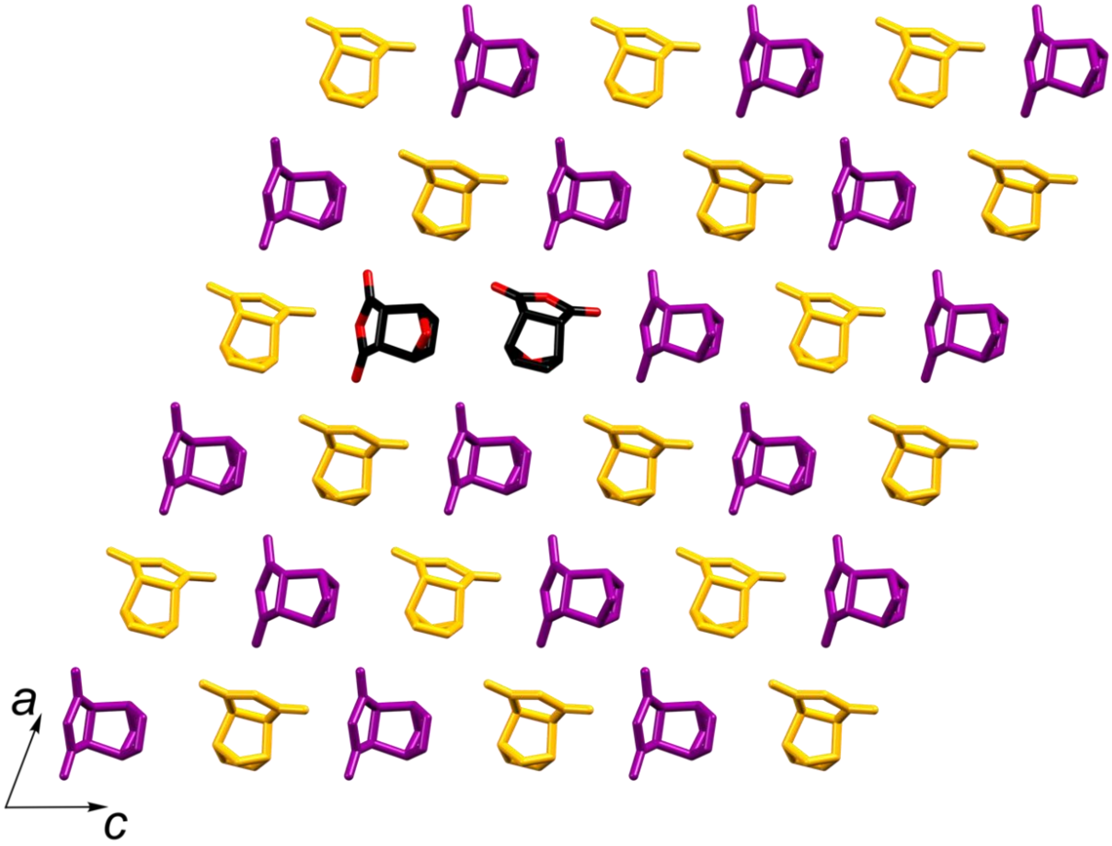

Fig. S10. Arrangement of molecules in solid (*endo*-**1**)(*exo*-**1**), viewed along the crystallographic *b*-axis. Molecules of *endo*-**1** are shown in purple, and of *exo*-**1** in yellow. For clarity, hydrogen atoms have been omitted and two molecules are shown with element colouring, with carbon atoms in black and oxygen in red.

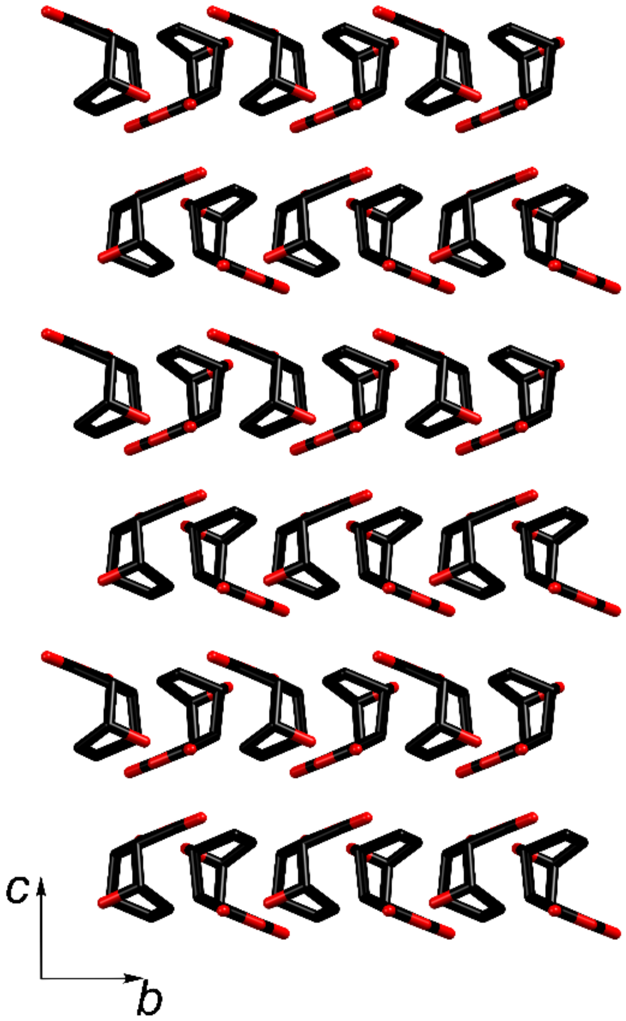

Fig. S11. Arrangement of molecules in solid *endo*-**1**, shown along the crystallographic *a*-axis, with hydrogen atoms omitted for clarity. Carbon atoms are shown in black, and oxygen in red.

Table S1. Crystallographic and general data for a single crystal of (*endo*-**1**)(*exo*-**1**) at 298 K.

CCDC deposition No. : 2376885	
Molecular formula	$C_{16}H_{12}O_8$
M_r (g/mol)	332.26
Crystal system	monoclinic
Crystal colour	colorless
Space group	*Pn*
T (K)	298
a (Å)	11.9547(5)
b (Å)	5.4149(2)
c (Å)	12.0064(5)
α (°)	90
β (°)	110.600(2)
γ (°)	90
V (Å^3)	727.52(5)
Z	2
ρ_{calc} (g/cm^3)	1.517
μ (mm^{-1})	1.066
F(000)	344
Independent reflections	2231 [R_{int} = 0.0660, R_{sigma} = 0.0760]
Data/restraints/parameters	2231/2/217
Goodness-of-fit on F^2	1.148
Final R indices [I>=2σ (I)]	R_1 = 0.0448, wR_2 = 0.1199
Final R indices [all data]	R_1 = 0.0960, wR_2 = 0.1627
Largest diff. peak/hole / e Å^{-3}	0.39/-0.36

Table S2. Crystallographic and general data for a single crystal of (*endo*-**1**)(*exo*-**1**) at 240 K.

CCDC deposition No. : 2376886	
Molecular formula	$C_{16}H_{12}O_8$
M_r (g/mol)	332.26
Crystal system	monoclinic
Crystal colour	colorless
Space group	*Pn*
T (K)	240
a (Å)	11.9288(4)
b (Å)	5.3945(2)
c (Å)	11.9826(5)
α (°)	90
β (°)	110.504(3)
γ (°)	90
V (Å^3)	722.23(5)
Z	2
ρ_{calc} (g/cm^3)	1.528
μ (mm^{-1})	1.074
F(000)	344
Independent reflections	2703 [R_{int} = 0.0972, R_{sigma} = 0.1055]
Data/restraints/parameters	2703/2/218
Goodness-of-fit on F^2	1.055
Final R indices [I>=2σ (I)]	R_1 = 0.0566, wR_2 = 0.1374
Final R indices [all data]	R_1 = 0.1039, wR_2 = 0.1771
Largest diff. peak/hole / e Å^{-3}	0.19/-0.18

Table S3. Crystallographic and general data for a single crystal of (*endo*-**1**)(*exo*-**1**) at 200 K.

CCDC deposition No. : 2376887	
Molecular formula	$C_{16}H_{12}O_8$
M_r (g/mol)	332.26
Crystal system	monoclinic
Crystal colour	colorless
Space group	*Pn*
T (K)	200
a (Å)	11.9141(14)
b (Å)	5.3741(4)
c (Å)	11.9214(14)
α (°)	90
β (°)	110.400(13)
γ (°)	90
V (Å^3)	715.43(14)
Z	2
ρ_{calc} (g/cm^3)	1.542
μ (mm^{-1})	1.084
F(000)	344
Independent reflections	2824 [R_{int} = 0.0555, R_{sigma} = 0.0376]
Data/restraints/parameters	2824/2/219
Goodness-of-fit on F^2	1.056
Final R-indices [I>=2σ (I)]	R_1 = 0.0460, wR_2 = 0.1118
Final R-indices [all data]	R_1 = 0.0525, wR_2 = 0.1208
Largest diff. peak/hole / e Å^{-3}	0.20/-0.21

Table S4. Crystallographic and general data for a single crystal of (*endo*-**1**)(*exo*-**1**) at 150 K.

CCDC deposition No. : 2376888	
Molecular formula	$C_{16}H_{12}O_8$
M_r (g/mol)	332.26
Crystal system	monoclinic
Crystal colour	colorless
Space group	*Pn*
T (K)	200
a (Å)	11.8895(13)
b (Å)	5.3562(4)
c (Å)	11.8978(13)
α (°)	90
β (°)	110.247(12)
γ (°)	90
V (Å^3)	710.86(13)
Z	2
ρ_{calc} (g/cm^3)	1.552
μ (mm^{-1})	1.091
F(000)	344
Independent reflections	2803 [R_{int} = 0.0577, R_{sigma} = 0.0396]
Data/restraints/parameters	2803/2/219
Goodness-of-fit on F^2	1.099
Final R indices [I>=2σ (I)]	R_1 = 0.0459, wR_2 = 0.1065
Final R indices [all data]	R_1 = 0.0509, wR_2 = 0.1123
Largest diff. peak/hole / e Å^{-3}	0.19/-0.20

Table S5. Crystallographic and general data for a single crystal of *endo*-**1** at 298 K.

CCDC deposition No. : 2376889	
Molecular formula	$C_8H_6O_4$
M_r (g/mol)	166.13
Crystal system	monoclinic
Crystal colour	colorless
Space group	$P2_1/c$
T (K)	298
a (Å)	11.2763(6)
b (Å)	5.7097(3)
c (Å)	11.4279(6)
α (°)	90
β (°)	103.881(3)
γ (°)	90
V (Å^3)	714.29(7)
Z	4
ρ_{calc} (g/cm^3)	1.545
μ (mm^{-1})	1.086
F(000)	344
Independent reflections	1389 [R_{int} = 0.0603, R_{sigma} = 0.0458]
Data/restraints/parameters	1389/0/109
Goodness-of-fit on F^2	1.029
Final R-indices [I>=2σ (I)]	R_1 = 0.0464, wR_2 = 0.1234
Final R-indices [all data]	R_1 = 0.0582, wR_2 = 0.1377
Largest diff. peak/hole / e Å^{-3}	0.17/-0.18

Table S6. Crystallographic and general data for a single crystal of *endo*-**1** at 291 K.

CCDC deposition No. : 2424954	
Molecular formula	$C_8H_6O_4$
M_r (g/mol)	166.13
Crystal system	monoclinic
Crystal colour	colorless
Space group	$P2_1/c$
T (K)	291
a (Å)	11.2681(11)
b (Å)	5.7140(6)
c (Å)	11.4194(13)
α (°)	90
β (°)	103.858(11)
γ (°)	90
V (Å^3)	713.85(13)
Z	4
ρ_{calc} (g/cm^3)	1.546
μ (mm^{-1})	1.087
F(000)	344
Independent reflections	1465 [R_{int} = 0.0284, R_{sigma} = 0.0353]
Data/restraints/parameters	1465/0/110
Goodness-of-fit on F^2	1.050
Final R-indices [I>=2σ (I)]	R_1 = 0.0444, wR_2 = 0.1072
Final R-indices [all data]	R_1 = 0.0696, wR_2 = 0.1298
Largest diff. peak/hole / e Å^{-3}	0.18/-0.19

Table S7. Crystallographic and general data for a single crystal of *endo*-**1** at 250 K.

CCDC deposition No. : 2376890	
Molecular formula	$C_8H_6O_4$
M_r (g/mol)	166.13
Crystal system	monoclinic
Crystal colour	colorless
Space group	$P2_1/c$
T (K)	250
a (Å)	11.2483(3)
b (Å)	5.6878(1)
c (Å)	11.3895(3)
α (°)	90
β (°)	103.884(1)
γ (°)	90
V (Å^3)	707.39(3)
Z	4
ρ_{calc} (g/cm^3)	1.560
μ (mm^{-1})	1.097
F(000)	344
Independent reflections	1344 [R_{int} = 0.0424, R_{sigma} = 0.0226]
Data/restraints/parameters	1344/0/109
Goodness-of-fit on F^2	1.090
Final R-indices [I>=2σ (I)]	R_1 = 0.0380, wR_2 = 0.0928
Final R-indices [all data]	R_1 = 0.0404, wR_2 = 0.0949
Largest diff. peak/hole / e Å^{-3}	0.20/-0.14

Table S8. Crystallographic and general data for a single crystal of *endo*-**1** at 200 K.[a]

CCDC deposition No. : 2424955	
Molecular formula	$C_8H_6O_4$
M_r (g/mol)	166.13
Crystal system	monoclinic
Crystal colour	colorless
Space group	$P2_1/c$
T (K)	200
a (Å)	11.2234(8)
b (Å)	5.6672(3)
c (Å)	11.3510(8)
α (°)	90
β (°)	103.917(7)
γ (°)	90
V (Å^3)	700.79(8)
Z	4
ρ_{calc} (g/cm^3)	1.575
μ (mm^{-1})	1.103
$F(000)$	344
Independent reflections	1443 [R_{int} = 0.0360, R_{sigma} = 0.0334]
Data/restraints/parameters	1443/0/109
Goodness-of-fit on F^2	1.075
Final R-indices [I>=2σ (I)]	R_1 = 0.0468, wR_2 = 0.1229
Final R-indices [all data]	R_1 = 0.0607, wR_2 = 0.1364
Largest diff. peak/hole / e Å^{-3}	0.23/-0.22

[a]For a 200 K structural study on a different single crystal of *endo*-**1**, see Table S9.

Table S9. Crystallographic and general data for a single crystal of *endo*-**1** at 200 K.[a]

CCDC deposition No. : 2376891	
Molecular formula	$C_8H_6O_4$
M_r (g/mol)	166.13
Crystal system	monoclinic
Crystal colour	colorless
Space group	$P2_1/c$
T (K)	200
a (Å)	11.2298(4)
b (Å)	5.6750(2)
c (Å)	11.3656(4)
α (°)	90
β (°)	103.903(1)
γ (°)	90
V (Å^3)	703.10(4)
Z	4
ρ_{calc} (g/cm^3)	1.569
μ (mm^{-1})	1.103
F(000)	344
Independent reflections	1304 [R_{int} = 0.0362, R_{sigma} = 0.0331]
Data/restraints/parameters	1304/0/109
Goodness-of-fit on F^2	1.054
Final R-indices [I>=2σ (I)]	R_1 = 0.0384, wR_2 = 0.0892
Final R-indices [all data]	R_1 = 0.0413, wR_2 = 0.0914
Largest diff. peak/hole / e Å^{-3}	0.23/-0.15

[a]For a 200 K structural study on a different single crystal of *endo*-**1**, see Table S8.

Table S10. Crystallographic and general data for a single crystal of *endo*-**1** at 150 K.

CCDC deposition No. : 2376892	
Molecular formula	$C_8H_6O_4$
M_r (g/mol)	166.13
Crystal system	monoclinic
Crystal colour	colorless
Space group	$P2_1/c$
T (K)	150
a (Å)	11.1987(6)
b (Å)	5.6558(3)
c (Å)	11.3247(6)
α (°)	90
β (°)	103.949(2)
γ (°)	90
V (Å^3)	696.13(6)
Z	4
ρ_{calc} (g/cm^3)	1.585
μ (mm^{-1})	1.114
F(000)	344
Independent reflections	1285 [R_{int} = 0.0653, R_{sigma} = 0.0547]
Data/restraints/parameters	1285/0/109
Goodness-of-fit on F^2	1.107
Final R-indices [I>=2σ (I)]	R_1 = 0.0647, wR_2 = 0.1823
Final R-indices [all data]	R_1 =0.0679, wR_2 = 0.1882
Largest diff. peak/hole / e Å^{-3}	0.26/-0.25

Table S11. Crystallographic and general data for a single crystal of *endo*-**1** at 100 K.

CCDC deposition No. : 2376893	
Molecular formula	$C_8H_6O_4$
M_r (g/mol)	166.13
Crystal system	monoclinic
Crystal colour	colorless
Space group	$P2_1/c$
T (K)	100
a (Å)	11.1845(9)
b (Å)	5.6420(4)
c (Å)	11.2971(9)
α (°)	90
β (°)	104.169(4)
γ (°)	90
V (Å^3)	691.19(9)
Z	4
ρ_{calc} (g/cm^3)	1.596
μ (mm^{-1})	1.122
F(000)	344
Independent reflections	1298 [R_{int} = 0.0648, R_{sigma} = 0.0554]
Data/restraints/parameters	1298/0/109
Goodness-of-fit on F^2	1.119
Final R-indices [I>=2σ (I)]	R_1 = 0.0714, wR_2 = 0.2019
Final R-indices [all data]	R_1 = 0.0738, wR_2 = 0.2072
Largest diff. peak/hole / e Å^{-3}	0.29/-0.39

Table S12. Crystallographic and general data for a single crystal of (*exo*-**1**)(furan) at 120 K.

CCDC deposition No. : 2376894	
Molecular formula	$C_{20}H_{16}O_9$
M_r (g/mol)	400.33
Crystal system	monoclinic
Crystal colour	colorless
Space group	$P2_1/n$
T (K)	120
a (Å)	11.1990(16)
b (Å)	5.4807(9)
c (Å)	14.732(2)
α (°)	90
β (°)	104.639(15)
γ (°)	90
V (Å^3)	874.9(2)
Z	2
ρ_{calc} (g/cm^3)	1.520
μ (mm^{-1})	1.039
F(000)	416
Independent reflections	1805 [R_{int} = 0.0604, R_{sigma} = 0.0635]
Data/restraints/parameters	1805/16/155
Goodness-of-fit on F^2	1.097
Final R-indices [I>=2σ (I)]	R_1 = 0.0765, wR_2 = 0.1757
Final R-indices [all data]	R_1 = 0.1017, wR_2 = 0.1921
Largest diff. peak/hole / e Å^{-3}	0.30/-0.35

S4.1 ORTEP Figures

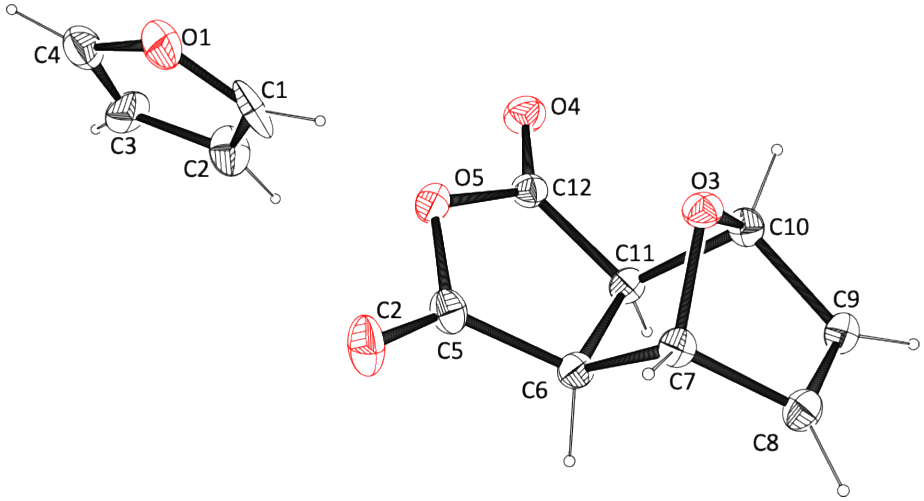

Fig. S12. ORTEP representation of the asymmetric unit for the crystal structure of (*exo*-**1**)(furan) collected at 120 K, with labelling scheme shown. Thermal ellipsoids are shown at 30 % probability level and hydrogen atoms are shown as spheres with an arbitrary radius. For clarity, only one part of the disordered furan molecule is shown.

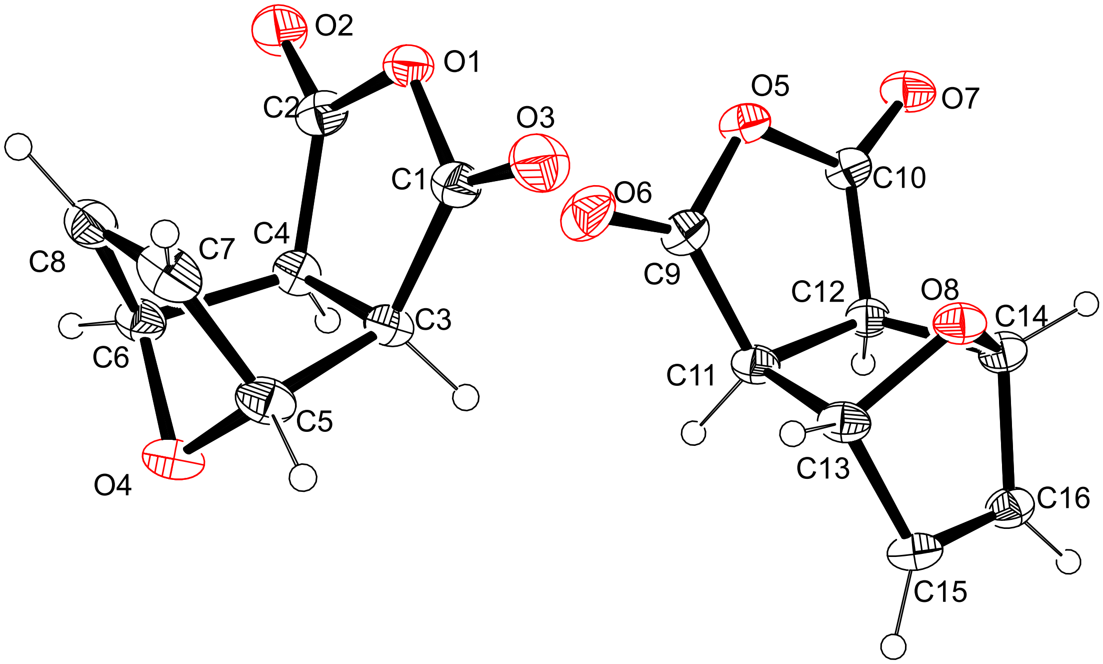

Fig. S13. ORTEP representation of the asymmetric unit for the crystal structure of (*exo*-**1**)(*endo*-**1**) determined at 150 K, with labelling shown. Thermal ellipsoids are shown at 30 % probability level and hydrogen atoms are shown as spheres of arbitrary radius.

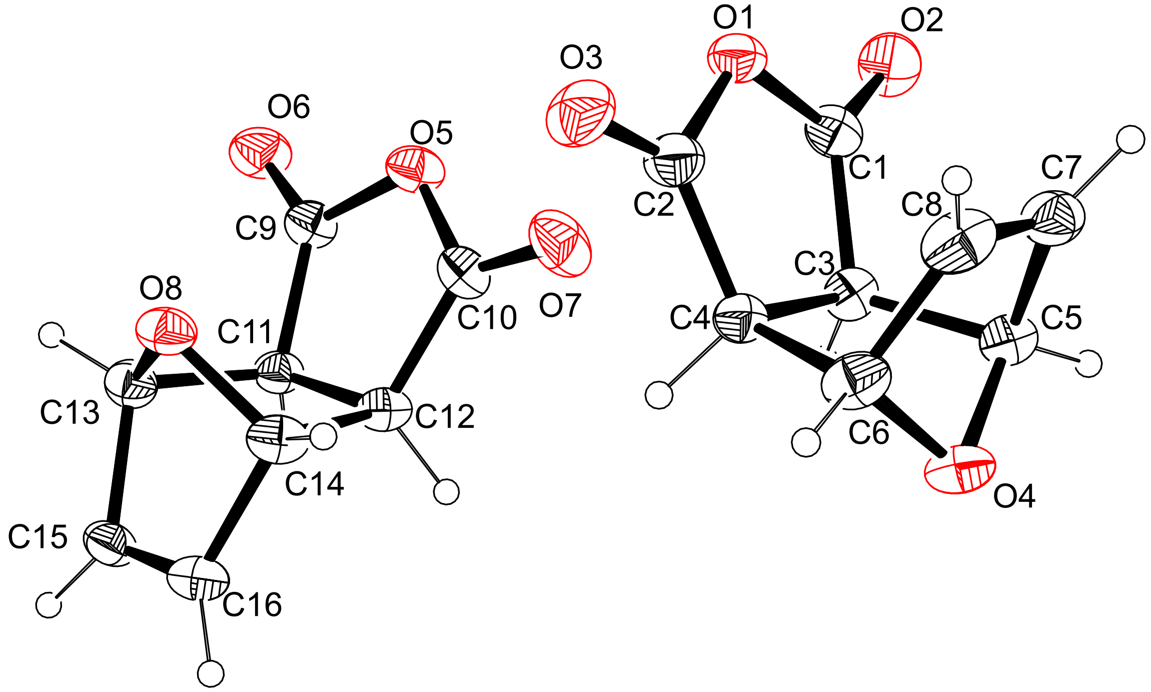

Fig. S14. ORTEP representation of the asymmetric unit for the crystal structure of (*exo*-**1**)(*endo*-**1**) determined at 200 K, with labelling shown. Thermal ellipsoids are shown at 30 % probability level and hydrogen atoms are shown as spheres of arbitrary radius.

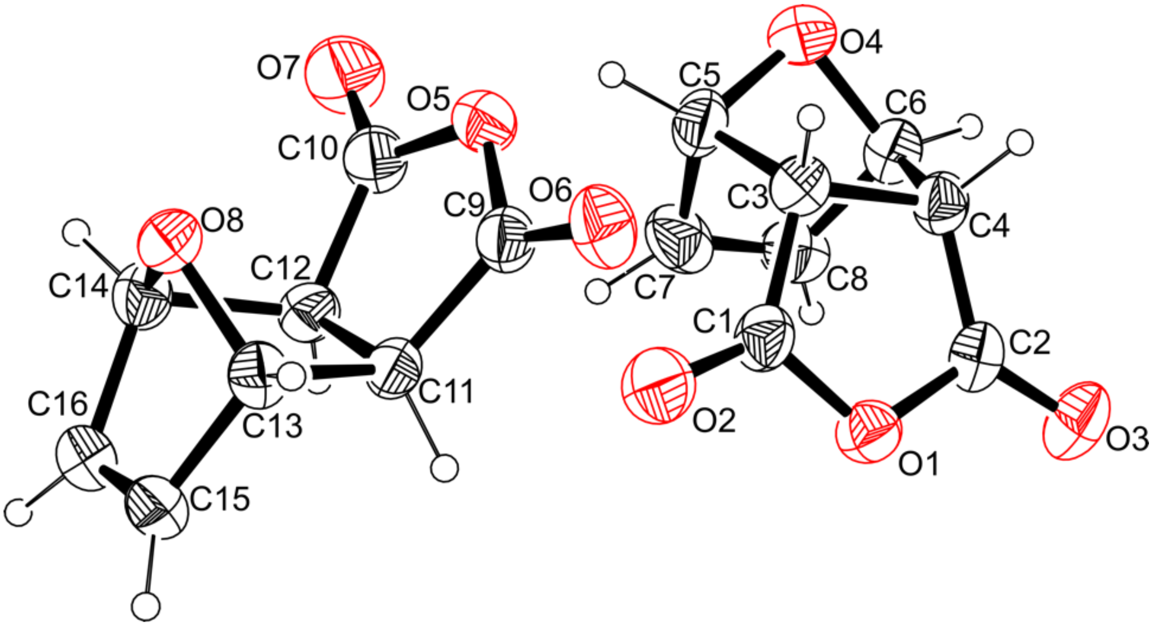

Fig. S15. ORTEP representation of the asymmetric unit for the crystal structure of (*exo*-**1**)(*endo*-**1**) determined at 240 K, with labelling shown. Thermal ellipsoids are shown at 30 % probability level and hydrogen atoms are shown as spheres of arbitrary radius.

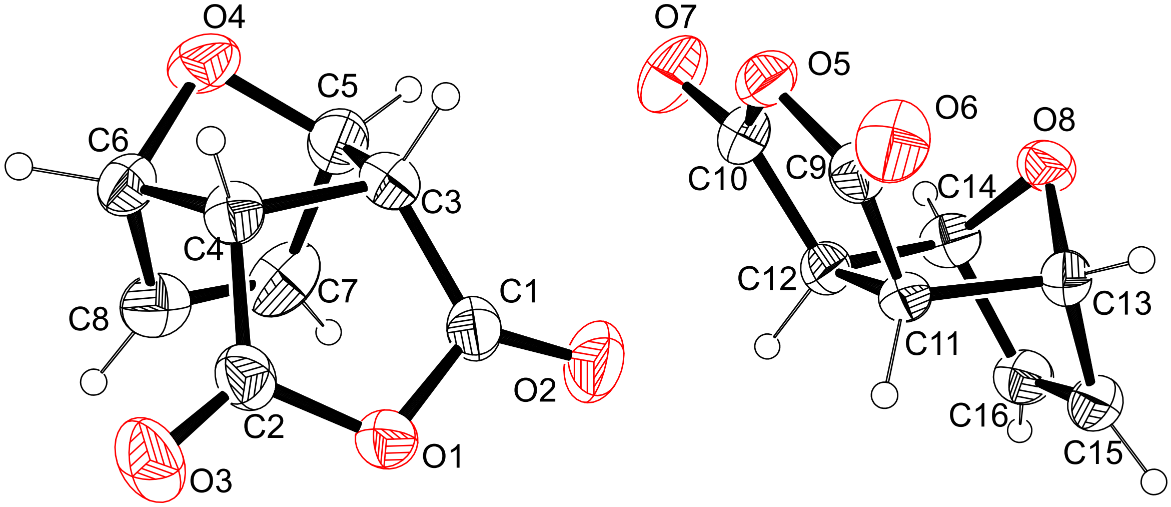

Fig. S16. ORTEP representation of the asymmetric unit for the crystal structure of (*exo*-**1**)(*endo*-**1**) determined at 298 K, with labelling shown. Thermal ellipsoids are shown at 30 % probability level and hydrogen atoms are shown as spheres of arbitrary radius.

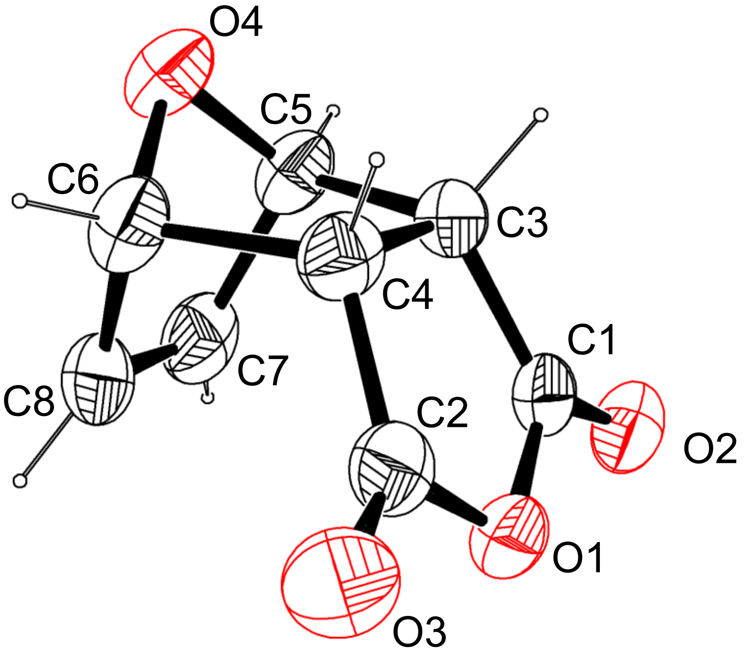

Fig. S17. ORTEP representation of the asymmetric unit of the crystal structure of *endo*-**1**, collected at 298 K showing labelling scheme. Thermal ellipsoids are shown at 30 % probability level and hydrogen atoms are shown as spheres with an arbitrary radius.

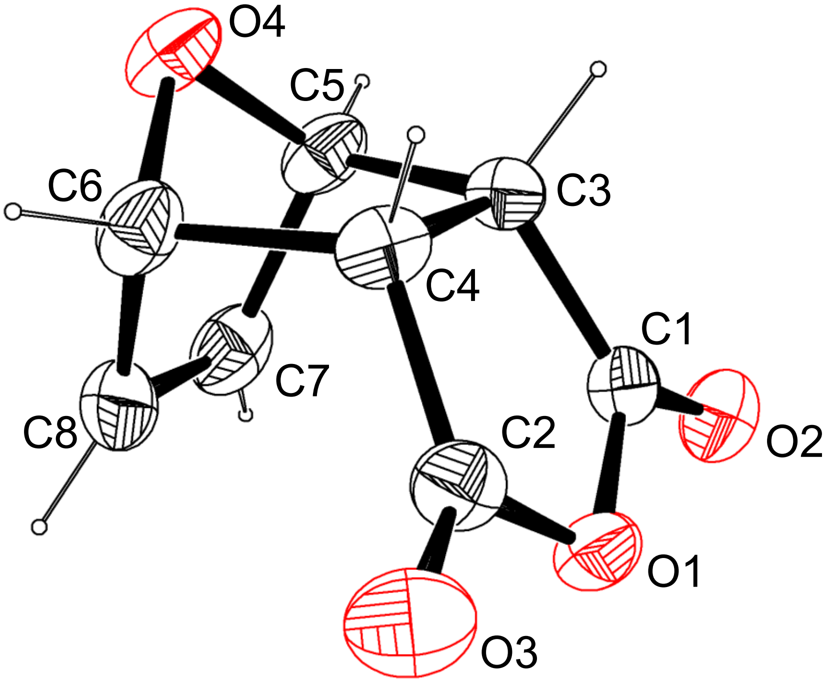

Figure S18. ORTEP representation of the asymmetric unit of the crystal structure of *endo*-**1**, collected at 291 K showing labelling scheme. Thermal ellipsoids are shown at 30 % probability level and hydrogen atoms are shown as spheres with an arbitrary radius.

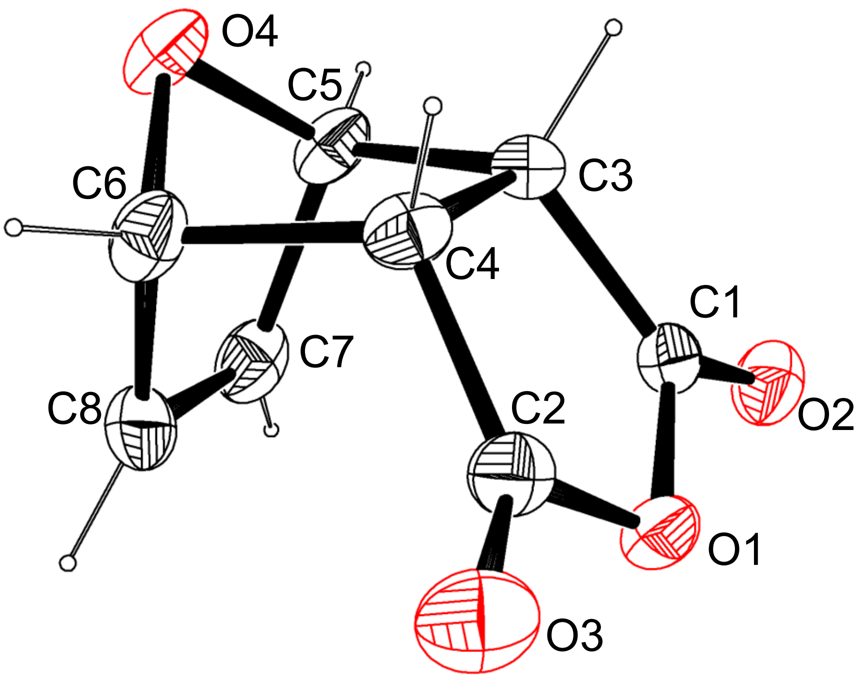

Fig. S19. ORTEP representation of the asymmetric unit of the crystal structure of *endo*-**1**, collected at 250 K showing labelling scheme. Thermal ellipsoids are shown at 30 % probability level and hydrogen atoms are shown as spheres with an arbitrary radius.

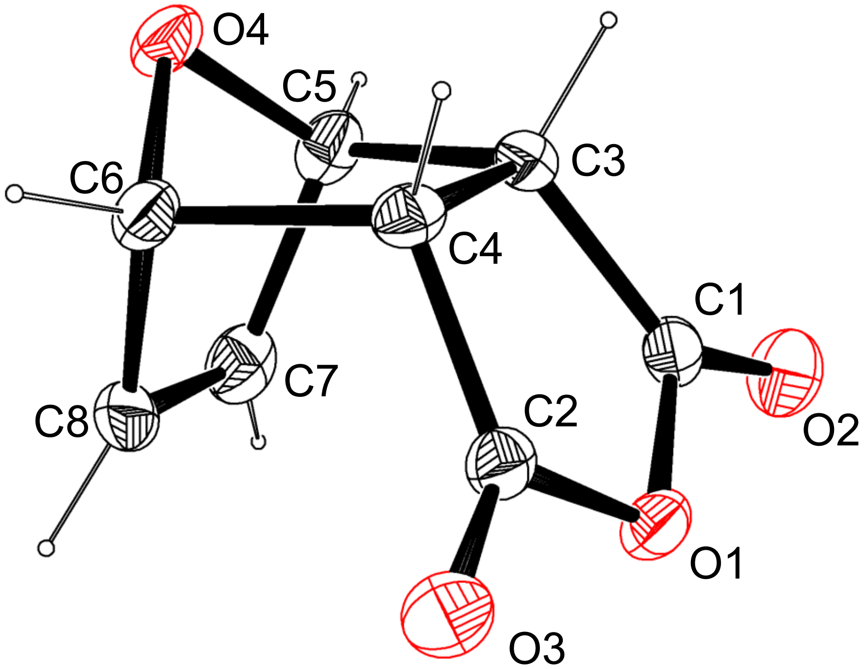

Fig. S20. ORTEP representation of the asymmetric unit of the crystal structure of *endo*-**1**, collected at 200 K showing labelling scheme. Thermal ellipsoids are shown at 30 % probability level and hydrogen atoms are shown as spheres with an arbitrary radius. For reproducibility, the structure analysis at 200K was performed on two different crystals (see also Fig. S21).

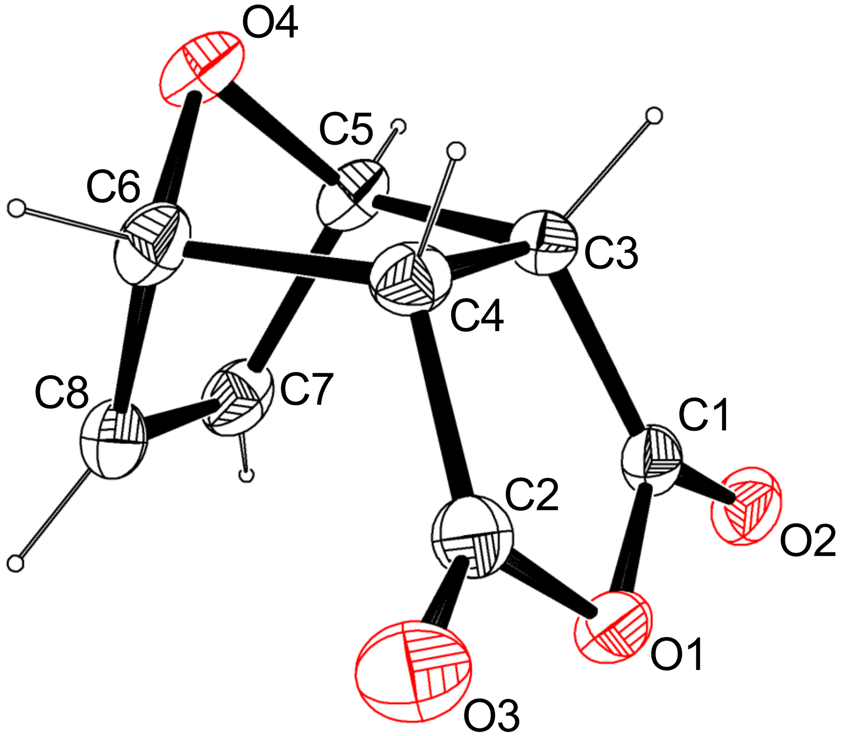

Fig. S21. ORTEP representation of the asymmetric unit of the crystal structure of *endo*-**1**, collected at 200 K showing labelling scheme. Thermal ellipsoids are shown at 30 % probability level and hydrogen atoms are shown as spheres with an arbitrary radius. For reproducibility, the structure analysis at 200K was performed on two different crystals (see also Fig. S20).

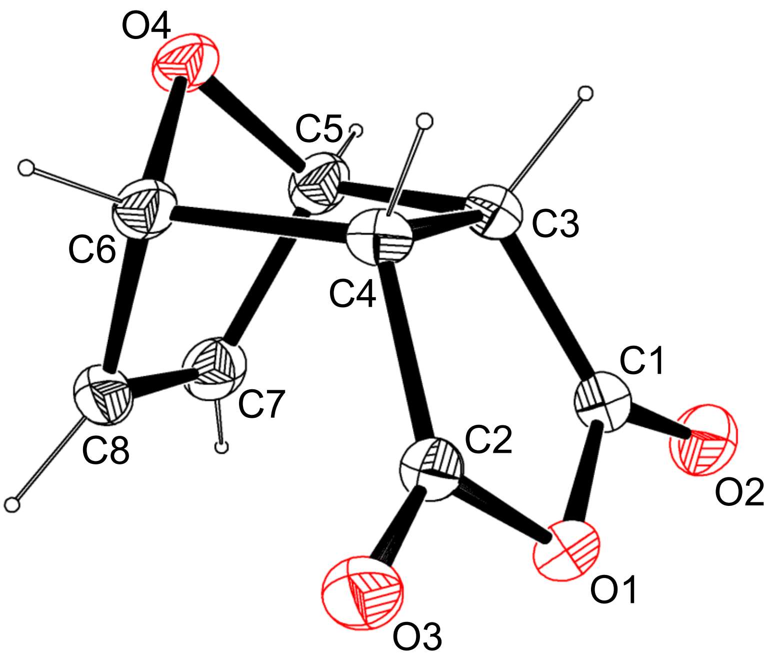

Figure S22. ORTEP representation of the asymmetric unit of the crystal structure of *endo*-**1**, collected at 150 K showing labelling scheme. Thermal ellipsoids are shown at 30 % probability level and hydrogen atoms are shown as spheres with an arbitrary radius.

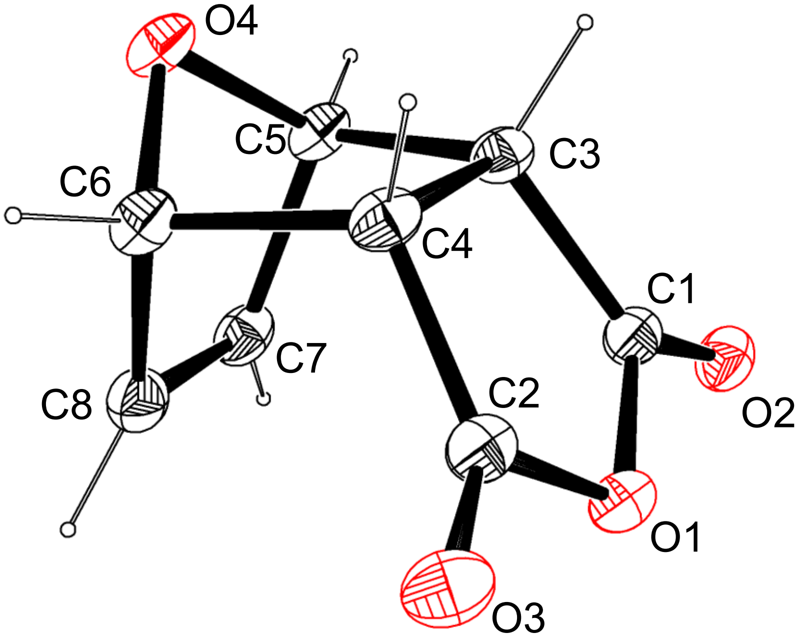

Fig. S23. ORTEP representation of the asymmetric unit of the crystal structure of *endo*-**1**, collected at 100 K showing labelling scheme. Thermal ellipsoids are shown at 30 % probability level and hydrogen atoms are shown as spheres with an arbitrary radius.

S4.2 Principal axis strain resulting from thermal expansion.

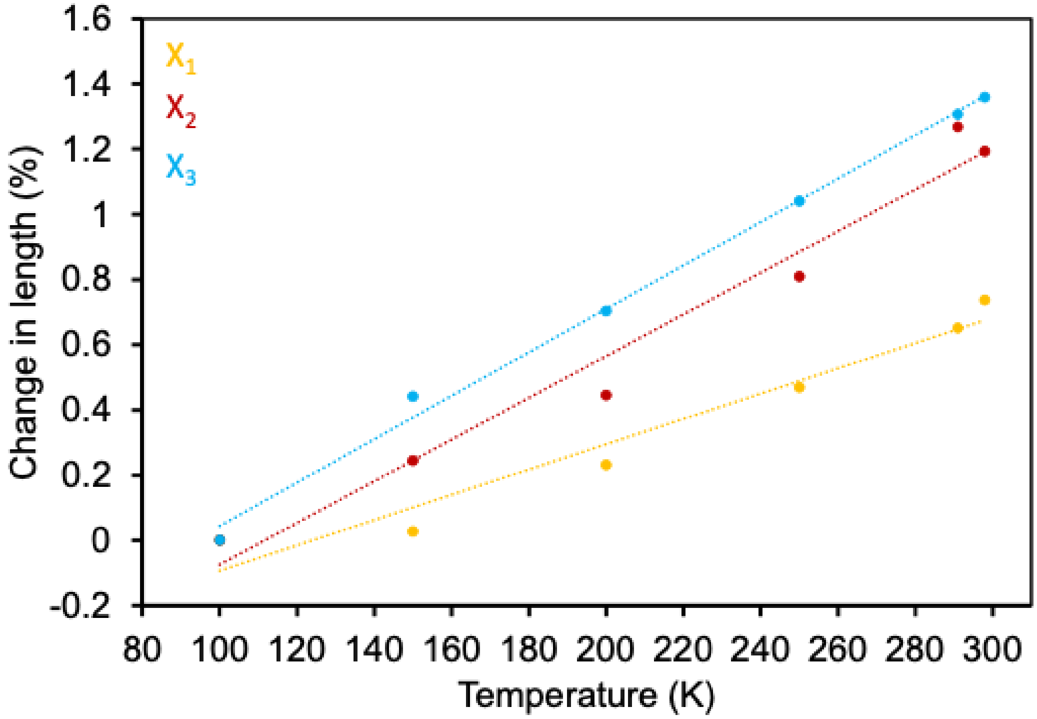

Fig. S24. Plot of the temperature dependence of the principal axis lengths of *endo*-**1**, calculated using PASCal, with linear regression shown.[5]

Table S11. Linear coefficients of thermal expansion for each principal axis of (*endo*-**1**)

Axis	Linear coefficient of thermal expansion **α** (MK^{-1})	Error in coefficient of thermal expansion **σα** (MK^{-1})
X_1	38.719	4.0526
X_2	63.9184	4.1083
X_3	66.5782	2.0818

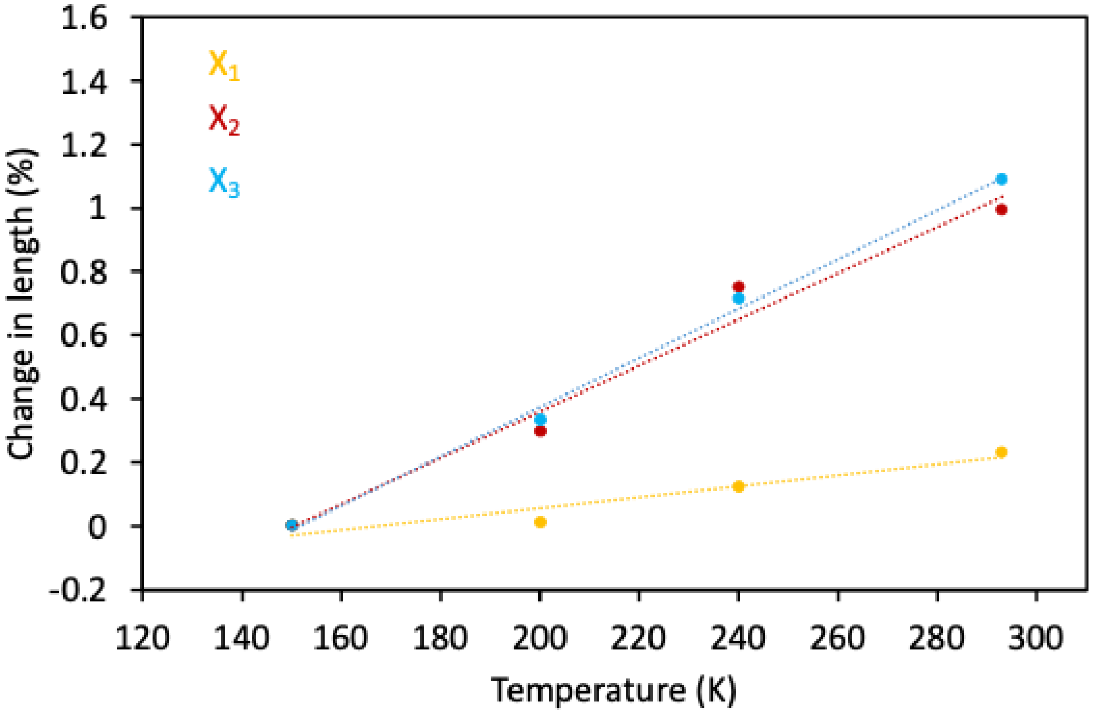

Fig. S25. Temperature dependence of the principal axes lengths for (*endo*-**1**)(*exo*-**1**), calculated using PASCal, with linear regression shown.[5]

Table S12. Linear coefficients of thermal expansion for each principal axis of (*endo*-**1**)(*exo*-**1**)

Axis	Linear coefficient of thermal expansion **α** (MK^{-1})	Error in coefficient of thermal expansion **σα** (MK^{-1})
X_1	17.1829	2.4
X_2	72.7227	3.4
X_3	77.5746	1.3

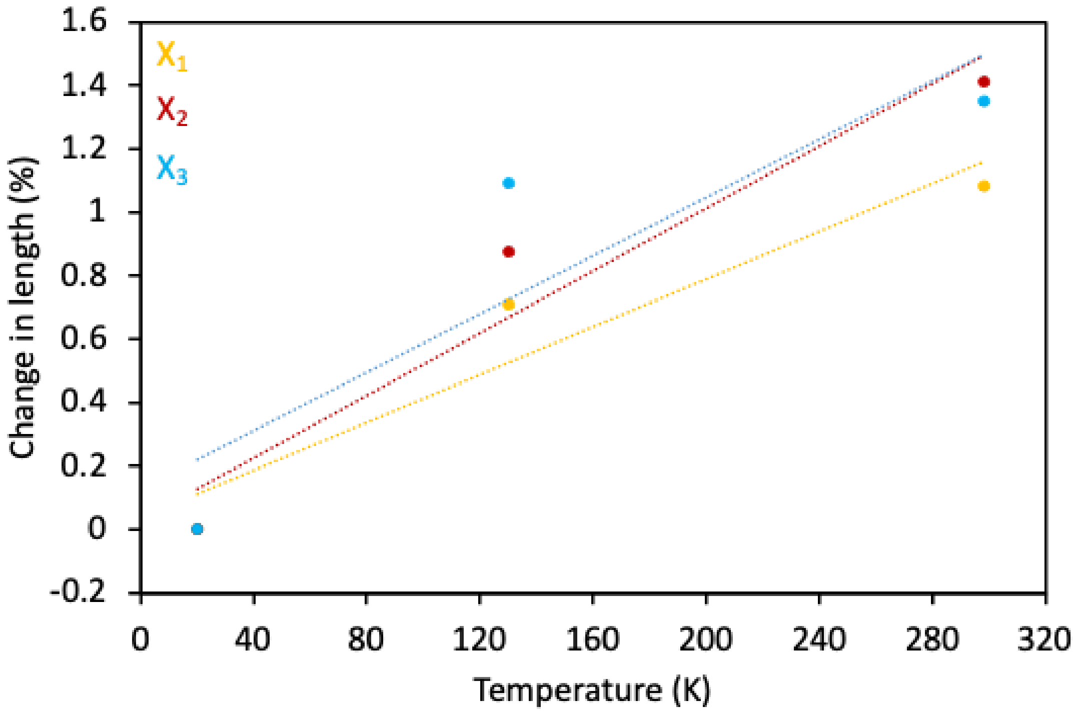

Fig. S26. Temperature dependence of the principal axes lengths for (*exo*-**1**), calculated using PASCal, with linear regression shown.[5]

Table S13. Linear coefficients of thermal expansion for each principal axis of (*exo*-**1**)

Axis	Linear coefficient of thermal expansion **α** (MK^{-1})	Error in coefficient of thermal expansion **σα** (MK^{-1})
X_1	37.6063	4.7
X_2	49.1942	5.3
X_3	45.9075	9.3

S5. ^{1}H- and ^{13}C-NMR spectra

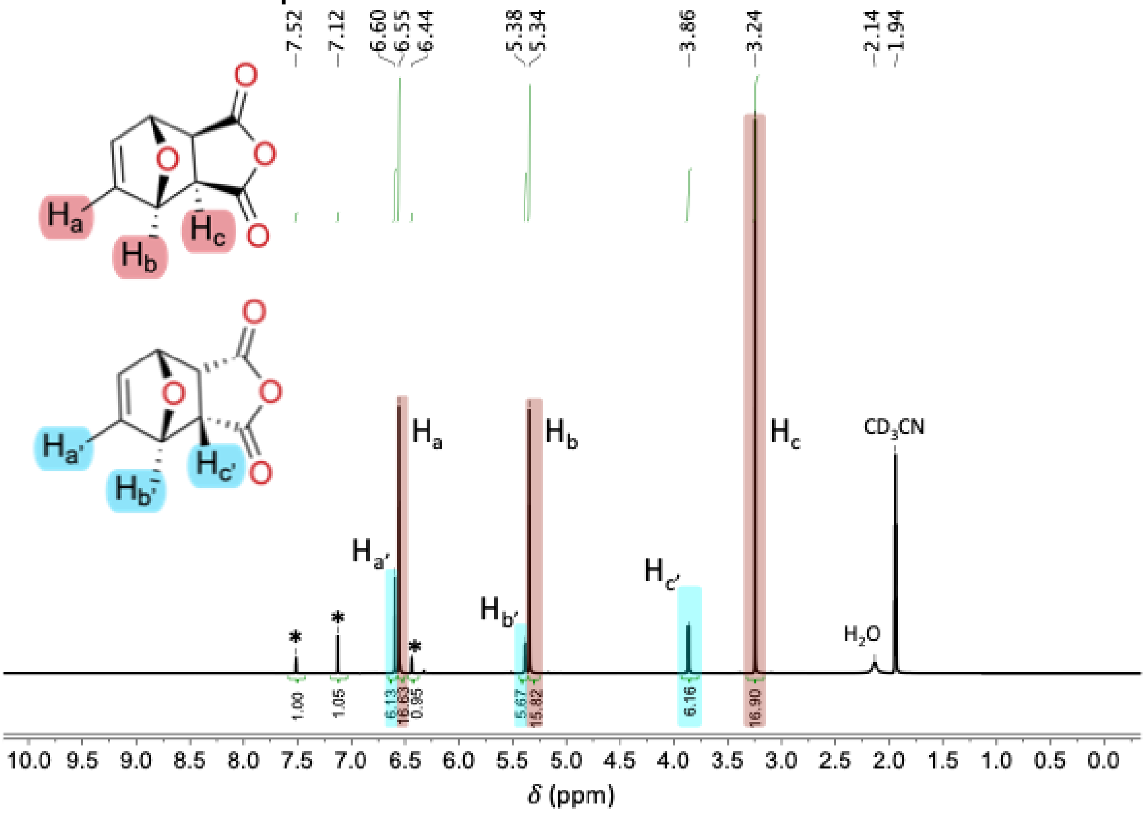

Fig. S27. ^{1}H NMR spectrum in CD_3CN (δ1.94 ppm and H_2O δ 2.14 ppm) following crystallisation of maleic anhydride and furan at 4 °C, resulting in a mixture of *endo*-**1** and *exo*-**1** in ca. 3 : 8 ratio. *Note*: chemical composition of mixture corresponds to ca. 24 % *endo*-**1** (δ 6.60, 5.38, 3.86 ppm), and 67 % *exo*-**1** (δ 6.55, 5.34, 3.24 ppm), 4 % maleic anhydride (δ 7.12 ppm), 4 % furan (δ 7.52, 6.44 ppm) and trace maleic acid (δ 6.33 ppm), marked with asterisks. Samples were collected within 10 minutes of sample dissolution and recorded at 25 °C.

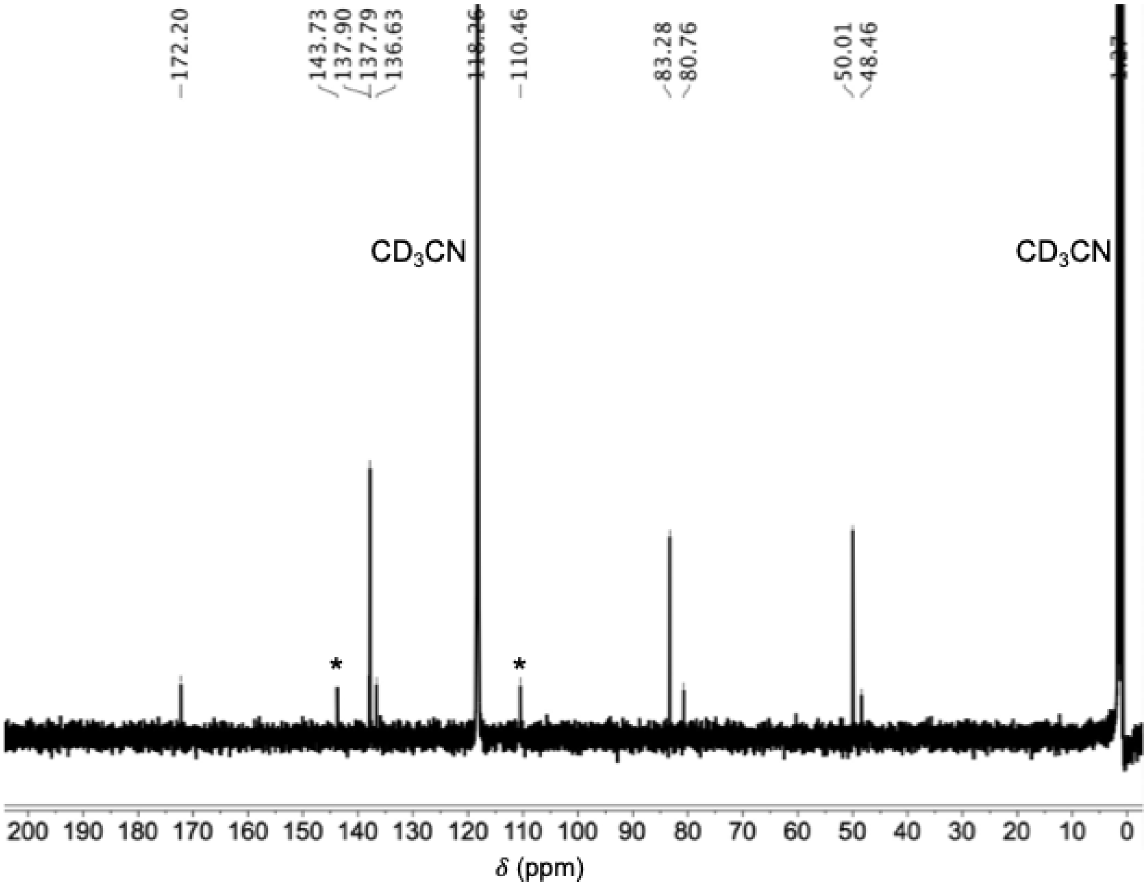

Fig. S28. ^{13}C NMR spectrum in CD_3CN (δ 118.26, 1.27 ppm) of a mixture of maleic anhydride and furan following crystallisation at 0 °C, resulting in a mixture of *endo*-**1** (δ 137.90, 136.63, 80.76, 48.46 ppm) and *exo*-**1** (*δ* 172.20, 137.79, 83.28, 50.01 ppm). Residual furan (δ 143.73 and 110.46 ppm) is marked with asterisks. Samples were collected within 10 minutes of sample dissolution and recorded at 25 °C.

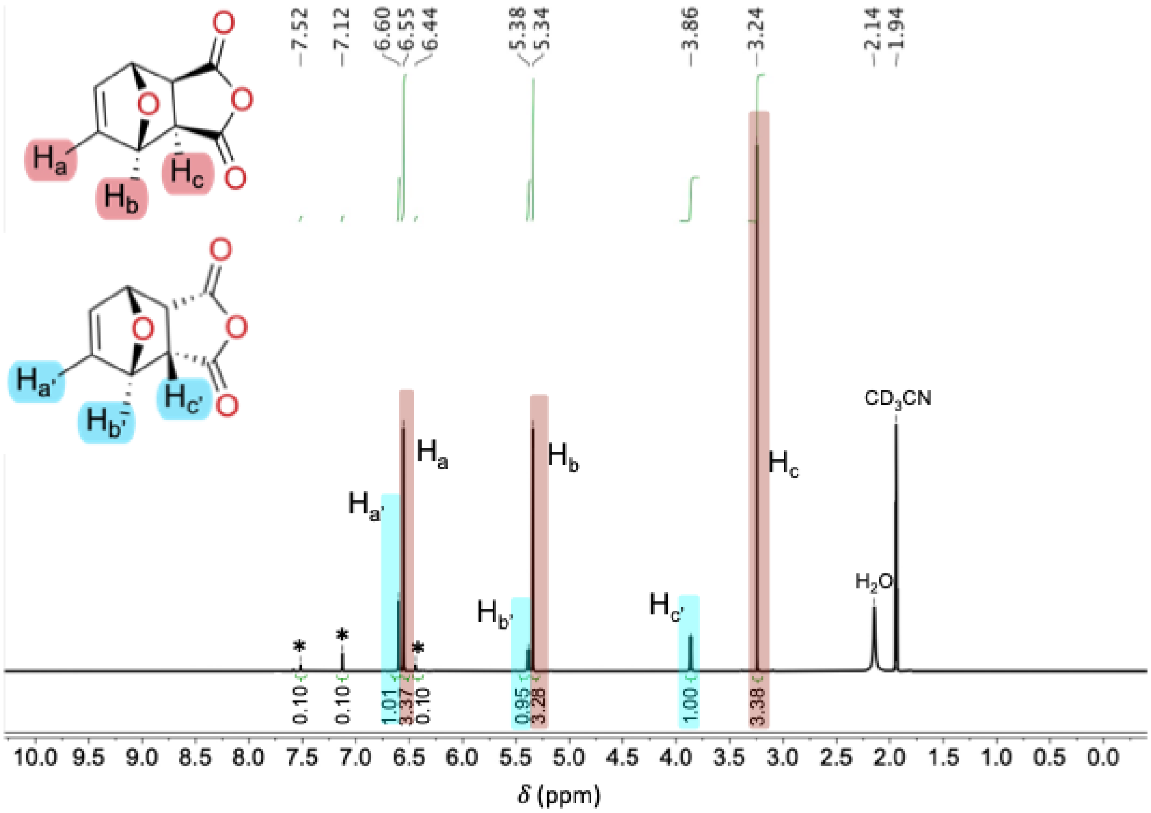

Fig. S29. ^{1}H NMR spectrum of reaction mixture of maleic anhydride and furan at room temperature in CD_3CN (δ 2.13 (H_2O) and 1.94 ppm), resulting in a mixture of *endo*-**1** and *exo*-**1** in a ratio of ca. 2 : 7 *endo*-**1** : *exo*-**1**. *Note*: Chemical composition of mixture corresponds to ca. 2 % maleic anhydride (δ 7.12 ppm), 2 % furan (δ 7.52, 6.44 ppm), 22 % *endo*-**1** (δ 6.60, 5.38, 3.86 ppm), and 74 % *exo*-**1** (δ 6.55, 5.34, 3.24 ppm). Samples were collected within 10 minutes of sample dissolution and recorded at 25 °C.

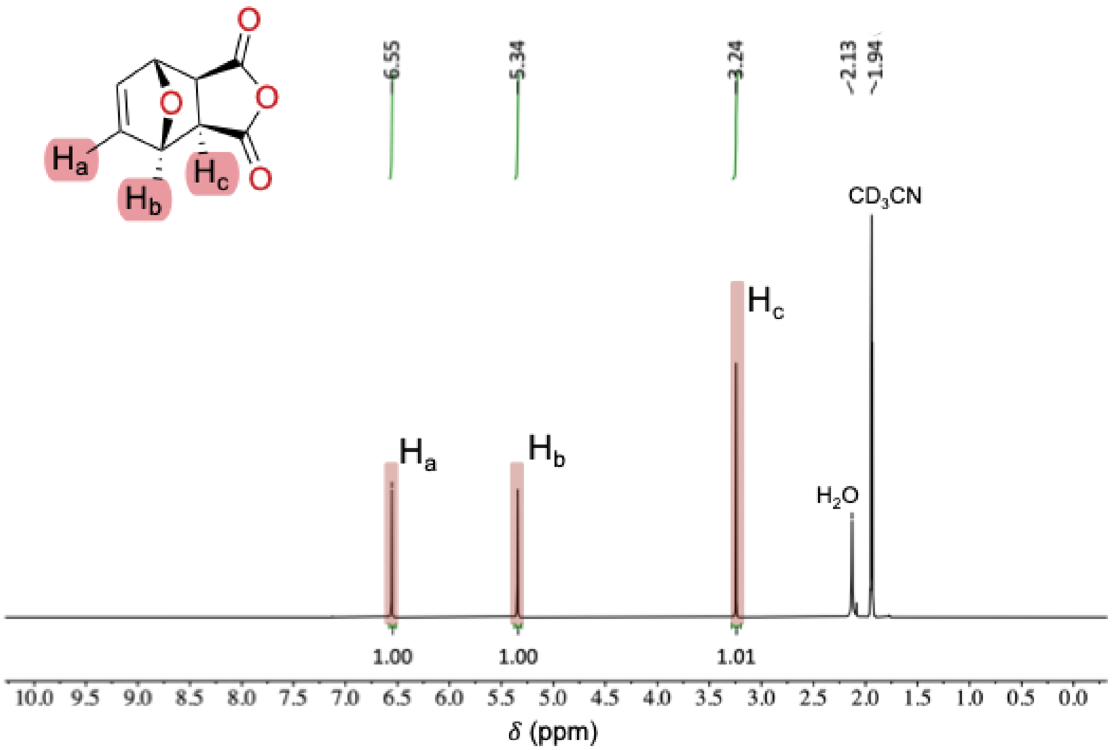

Fig. S30. ^{1}H NMR spectrum of *exo*-**1** (δ 6.55, 5.34, 3.24 ppm) in CD_3CN (δ 2.13 (H_2O) and 1.94 ppm). Samples were collected within 10 minutes of sample dissolution and recorded at 25 °C.

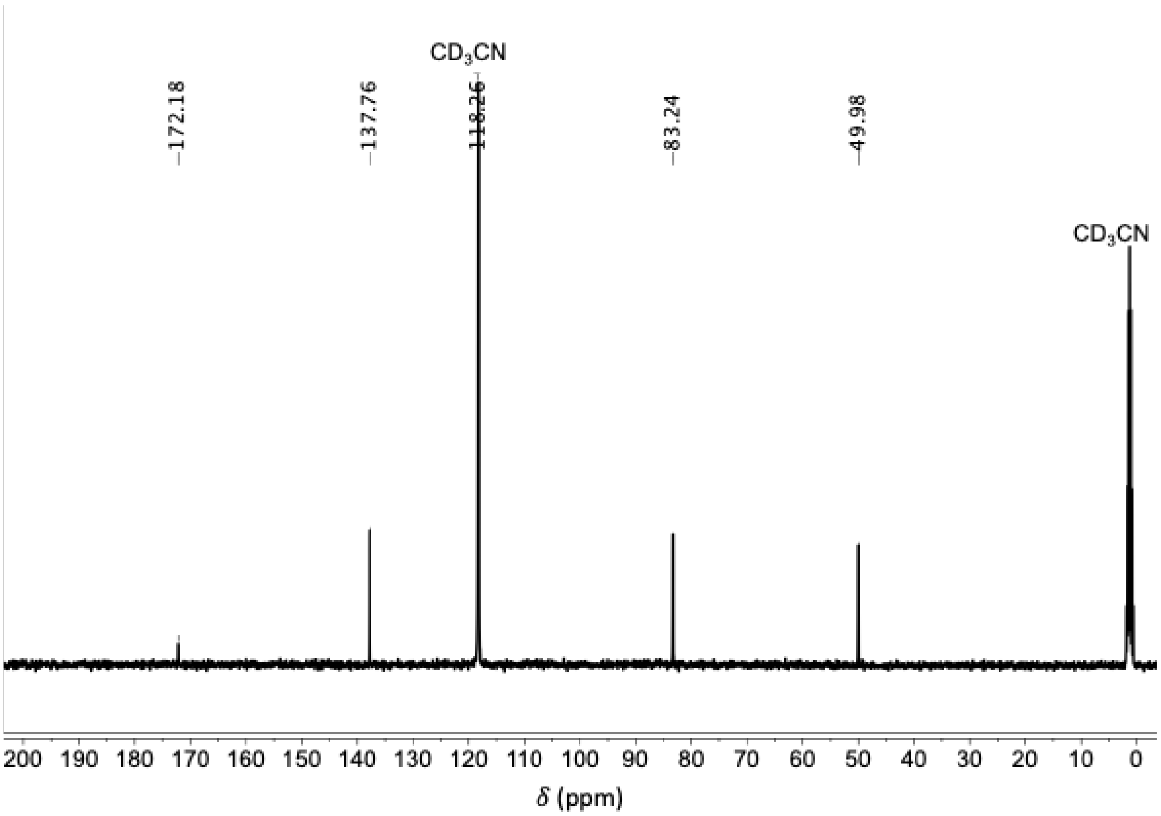

Fig. S31. ^{13}C NMR spectrum of *exo*-**1** (δ 172.18, 137.76, 83.24, 49.98 ppm) in CD_3CN (δ 118.26, 1.26 ppm). Samples were collected within 10 minutes of sample dissolution and recorded at 25 °C.

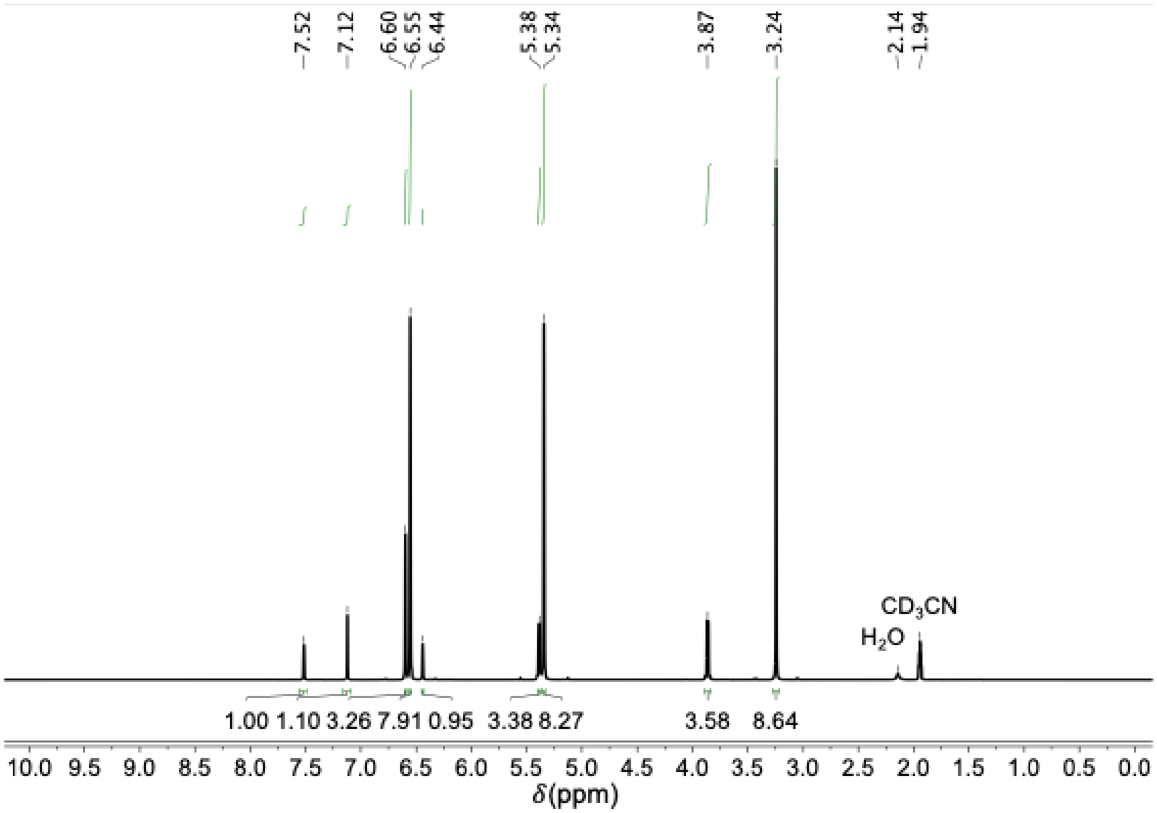

Fig. S32. ^{1}H NMR spectrum of the solid mixture produced by the neat reaction of maleic anhydride (3 mmol) and furan (3 mmol) at room temperature for 24 hours, dissolved in CD_3CN (δ 2.13 (H_2O) and 1.94 ppm); measured ratio of *ca.* 5:12 *endo*-**1**:*exo*-**1**. Chemical composition of mixture corresponds to *ca.* 7 % furan (δ 7.52, 6.44 ppm), 8 % maleic anhydride (δ 7.12 ppm), 25 % *endo*-**1** (δ 6.60, 5.38, 3.86 ppm), and 60 % *exo*-**1** (δ 6.55, 5.34, 3.24 ppm). Spectra were measured within 10 minutes of sample dissolution and recorded at 25 °C.

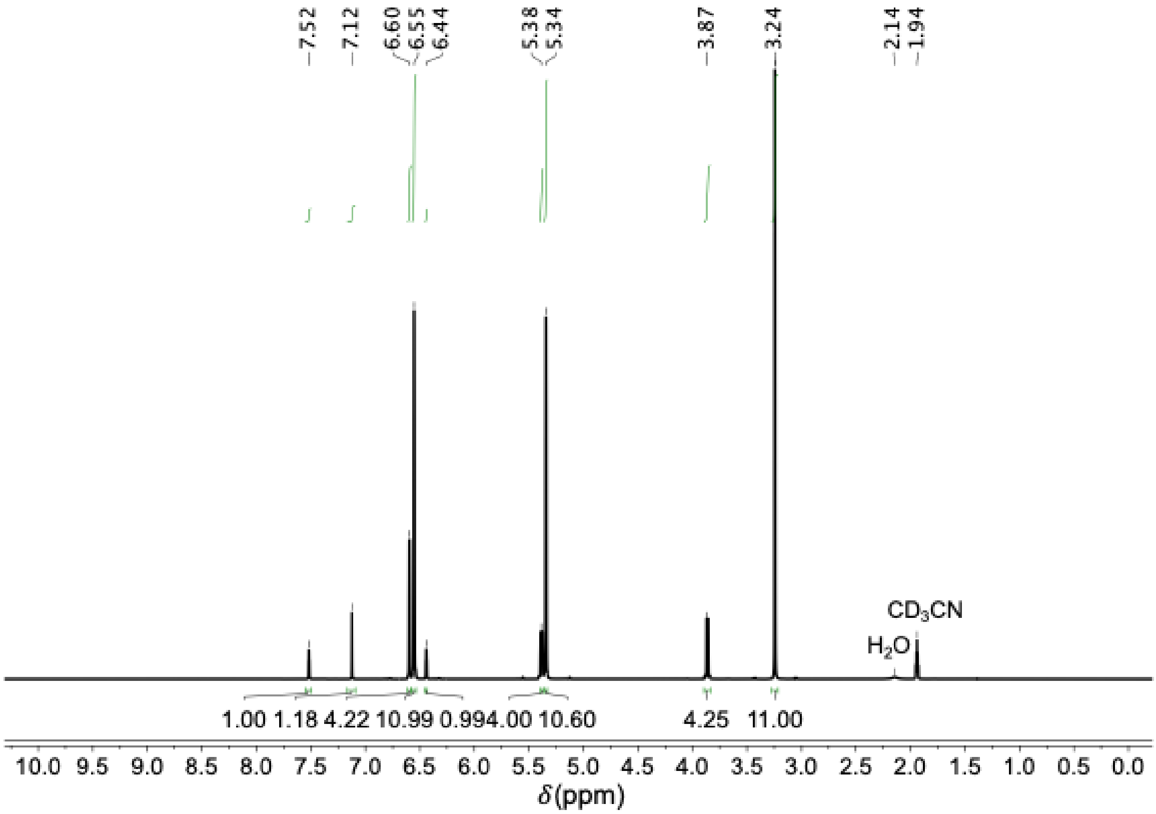

Fig. S33. ^{1}H NMR spectrum of the solid mixture produced by the neat reaction of maleic anhydride (3 mmol) and furan (3 mmol) at room temperature for 24 hours, dissolved in CD_3CN (δ 2.13 (H_2O) and 1.94 ppm); measured ratio of *ca.* 3:8 *endo*-**1**:*exo*-**1**. Chemical composition of mixture corresponds to *ca.* 6 % furan (δ 7.52, 6.44 ppm), 7 % maleic anhydride (δ 7.12 ppm), 24 % *endo*-**1** (δ 6.60, 5.38, 3.86 ppm), and 63 % *exo*-**1** (δ 6.55, 5.34, 3.24 ppm). Spectra were measured within 10 minutes of sample dissolution and recorded at 25 °C.

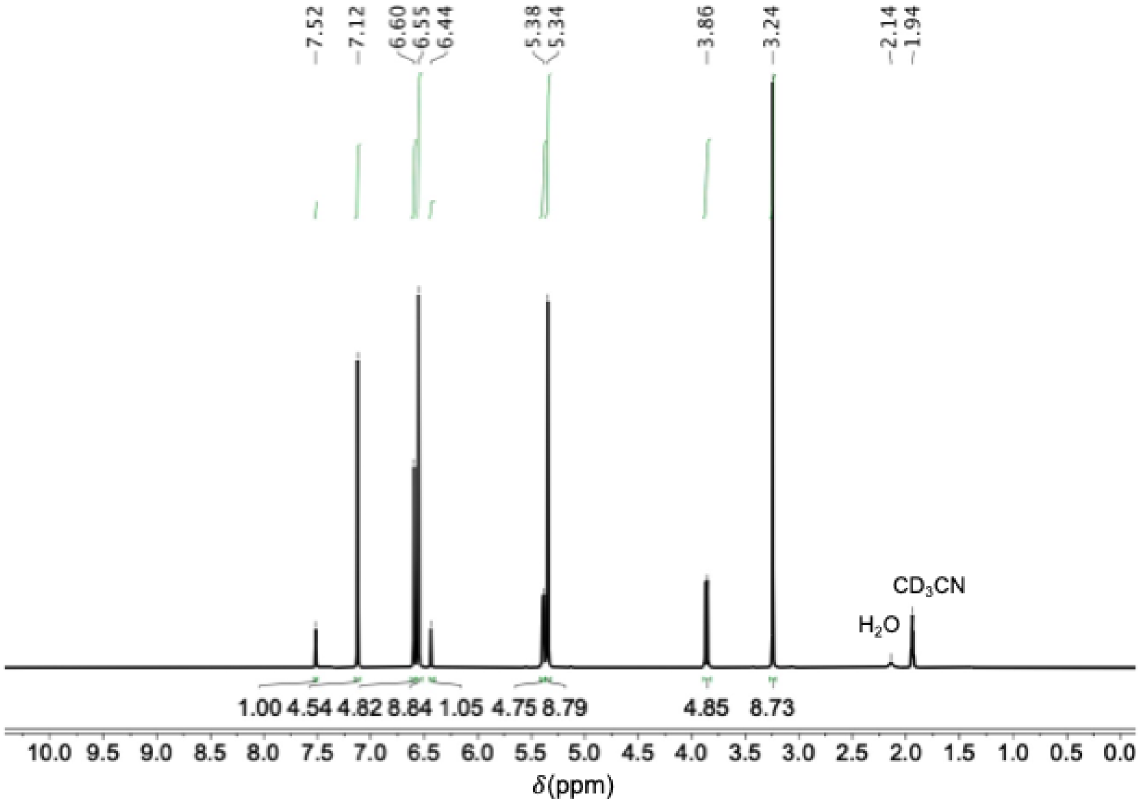

Fig. S34. ^{1}H NMR spectrum of the solid mixture produced by the neat reaction of maleic anhydride (3 mmol) and furan (3 mmol) at 4°C for 24 hours, dissolved in CD_3CN (δ 2.13 (H_2O) and 1.94 ppm); measured ratio of *ca.* 5:9 *endo*-**1**:*exo*-**1**. Chemical composition of mixture corresponds to *ca.* 5 % furan (δ 7.52, 6.44 ppm), 24 % maleic anhydride (δ 7.12 ppm), 25 % *endo*-**1** (δ 6.60, 5.38, 3.86 ppm), and 46 % *exo*-**1** (δ 6.55, 5.34, 3.24 ppm). Spectra were measured within 10 minutes of sample dissolution and recorded at 25 °C.

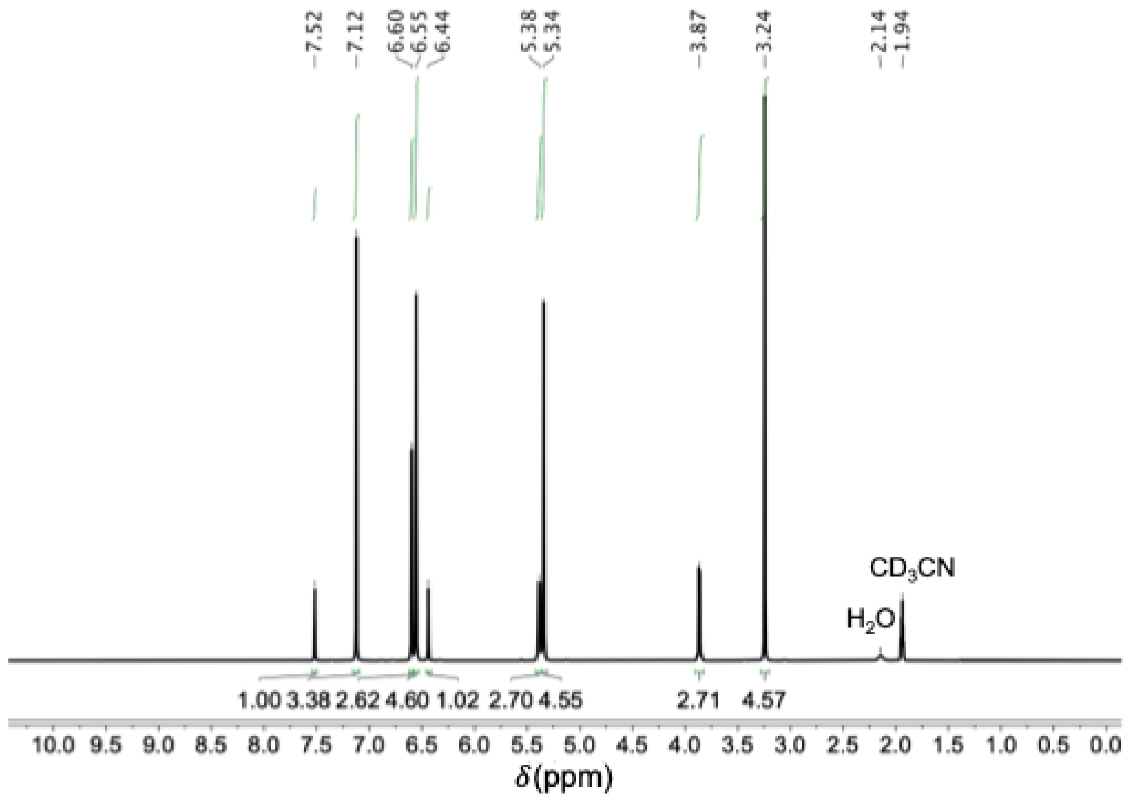

Fig. S35. ^{1}H NMR spectrum of the solid mixture produced by the neat reaction of maleic anhydride (3 mmol) and furan (3 mmol) at 4°C for 24 hours, dissolved in CD_3CN (δ 2.13 (H_2O) and 1.94 ppm); measured ratio of *ca.* 3:5 *endo*-**1**:*exo*-**1**. Chemical composition of mixture corresponds to *ca.* 9 % furan (δ 7.52, 6.44 ppm), 29 % maleic anhydride (δ 7.12 ppm), 23 % *endo*-**1** (δ 6.60, 5.38, 3.86 ppm), and 39 % *exo*-**1** (δ 6.55, 5.34, 3.24 ppm). Spectra were measured within 10 minutes of sample dissolution and recorded at 25 °C.

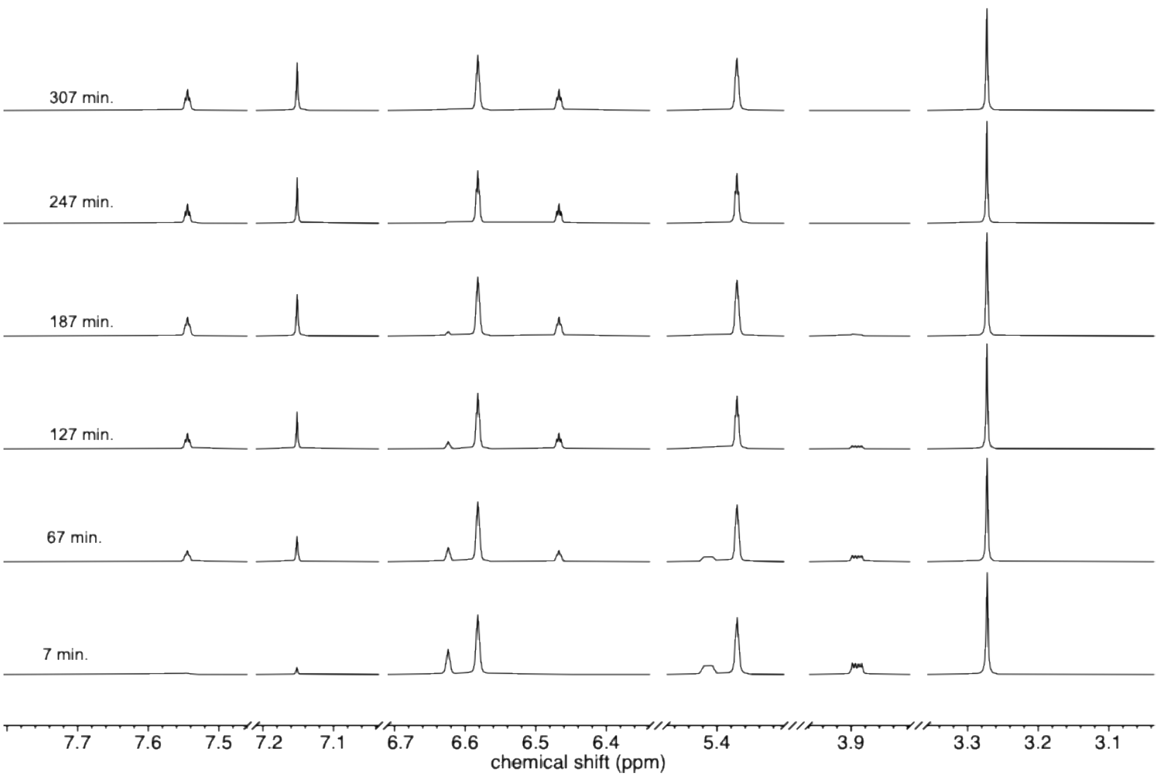

Fig. S36. Time-resolved ^{1}H NMR spectra for a mixture resulting from the neat reaction of maleic anhydride (3 mmol) with furan (3 mmol) at room temperature for 19 hours. The solidified reaction mixture (44 mg) was dissolved in 1 mL CD_3CN, and spectra measured with a delay of 20 minutes. For clarity, only spectra collected at 1 hour intervals over the first 5 hours of the experiment are shown above. The first spectrum (bottom) was measured ca. 7 minutes after the sample was dissolved in CD_3CN.

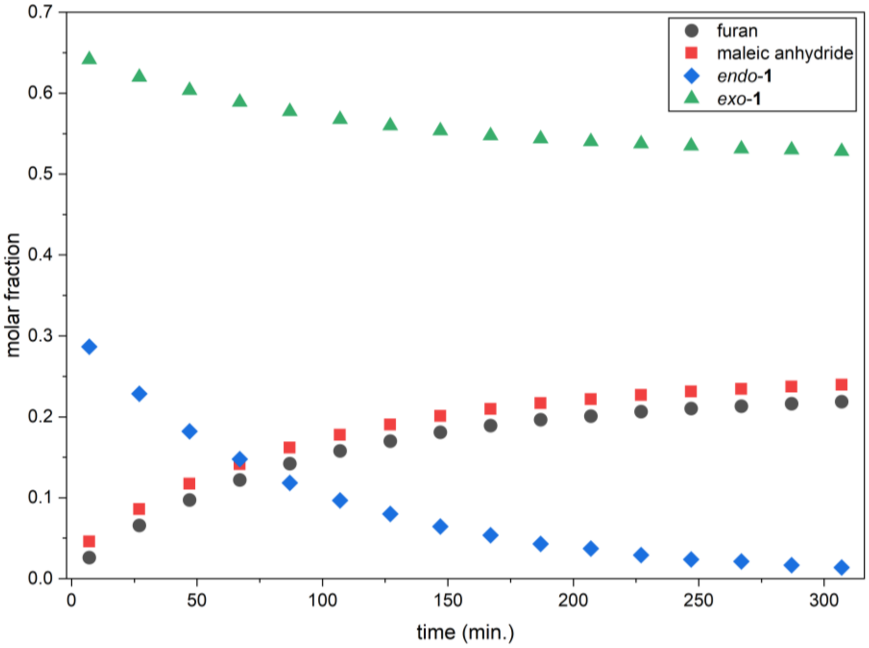

Fig. S37. The time-dependent changes in the molar fractions of *endo*-**1**, *exo*-**1**, furan, and maleic anhydride determined by integration of characteristic signals for each compound in time-dependent ^{1}H NMR spectra (for experimental details see Section S1.5, and for example ^{1}H NMR spectra see Figure S36).

S6. Thermal microscopy

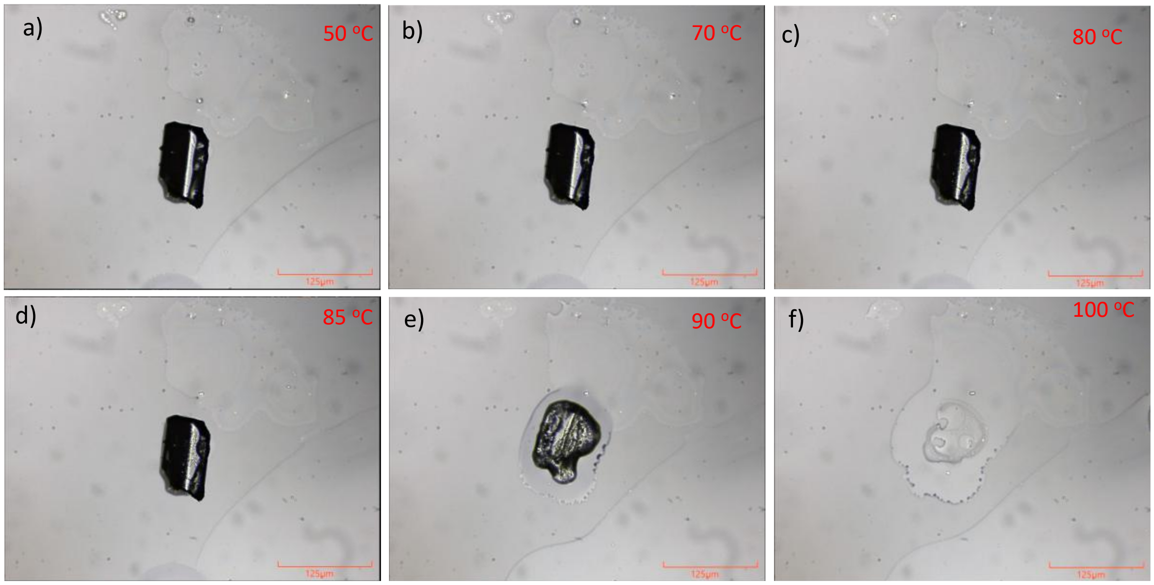

Fig. S38. Hot-stage microscopy images of a crystal of *endo*-**1** at: a) 50 °C, b) 70 °C, c) 80 °C, d) 85 °C, e) 90 °C, and f) 100 °C. Prior to measurement, the crystal composition was verified by single-crystal X-ray diffraction.

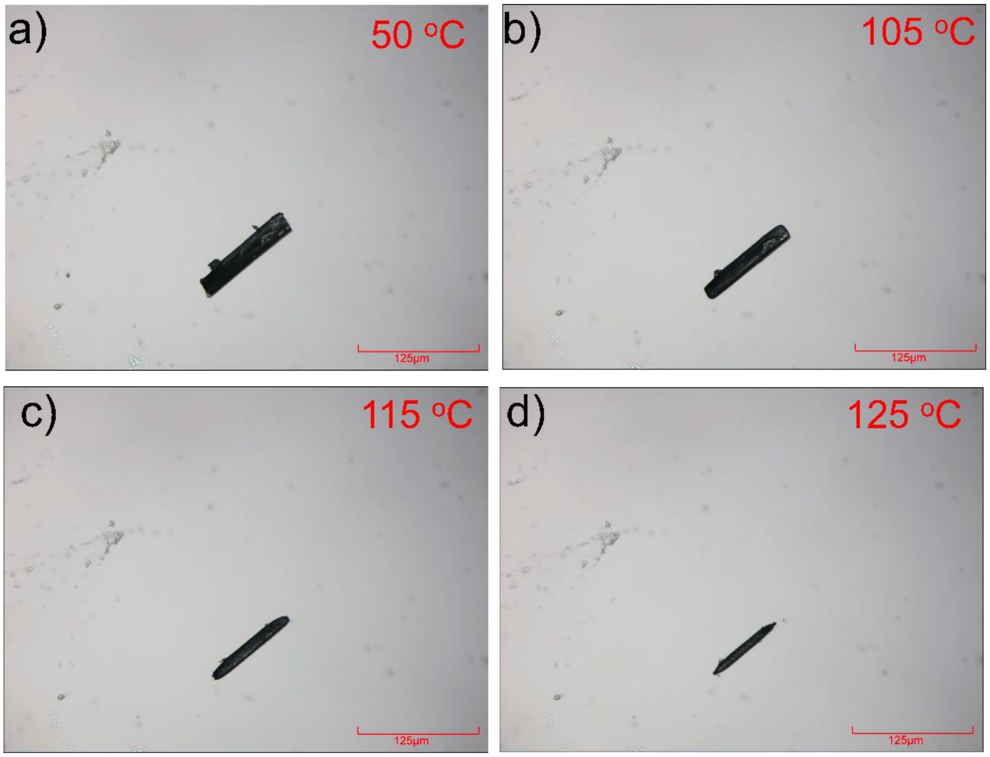

Fig. S39. Hot-stage microscopy images of an *exo*-**1** crystal at: a) 50 °C, b) 105 °C, c) 115 °C, d) 125 °C. Prior to measurement, crystal composition was verified by single-crystal X-ray diffraction.

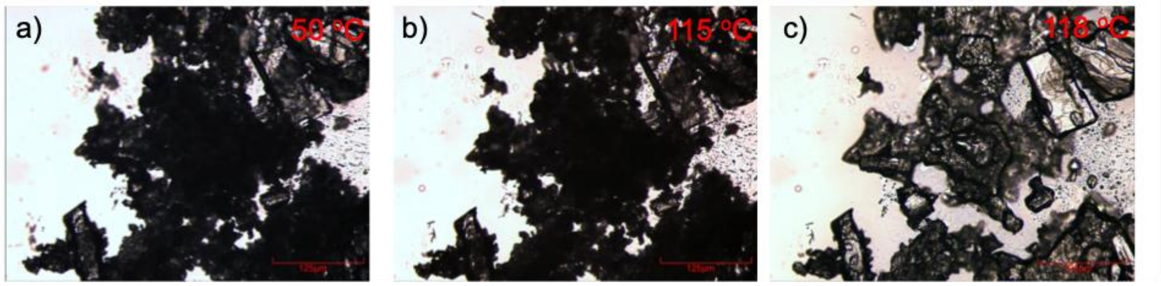

Fig. S40. Hot-stage microscopy images of a polycrystalline sample of *exo*-**1** placed between loose cover slips at a) 50 °C, b) 115 °C, and b) 118 °C. Prior to measurement, the composition was verified by PXRD analysis.

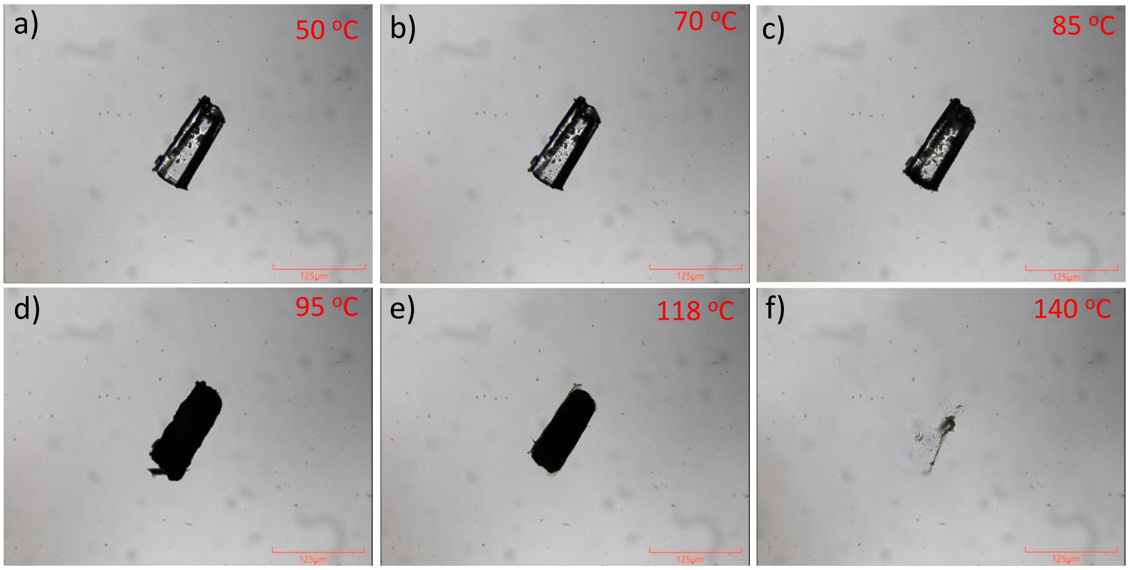

Fig. S41. Hot-stage microscopy images of a crystal of (*endo*-**1**)(*exo*-**1**) taken at: a) 50 °C, b) 70 °C, c) 85 °C, d) 95 °C, e) 118 °C, and e) 140 °C. Prior to measurement, the composition of the initial crystal was confirmed by single-crystal X-ray diffraction.

S7. Thermal microscopy videos

Movie S1 (separate file). *endo*-1: Demonstration of the thermal behaviour of a crystal of *endo*-1 upon heating at 10 °C/min. At 100 ºC, the thermal stage is held at a constant temperature of 100 °C, and the light is turned off to allow Raman spectra to be recorded on the residue.

Movie S2 (separate file). *endo*-1_*exo*-1*:* Demonstration of the thermal behaviour of a crystal of (*exo*-1)(*endo*-1) upon heating at 10 °C/min. At 100 ºC, the thermal stage is held at a constant temperature of 100 °C, and the light is turned off to allow Raman spectra to be recorded on the residue.

Movie S3 (separate file). *exo*-1: Demonstration of the thermal behaviour of a crystal of *exo*-1 upon heating at 10 °C/min. At 100 °C, the thermal stage is held at a constant temperature of 100 °C, and the light is turned off to allow Raman spectra to be recorded on the residue.

Movie S4 (separate file). *exo*-1_covered: Demonstration of the thermal behaviour of a polycrystalline sample of *exo*-1, upon heating at 10 °C/min. Sample is held between two loose glass cover slips.

Movie S5 (separate file). *exo*-1_alt: Demonstration of the thermal behaviour of a larger crystal of *exo*-1, upon heating at 10 ºC/min.

S8. Raman spectra

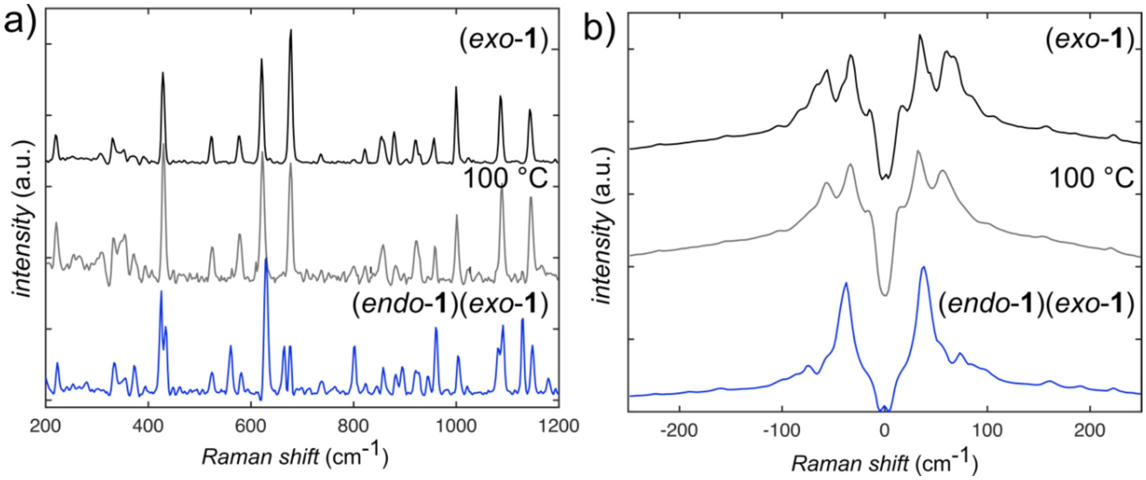

Fig. S42. Raman spectra of (*endo*-**1**)(*exo*-**1**) (blue), (*endo*-**1**)(*exo*-**1**) after heating to 100 °C (gray), and the *exo*-**1** reference spectrum (black, measured at 150 K) over the range: a) 200 – 1200 cm^{-1} and b) the low-frequency Raman region. The spectra indicate that after heating (*endo*-**1**)(*exo*-**1**) to 100 °C, the remaining solid is *exo*-**1**.

S9. Thermogravimetric analysis / differential scanning calorimetry (TGA/DSC)

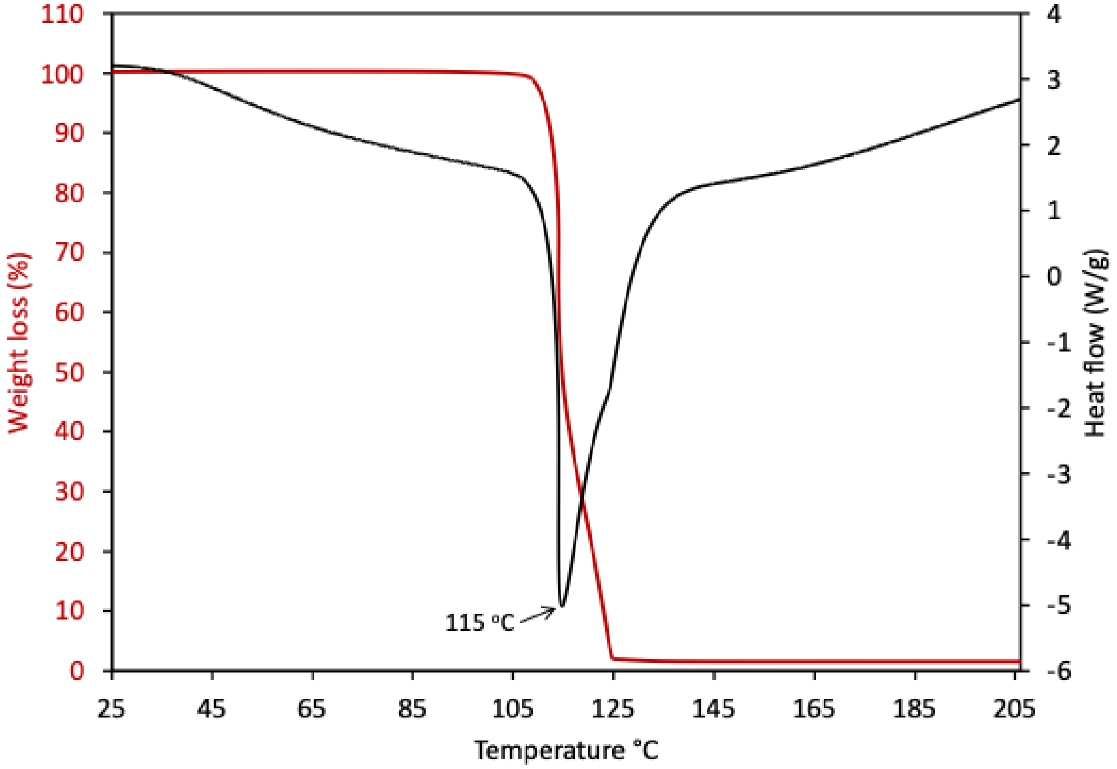

Fig. S43. Combined TGA/DSC of a sample of *exo*-**1** heated at 5 °C per minute, showing a melting endotherm at 115 °C.

S10. Computational Details

S10.1 Crystal Structure Prediction Workflow

Our crystal structure prediction method has been described previously. In brief, our quasi-random structure generation procedure generates trial structures sampling over the possible crystalline degrees of freedom (molecular positions and orientations, lattice parameters), subject to space group symmetry constraints, using a low-discrepancy (Sobol) sequence.[6] The structures thus generated are then energy minimised subject to the rigid-molecule constraint using the DMACRYS package.[7] 10,000 successfully-minimised structures were generated in each of the 25 most common space groups observed for organic crystal structures in the Cambridge Structural Database.

The energy model employed in this work is a combination of two terms – electrostatic interactions and an empirical repulsion-dispersion potential. The electrostatic interactions are modeled using fixed atom-centred distributed multipoles obtained using GDMA,[8,9] derived from the charge density of the gas-phase optimised molecule computed using molecular DFT as implemented in Gaussian.[10] The repulsion-dispersion used is the *exp*-6 Williams 99 potential[11-13] revised by Pyzer-Knapp *et al.*[14] The application of our workflow to the generated crystal structures requires each to have completed two sequential minimisations – an initial minimisation with a large external pressure applied to avoid poorly-packed structures with unphysical densities and packing arrangements, and a subsequent second minimisation with no external pressure to allow fine-tuning of the crystal packing.

Duplicate structures resulting from our CSP workflow are identified and removed by comparison of computed PXRD patterns.

S10.2 Filtering of plausible structures based on PXRD matching

Having identified a large number of possible crystal structures of *endo*-**1** with plausibly low lattice energies to be potential candidates for the true structure, we applied our PXRD comparison method using the residual PXRD pattern obtained from the experimental work, as described in the text, and our constrained dynamic-time warping comparison algorithm.[15]

The PXRD comparison between the residual pattern and computed patterns was performed for every CSP structure in the lowest 20 kJ·mol^{-1} on the CSP landscape. Our selection criteria were to accept as relevant any predicted structure that was among the 10 closest matches (smallest DTW distance) to the residual pattern across values of the DTW constraint ranging from 0.1 to 1.0 degrees. This represented a compromise between selecting the “best” candidate at one specific level of comparison and selecting candidates that were consistently good (even if not optimal) across a range of levels of comparison of varying stringency. This procedure reduced the number of structures considered plausible as matches to the experimental *endo*-**1** form from 643 before PXRD comparison to 25.

Having reduced the number of plausible CSP structures based on the residual PXRD pattern, we then optimised this smaller set of structures using periodic DFT as implemented in VASP,[16–19] using the PBE functional with the Grimme D3 dispersion correction[20] and Becke-Johnson damping.[21] Structures were again optimised using a two-stage procedure – an initial phase in which lattice vectors are fixed (to relax only atomic positions) and a second phase in which all degrees of freedom are relaxed (both lattice vectors and atomic positions) to fully refine the structures. Both stages employed a plane-wave basis energy cut-off of 500 eV and a *k*-point spacing of 0.05 Å^{-1}. A final single-point calculation at a higher plane-wave energy cut-off of 600 eV was performed to obtain more accurate energy rankings for each structure. In all calculations, the projector-augmented wave (PAW) method was employed with the standard supplied pseudopotentials.[22]

Having optimised our structures in VASP, we then re-performed our PXRD comparison to the residual pattern, finding that the second-lowest energy structure on the landscape now

represented the best match to the residual pattern across all values of the dynamic time-warping constraint. We therefore proposed this structure to be the form of solid *endo*-**1** observed experimentally, which was subsequently confirmed via single-crystal XRD.

S10.3 Free energy calculations

For our free-energy calculations on the *endo*-**1**, *exo*-**1**, and (*endo*-**1**)(*exo*-**1**) forms, we first re-optimised all the experimentally-determined crystal structures in periodic DFT using VASP as described above for the CSP results. To improve accuracy, all three crystal structures were re-optimised with an increased plane-wave basis cut-off of 1500 eV, *i.e.,* as tightly as it was reasonably possible to converge these structures.

We then took these tightly-converged minima and performed DMACRYS phonon calculations on these, as these are much lower in computational cost than periodic DFT phonon calculations. We again employed atom-centred distributed multipoles derived from the molecular charge densities (now constrained to preserve the in-crystal molecular geometry) and the W99rev potential as before. The DMACRYS phonon calculations first involved a rigid-molecule optimisation of the crystal structures (with the molecular geometry fixed at the geometry from the VASP re-optimisation), followed by numerical evaluation of the second derivatives with respect to molecular translations and rotations.

Our free-energy method has been described previously.[23] In brief, it samples *k*-points through the construction of mutually co-prime supercells. Further, it employs the Debye approximation to describe the acoustic (low-frequency) phonons to improve convergence of the calculated properties with respect to the *k*-point sampling, and also a kernel-density estimator (KDE) to model the dispersion (broadening) around each frequency in the phonon density of states (DOS). Having calculated the phonon spectra for each solid form, we then determined the zero-point energy (ZPE) along with calculating the thermal contribution at a range of temperatures from 0 K (*i.e.,* just ZPE) to 500 K. The quantity calculated is the Helmholtz free energy, *i.e.,*

$$A(T) = E_{\text{latt}} + F_{\text{vib}}(T)$$

where E_{latt} is the static (zero temperature) lattice energy and $F_{\text{vib}}(T)$ is the sum of ZPE and the finite temperature contributions calculated *via* Debye approximation and phonon DOS.

The difference in DFT total energies of the crystal structures of *endo*-1 and *exo*-1 were partitioned into intra- and inter-molecular contributions by extracting the molecular geometries from the optimised crystal structures and performing single-point energy calculations of the molecule in a 50x50x50 Å cubic box, within VASP with the same functional and basis set as the crystal structure optimisations. The intramolecular energies were taken from these single-point energy evaluations. Inter-molecular interactions in the crystals were evaluated by subtracting the intra-molecular energies from the total crystal energies.

S11. References

1 CrysAlisPro (2015) Version 1.171.38.43. (Rigaku Corporation, Tokyo).
2 C. B. Hübschle, G. M. Sheldrick, B. Dittrich, *J. Appl. Cryst.*, 2011, **44**, 1281–1284.
3 H. M. Rietveld, *J. Appl. Crystallogr.* 1969, **2**, 65–71.
4 A. A. Coelho, *J. Appl. Crystallogr.* 2018, **51**, 210–218.
5 M. J. Cliffe, A. L. Goodwin, *J. Appl. Cryst,* 2012, **45**, 1321–1329.
6 D. H. Case, J. E. Campbell, P. J. Bygrave, G. M. Day, *J. Chem. Theory Comput.*, 2016, **12**, 910–924.
7 S. L. Price, M. Leslie, G. W. A. Welch, M. Habgood, L. S. Price, P. G. Karamertzanis, G. M. Day, *Phys. Chem. Chem. Phys.*, 2010, **12**, 8478-8490.
8 A. J. Stone, M. Alderton, *Molecular Physics,* 2002, **100**, 221–233.
9 A. J. Stone, *J. Chem. Theory Comput.*, 2005, **1**, 1128–1132.
10 M. J. Frisch, G. W. Trucks, H. B. Schlegel, G. E. Scuseria, M. A. Robb, J. R. Cheeseman, G. Scalmani, V. Barone, G. A. Petersson, H. Nakatsuji, X. Li, M. Caricato, A. Marenich, J. Bloino, B. G. Janesko, R. Gomperts, B. Mennucci, H. P. Hratchian, J. V. Ortiz, A. F. Izmaylov, J. L. Sonnenberg, D. Williams-Young, F. Ding, F. Lipparini, F. Egidi, J. Goings, B. Peng, A. Petrone, T. Henderson, D. Ranasinghe, V. G. Zakrzewski, J. Gao, N. Rega, G. Zheng, W. Liang, M. Hada, M. Ehara, K. Toyota, R. Fukuda, J. Hasegawa, M. Ishida, T. Nakajima, Y. Honda, O. Kitao, H. Nakai, T. Vreven, K. Throssell, J. A. Montgomery, Jr., J. E. Peralta, F. Ogliaro, M. Bearpark, J. J. Heyd, E. Brothers, K. N. Kudin, V. N. Staroverov, T. Keith, R. Kobayashi, J. Normand, K. Raghavachari, A. Rendell, J. C. Burant, S. S. Iyengar, J. Tomasi, M. Cossi, J. M. Millam, M. Klene, C. Adamo, R. Cammi, J. W. Ochterski, R. L. Martin, K. Morokuma, O. Farkas, J. B. Foresman, and D. J. Fox, Gaussian, *Inc*., Wallingford CT, 2016.
11 D. E. Williams, *J. Mol. Struct.* 1999, **485-486**, 321–347.
12 D. E. Williams, *J. Comp. Chem.*, 2001, **22**, 1154–1166.
13 D. E. Williams, *J. Comp. Chem.*, 2001, **22**, 1–20.
14 E. O. Pyzer-Knapp, H. P. G. Thompson, G. M. Day, *Acta Crystallogr. B Struct. Sci. Cryst. Eng. Mater.*, 2016, **72**, 477–487.
15 P. Cui, D. P. McMahon, P. R. Spackman, B. M. Alston, M. A. Little, G. M. Day, A. I. Cooper, *Chem. Sci.*, 2019, **10**, 9988–9997.
16 G. Kresse, J. Hafner, *Phys. Rev. B,* 1993, **47**, 558–561.
17 G. Kresse, J. Hafner, *Phys. Rev. B,* 1994, **49,** 14251–14269.
18 G. Kresse, J. Furthmüller, *Comp. Mat. Sci.,* 1996, **6**, 15–50.
19 G. Kresse, J. Furthmüller, *Phys. Rev. B,* 1996, **54**, 11169–11186.
20 S. Grimme, J. Antony, S. Ehrlich, H. Krieg, H., 2010, *J. Chem. Phys.* **132**, 154104.
21 S. Grimme, S. Ehrlich, L. Goerigk, 2011, *J. Comp. Chem.* **32**,1456–1465.
22 G. Kresse, D. Joubert, *Phys. Rev. B,* 1999, **59**, 1758–1775.
23 J. Nyman, O. S. Pundyke, G. M. Day, *Phys. Chem. Chem. Phys.*, 2016, **18**, 15828–15837.
